# Identification of genes associated with the astrocyte-specific gene *Gfap* during astrocyte differentiation

**DOI:** 10.1038/srep23903

**Published:** 2016-04-04

**Authors:** Kenji Ito, Tsukasa Sanosaka, Katsuhide Igarashi, Maky Ideta-Otsuka, Akira Aizawa, Yuichi Uosaki, Azumi Noguchi, Hirokazu Arakawa, Kinichi Nakashima, Takumi Takizawa

**Affiliations:** 1 Department of Pediatrics, Graduate School of Medicine, Gunma University, 3-39-22 Showa-machi, Maebashi, Gunma 371-8511, Japan; 2 Stem Cell Biology and Medicine, Department of Stem cell Biology and Medicine, Graduate School of Medical Sciences, Kyushu University, 3-1-1 Maidashi, Higashi-ku, Fukuoka, 812-8582, Japan; 3 Life Science Tokyo Advanced Research Center (L-StaR), Pharmacy and Pharmaceutical Science, Hoshi University, 2-4-41 Ebara, Shinagawa-ku, Tokyo 142-5801, Japan

## Abstract

Chromosomes and genes are non-randomly arranged within the mammalian cell nucleus, and gene clustering is of great significance in transcriptional regulation. However, the relevance of gene clustering and their expression during the differentiation of neural precursor cells (NPCs) into astrocytes remains unclear. We performed a genome-wide enhanced circular chromosomal conformation capture (e4C) to screen for genes associated with the astrocyte-specific gene glial fibrillary acidic protein (*Gfap*) during astrocyte differentiation. We identified 18 genes that were specifically associated with *Gfap* and expressed in NPC-derived astrocytes. Our results provide additional evidence for the functional significance of gene clustering in transcriptional regulation during NPC differentiation.

An increasing amount of evidence supports the importance of spatial organization of the genome in the nuclei of higher eukaryotes^1^. Chromosomes and genes are non-randomly arranged and occupy preferential positions within the nucleus^2^. Moreover, these arrangements are associated with gene regulation because the sub-nuclear positions of genes change along with alterations in their transcriptional states^3^,^4^,^5^,^6^. For instance, in naïve CD4^+^T helper cells, there is an inter-chromosomal association between the regulatory region of the TH2 cytokine locus and the interferon γ (*Ifng)* promoter region, where both are repressed^7^. On the other hand, in erythroid cells, Klf1-regulated genes including globins preferentially associate at a limited number of transcriptional factories containing high levels of Klf1 once activated^8^. Other observations based on chromosome conformation capture (3C) and its derivative techniques (4C, 5C, ChIA-PET) have shown that gene associations play roles in transcriptional regulation^9^,^10^,^11^,^12^. These techniques are essential for revealing three-dimensional information regarding the spatial proximity of DNA within the cell nucleus^13^,^14^.

Neural precursor cells (NPCs) in the central nervous system can self-renew and differentiate into neurons mid-gestation, and then into astrocytes and oligodendrocytes only after late-gestation^15^. Differentiation of NPCs is temporally and spatially regulated by several factors including cytokines and epigenetic modifications^16^,^17^. NPCs from mouse telencephalon at late gestation (e.g., embryonic day [E] 14.5) are competent to differentiate into astrocytes upon stimulation with leukemia inhibitory factor (LIF)^18^,^19^. LIF activates the transcription factor STAT3, which then binds to the promoter of an astrocyte specific gene, glial fibrillary acidic protein *(Gfap)*, to induce its expression^19^,^20^. This is of great relevance in astrocytogenesis, since mice lacking a common receptor for LIF, gp130, are largely devoid of *Gfap*-positive astrocytes. These astrocytes show a lower ability to support the survival of neurons^21^. In addition, DNA demethylation and chromatin remodeling in STAT3 binding motifs on the *Gfap* promoter are essential for *Gfap* expression^22^.

*Gfap* gene loci have been shown to undergo a shift toward a more internal location upon transcriptional activation^6^. Furthermore, genomic regions adjacent to nuclear lamina are replaced as gene expression programs change during astrocyte differentiation from NPCs^23^. This indicates robust conversion of genome localization during astrocytogenesis; however, little is known about the relevance of gene clustering in NPC differentiation.

In this study, we screened for genes that associate with *Gfap* during the astrocyte differentiation of NPCs by using enhanced circular chromosome conformation capture with minor modifications (modified e4C). We looked for a correlation between gene clustering and transcriptional activities by comparing data from modified e4C and expression arrays. We identified 18 genes associated with *Gfap* that are also expressed specifically in LIF-induced astrocytes. DNA florescence *in situ* hybridization (FISH) confirmed the clustering of some genes and *Gfap*. These findings support the possibility that the association of co-expressing genes is involved in astrocyte differentiation.

## Results

### Genome-wide screening of genes specifically associated with *Gfap* and expressed in NPC-derived astrocytes

**Figure 1:**
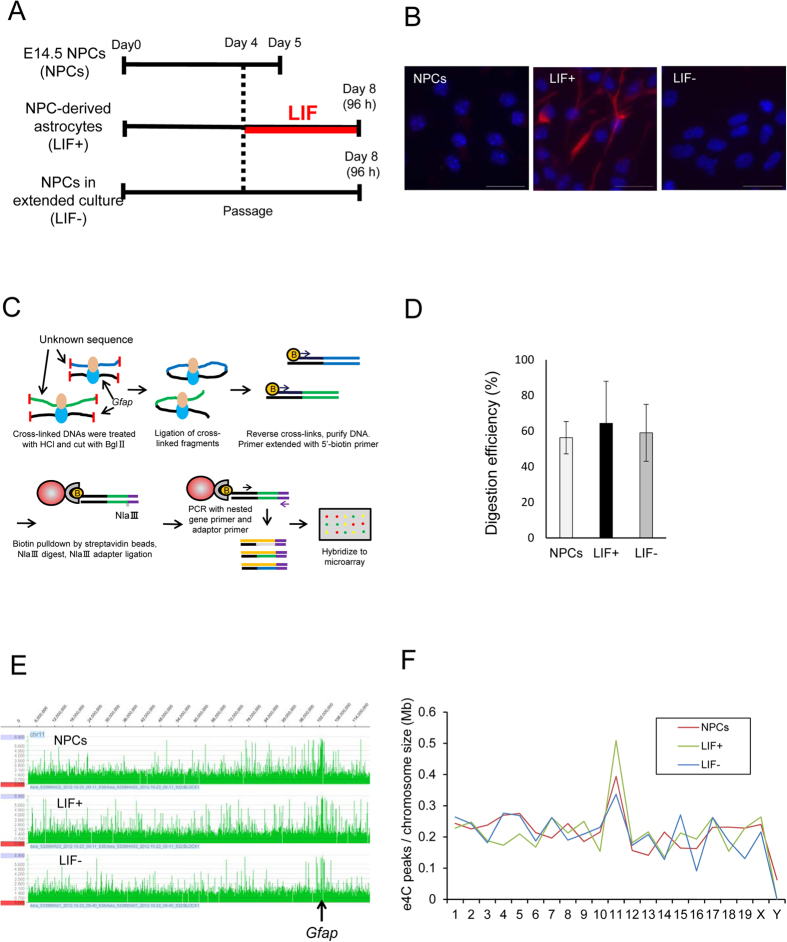
Genome-wide interactions of the *Gfap* loci in NPCs, LIF+, and LIF− cells. (**A**) Schematic experimental protocol. NPCs isolated from E14.5 mouse telencephalon were cultured and replated on day 4. On day 5, cells were used for experiments as NPCs. NPC-derived astrocytes and NPCs in extended culture were collected after an additional 4 days of culture with or without LIF. On day 8, the cells were used for experiments as LIF+ or LIF− cells. (**B**) NPCs, LIF+, and LIF− cells were stained with an anti-*GFAP* antibody (red, *GFAP*; blue, DAPI). Scale bar = 20 μm. (**C**) Schematic representation of the modified e4C method. Chromatin was fixed in paraformaldehyde and treated with 0.1 N hydrochloric acid, then digested with *Bgl*II and ligated. The resulting hybrid molecules were used as a template for the primer extension reaction using the bait-specific primer. This was followed by adaptor ligation, nested PCR, labelling with fluorescent dye, and hybridization to a custom microarray. (**D**) The digestion efficiency of the DNA samples used for modified e4C. Relative amounts of PCR products on a *Bgl*II recognition site located near the *Gfap* STAT3 binding site (GSBS) are shown. (**E**) Association profiles were determined as the signal ratio of e4C samples to reference genomic DNA. Log_2_ (e4C DNA/genomic DNA) = 2 was set as a cut-off value. (**F**) Number of e4C peaks on each chromosome. Chromosome sizes were obtained from the Mouse Genome Browser Gateway (NCBI37/mm9).

As a first step toward identifying genes clustered with and regulated similarly to *Gfap* during astrocyte differentiation, we decided to perform a modified e4C assay with a few modifications^8^. NPCs derived from E14.5 mouse brains can differentiate into astrocytes after being cultured *in vitro* for more than 4 days in the presence of the astrocyte-inducing cytokine LIF^19^. We isolated neuroepithelial cells from the telencephalon of E14.5 mice and cultured them for 5 consecutive days (designated as NPCs). After one passage, the NPCs were further cultured for 4 days with LIF to differentiate them into astrocytes (designated as LIF+ cells) (Fig. 1A). As reported previously, under these conditions, ~20% of NPCs differentiate into astrocytes as judged by immunofluorescence labeling of the astrocyte marker *GFAP* (Fig. 1B)^6^,^19^. The NPCs grown in extended culture without LIF (LIF− cells) were also tested as a control (Fig. 1A,B).

As “bait” for the e4C assay, we used a genomic region containing a STAT3 cognitive sequence on the *Gfap* promoter, the *Gfap* STAT3-binding site (GSBS). The GSBS is located ~1.5 kb upstream of the transcription start site and is a prerequisite for *Gfap* transcription during astrocytogenesis from NPCs^19^. We first tried *Bgl*II digestion of the flanking regions of GSBS. However, the digestion efficiency in this region as assessed with quantitative PCR was much lower in cells that did not express *Gfap* than those that did express *Gfap* (20.8% vs. 61.3%). We assumed the insufficient digestion was due to highly compacted chromatin around the GSBS in those cells^22^. To improve accessibility of restriction enzymes to the chromatin, we added an extra step of hydrochloric acid treatment to the original e4C protocol (Fig. 1C). Indeed, this achieved comparable digestion efficiency at the GSBS region in different types of cells (Fig. 1D) and helped to identify a large number of e4C peaks in both *cis* and *trans* in two biological replicates. As expected, many peaks were found around the bait locus, and the ratio between the e4C peak number and chromosome size was the highest for chromosome 11, which encodes *Gfap* in all the types of cells tested (Fig. 1E,F).

**Figure 2:**
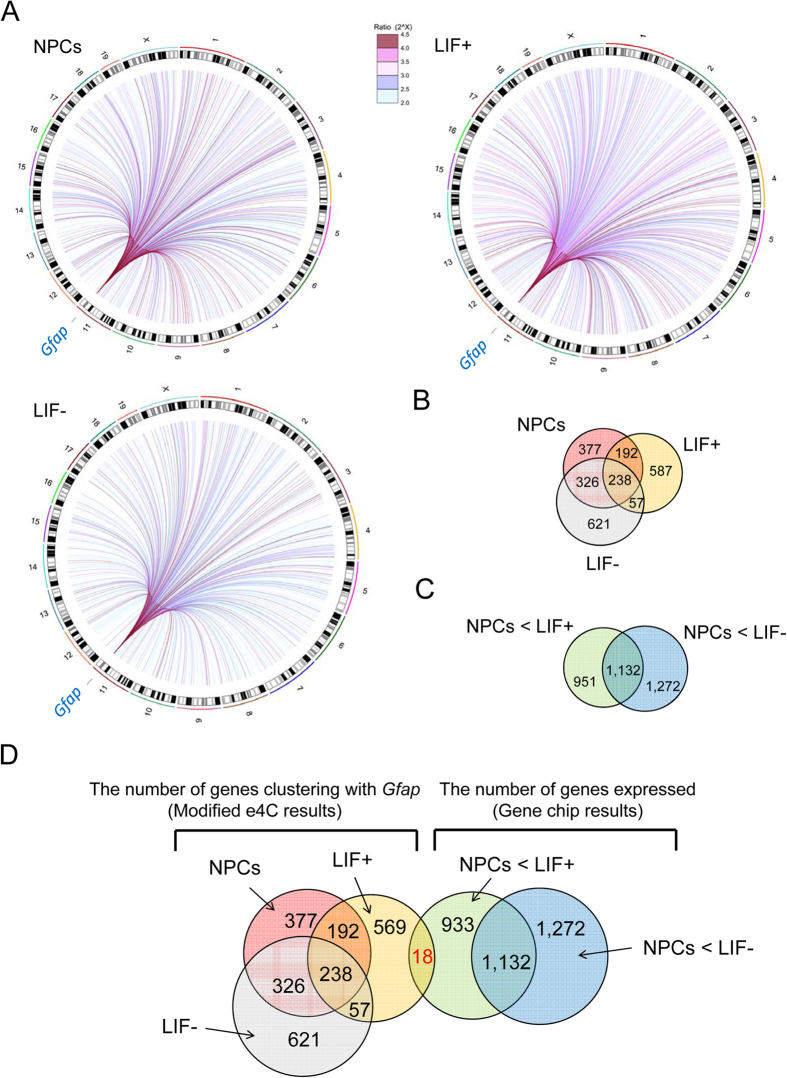
Identification of putative genes specifically associated with *Gfap* and expressed in LIF+ cells. (**A**) Circos plots showing interactions between the *Gfap* locus and interacting partners, with each line representing an interaction. Chromosomes are plotted along the circle. These plots were generated using the R package. The results of NPCs, LIF+, and LIF− cells are shown separately. Each line color represents the ratios of intensities of peaks compared to genomic controls. (**B**) Venn diagram of the *Gfap*-associating *Bgl*II fragments from e4C results in NPCs, LIF+, and LIF− cells. (**C**) Venn diagram of the genes expressed more highly in either LIF+ or LIF− cells than NPCs. (**D**) Venn diagram of 587 genes specifically associated with *Gfap* in LIF+ cells and 946 genes specifically expressed in LIF+ cells.

Trans-chromosomal interactions were also found in all types of cells tested (Fig. 2A). A number of positive probes were not replicated between two biological repeats (Figure S1A); this might be partly due to a resolution difference between the 3C assay and microarray probes. The former sometimes has cytological but not necessarily molecular resolution^24^, while the arrays represent molecular resolution and contain a probe or two for most target fragments between *Bgl*II and *Nla*III with expected lengths within 200 bp (Figure S1B). Therefore, we only focused on replicated positive probes. The analysis of distances of positive probes from the nearest genes revealed that probes 10–30 kbp away from the transcriptional start or end sites were enriched with e4C compared to all probes on the array (Figure S2). Indeed, as many as ~1,000 genes were found within 50 kbp from the positive probes that we defined as being associated with *Gfap* in each cell type (Fig. 2B and Supplemental Table S2), likely because of the relatively lower resolution of the 3C or its derivative assay^24^. Among them, 587 genes were associated with *Gfap* exclusively in LIF+ cells (Fig. 2B). We also found considerable overlap (564 genes) between NPCs and LIF− cells but less overlap between NPCs and LIF+ cells (430 genes) or LIF+ and LIF− cells (295 genes). These results show distinct associations between *Gfap* and other genes in LIF+ cells versus NPCs and LIF− cells.

To identify genes that are simultaneously associated with *Gfap* gene loci and expressed specifically in LIF+ cells, gene expression profiles in each cell type were examined by Affymetrix GeneChip analysis. To normalize gene expression levels from different samples, we adopted the Percellome method, which provides “per cell” readouts in copy numbers of mRNA by adding a set of external spike mRNAs in proportion to the DNA content as a substitute for the measurement of cell numbers in the sample^25^. We found 2,083 genes in LIF+ cells and 2,404 in LIF− cells, with more than a two-fold increase in expression in NPCs. There were 1,132 overlapping genes between LIF+ and LIF− cells and 951 genes exclusively expressed in LIF+ cells (Fig. 2C and Supplemental Table S3). By referring to genes associated in LIF+ cells in the e4C assay, we found 18 genes that were associated with *Gfap* and expressed at particularly high levels in LIF+ cells (Table 1 and Fig. 2D).

### Validation of GeneChip array and modified e4C results

**Figure 3:**
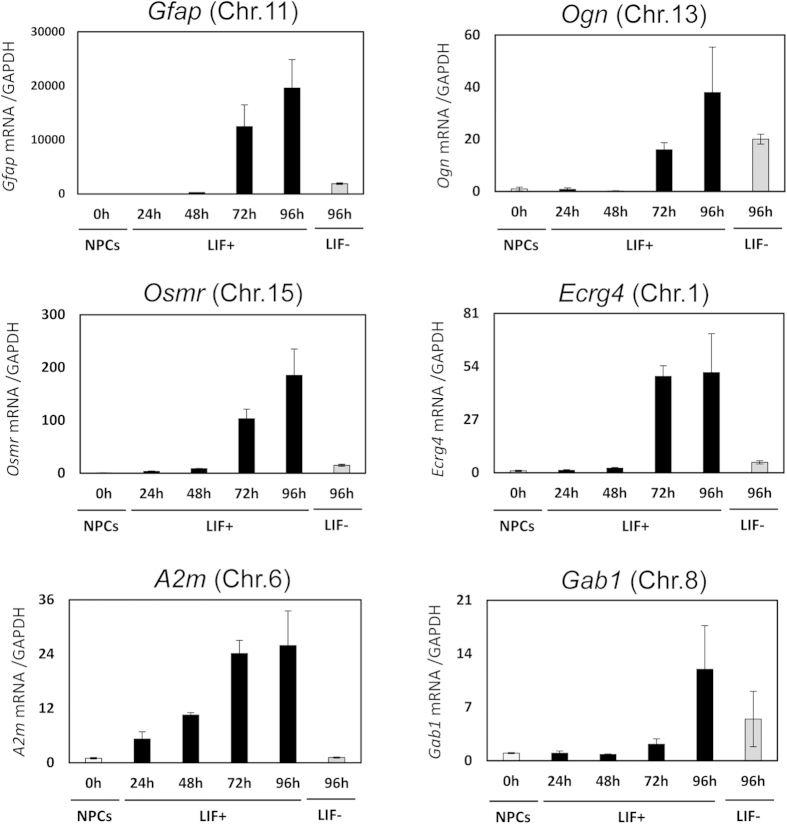
Validation in expression of *Gfap* and five putative genes identified by Genechip and modified e4C. Quantitative RT-PCR was performed on *Gfap* and five genes selected from modified e4C. The expression level of each gene was determined in NPCs (NPCs 0 h), cells stimulated with LIF for different periods of time (LIF+ 24 h, 48 h, 72 h, 96 h), and without LIF (LIF− 96 h). The results were normalized to *Gapdh* expression. Each graph represents the mean (±SEM) relative amount (compared with NPCs) in at least three experiments.

Among the putative 18 genes, we selected *Ogn*, *Osmr*, *Ecrg4*, *A2m*, and *Gab1* for further analysis because they have been implicated as playing important roles in the central nervous system^26^,^27^,^28^,^29^,^30^. To affirm the results of the GeneChip analysis, we tested the expressions of the five genes and *Gfap* by RT-qPCR. As expected, mRNA expression levels of the six genes were all significantly increased 72 and 96 h after LIF stimulation (Fig. 3).

**Figure 4:**
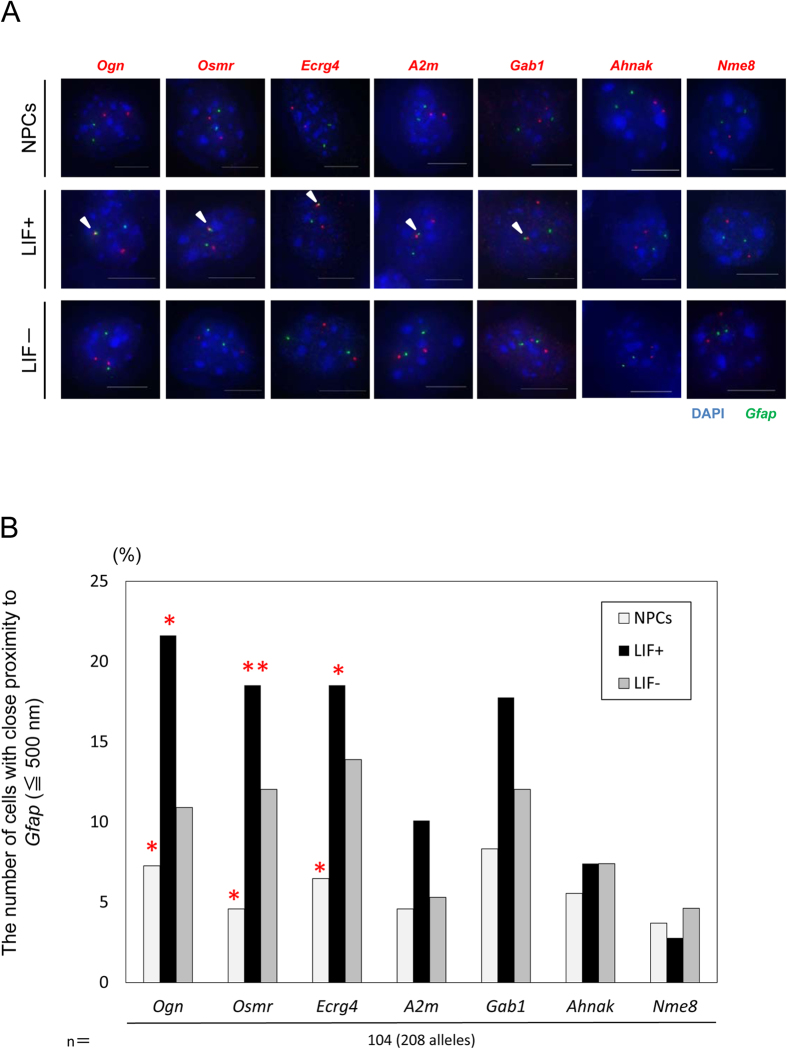
Validation of gene association between *Gfap* and five putative genes identified by Genechip and modified e4C. (**A**) Projected images of double-labeled DNA FISH in NPCs, LIF+, and LIF− cells for *Gfap* (green) and other genes (red). Nuclei were counterstained with DAPI (blue). Scale bar = 5 μm. (**B**) Association frequencies determined with DNA FISH for the indicated gene pairs in NPCs, LIF+, and LIF− cells. **P < 0.01, *P < 0.05. (**C**) Nuclear size as measured by DAPI staining in NPCs, LIF+, and LIF− cells. **P < 0.01, *P < 0.05.

The long-range interactions identified by 4C technology need to be verified by completely independent methods such as FISH because 4C technology shows average chromosome conformations from millions of nuclei^31^,^32^. Therefore, we next performed DNA FISH for each of the five putative associating genes (Fig. 4A and Figure S3). In addition to these five genes, we also tested genes that were negative in modified e4C and located on different chromosomes, namely *Ahnak* on chromosome 19 and *Nme8* on chromosome 13. The GeneChip analysis showed that *Ahnak* is exclusively expressed in LIF+ cells, while *Nme8* is expressed at miniscule levels in NPCs, LIF+, and LIF− cells. Three genes (*Ogn*, *Osmr*, and *Ecrg4*) out of the five putative genes show a significant increase in the number of cells with associated alleles in the LIF+ culture (Fig. 4B). Although not significant, similar tendencies were observed for *A2m* and *Gab*1. The number of cells with close proximity to *Gfap* did not change significantly for *Ahnak* and *Nme8* in different types of cells. Overall, there were no significant differences in the distribution of distances between *Gfap* and those gene loci among the three types of cells (Figure S4). We did notice a consistent difference between NPCs and LIF− in the association of each pair of probes (Fig. 4B). This was not explained by the nuclear diameter because it was not significantly different between NPCs, LIF+, and LIF− cells (10.4 ± 1.5 μm in NPCs, 10.1 ± 1.1 μm in LIF+, and 10.2 ± 1.2 μm in LIF−). LIF− is an extended culture of NPCs, which readily differentiate into astrocytes with changes in epigenetic programs dedicated to astrocytes^19^,^33^. This might explain the constant difference between NPCs and LIF−. Consequently, these cell-based analysis results agree with the e4C findings.

### Detailed gene association analysis

**Figure 5:**
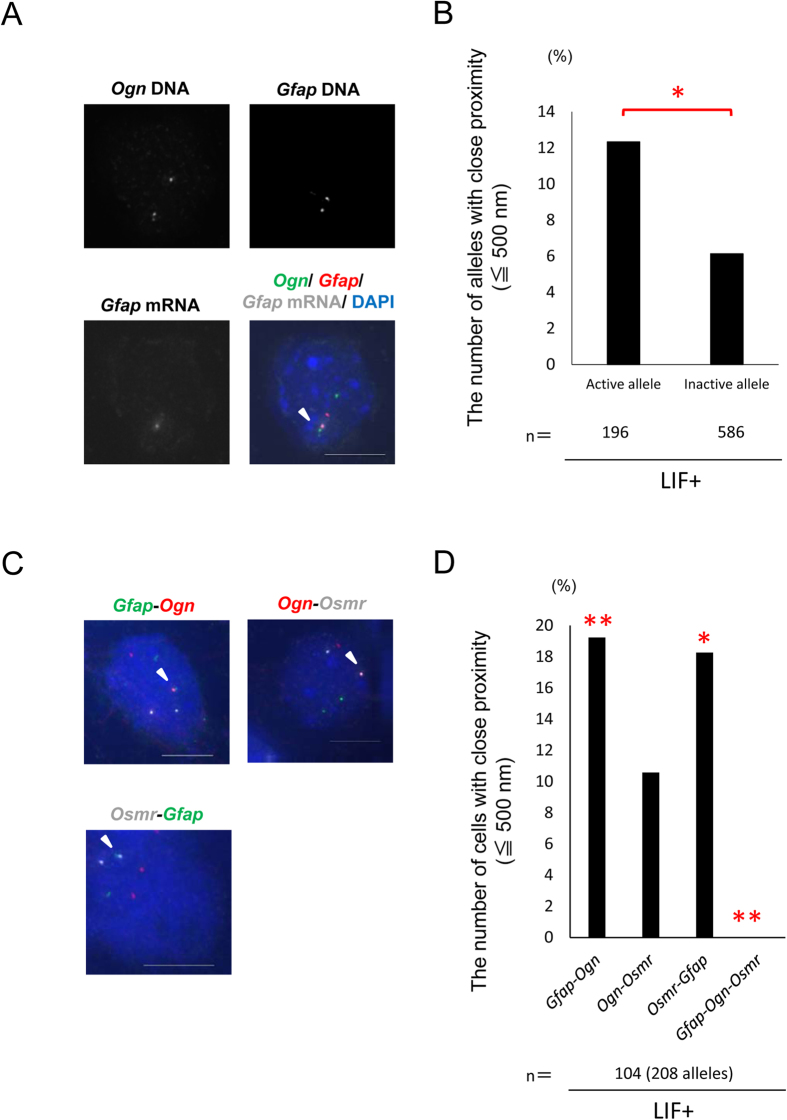
Detailed gene association analysis. (**A**) Projected images of triple-labeled RNA/DNA FISH in LIF+ cells for *Ogn* DNA (green), *Gfap* RNA (red), and *Gfap* DNA (white). Nuclei were counterstained with DAPI (blue). Scale bar = 5 μm. (**B**) Association frequencies of *Gfap* active alleles and inactive alleles determined with RNA/DNA FISH for LIF+ cells. *P < 0.05. (**C**) Projected images of triple-labeled DNA FISH in LIF+ cells for *Gfap* (green), *Ogn* (red), and *Osmr* (white). Nuclei were counterstained with DAPI (blue). Scale bar = 5 μm. (**D**) Association frequencies determined with DNA FISH for the indicated gene pairs in LIF+ cells. **P < 0.01, *P < 0.05.

We previously reported that *Gfap* is expressed in a random monoallelic manner in LIF+ cells and cortical astrocytes^6^. Hence, we investigated whether the alleles that associate are preferentially expressed with a simultaneous RNA/DNA-FISH assay. We used a probe targeting mature *Gfap* transcripts and probes targeting *Gfap* and *Ogn* gene loci. A significantly larger number of active *Gfap* alleles associated with *Ogn* loci than inactive ones (Fig. 5A,B). The results suggest that *Gfap* transcriptional activity is at least partially correlated with gene association.

According to the position weight matrix of STAT3 derived from the previous ChIP-seq data^34^, there are STAT3 binding motifs within 5 kb of the transcription start sites of 14 of the 18 genes screened with the e4C (Table 1). This indicates that these genes are activated by the common transcription factor, STAT3. We therefore aimed to determine whether the genes with STAT3 binding motifs share the same locale, i.e. a transcription factory. We performed a multicolor DNA FISH assay using probes targeting *Gfap*, *Ogn*, and *Osmr*. We did not observe these three genes clustered in our system (Fig. 5C,D), indicating that these gene associations did not simultaneously occur.

In addition, a detailed analysis of the e4C results did not indicate direct interaction among the promoters of those genes; positive probes for those 18 genes were not mapped on the promoter regions (Table 1).

## Discussion

In this study, we identified 18 genes that are specifically expressed and associated with the astrocyte-specific gene *Gfap* in NPC-derived astrocytes (LIF+ cells) by comparing gene association data obtained by applying a modified e4C genome-wide screening method to genome-wide gene expression profiles obtained from a microarray analysis. Importantly, these results were reproducible with two additional independent and distinct methods: DNA FISH and RT-qPCR. We also showed that the association of these genes correlated with *Gfap* transcriptional activity (Fig. 5A,B).

Long-range chromatin interactions including gene clustering are increasingly being recognized as an important mechanism to regulate gene expression. In many instances, these multi-gene complexes are hypothesized to be organized within “transcription factories” containing RNA polymerase II (RNAPII) and other components involved in transcription^5^,^8^,^35^. Furthermore, most genes in these transcription factories are transcribed cooperatively, and some of these genes can influence each other^36^. Although we need to investigate further to reveal the functional significance of clustering, the 18 genes identified in this study may have roles in regulating *Gfap* expression and astrocyte differentiation. In fact, one of the identified genes, *Osmr*, encodes an oncostatin M (OSM) receptor known to be involved in STAT3 activation and subsequent *Gfap* expression during astrocyte differentiation^37^. The OSMR is essential for OSM, a member of the interleukin (IL)-6 family of cytokines, to activate downstream JAK-STAT signaling pathways by forming a heterodimer with the common signal transducer gp130. Interestingly, *Osmr* itself is transcriptionally activated by STAT3^38^,^39^. This suggests that STAT3 may be involved in gene associations between *Gfap* and *Osmr*, and that the association has roles in initiating enhanceosomes for *Gfap* expression and astrocyte differentiation. Another identified gene, *Ecrg4*, participates in NPC cell cycle arrest through a mechanism involving the proteasome-dependent degradation of cyclins D1 and D3^27^,^40^. Gene associations between *Gfap* and *Ecrg4* may be relevant for the appropriate timing of transcription initiation and astrocyte differentiation. On the otherhand, e4C-positive probes were not found around the STAT3 binding sites of the 14 genes that possessed them. This indicates that close proximity of those genes, but not direct association of STAT3 binding sites, may play a role in enhancing transcription. It might be interesting to perform higher molecular resolution tests such as sequential chromatin immunoprecipitation to study gene interactions.

Changes in gene clustering reflect a wide range of genome functions such as replication, imprinting, and transcription^41^,^42^,^43^. Although we revealed transcription-related gene associations of *Gfap*, transcription is not the only factor governing associations between *Gfap* and other genes. *Gfap* is monoallelically expressed in primary astrocytes and asymmetrically replicated, as are a number of other monoallelically expressed genes^6^. Furthermore, the reduction of transcription-repressive histone modifications (e.g., methylation of H3 at lysine 9 [H3K9me2, 3]) and the expansion of transcription-permissive histone modifications (e.g., methylation of H3 at lysine 4 [H3K4me3] and acetylation of H3 at lysine 9 [H3K9Ac] at GSBS) are important for *Gfap* expression^44^,^45^,^46^. These previous findings support the idea that changes in the pairing of associated genes may depend not only on transcriptional activities but also on replication timing and epigenetic modifications. In fact, during differentiation of embryonic stem cells (ESCs) to NPCs, switching of chromosomal domains between early and late replication in the S-phase results in changes in gene pairings^43^. Furthermore, proteins that bind to H3K27me3 and cause DNA methylation can enhance gene associations and change transcription states^47^. Thus, it would be interesting to investigate replication timing and genome-wide epigenetic modifications in NPCs and NPC-derived astrocytes. In this sense, it will also be interesting to identify genes that associate with *Gfap* in NPCs from mouse telencephalon at mid-gestation (e.g., E11.5) because the promoter region of *Gfap* is highly DNA methylated and H3K27 is tri-methylated to maintain a transcriptionally repressed state^19^,^48^.

Recent studies that couple 3C derivatives and deep sequencing have shown that the genome’s spatial organization is more complex than initially thought. Dixon *et al*.^49^ showed that the genome is organized into large, discrete, and self-interacting domains and termed these “topological domains.” These serve as a fundamental organizational framework of the genome because the broader organization of these topological domains is largely unchanged during differentiation, and structural changes mostly occur within domains^49^,^50^. In this study, we found both specifically associating genes and genes that stably associate with *Gfap*. Although their molecular functions and significances remain unknown, they presumably act as the boundaries of topological domain-like structures and may play a role in cell-type-specific gene associations and expression. Among several such factors that participate in the organization of a higher-order chromatin structure is the CCCTC-binding factor (CTCF), which is enriched at the boundaries of topological domains^49^. In addition, the loss of cohesin, which co-localizes with CTCF, leads to global perturbation of topological domain organization and transcriptional regulation in NPCs and NPC-derived astrocytes^51^,^52^. Cohesin is essential for gene expression in neural cells, and its dysfunction leads to Cornelia de Lange Syndrome (CdLS), which presents as congenital anomalies and mental retardation^53^,^54^. It would be interesting to map CTCF onto the genome by using chromatin immunoprecipitation in our culture system.

Several concerns regarding the 3C and its derivative techniques have been pointed out in recent publications^24^,^32^,^55^. One is that 3C ligation products largely originate from insoluble rather than soluble fractions of chromatin^56^. The results are therefore influenced by nuclear compartment or chromatin folding. Another issue is that the ability of sequences to become cross-linked and captured to distant sequences by Hi-C corresponds to the looping-out frequency from chromosome territories^57^. This indicates that the results could be affected by differences in digestion efficiency with restriction endonucleases. The modified-e4C assay with hydrochloric acid treatment used in this paper likely ameliorated this problem. Nevertheless, 3C and its derivative techniques need to be validated by completely independent methods such as FISH because the results do not always simply represent spatial proximity or molecular interaction. In addition, these methods assess millions of cells and estimate an average chromatin conformation, which prompted us to ask whether multiple identified genes simultaneously share the same nuclear locale. Three-color DNA FISH (Fig. 5C,D) showed that at least the selected three genes were rarely present in the same locale of the nucleus simultaneously. Conclusively, our results suggest that verification of results obtained with 3C and its derivatives by FISH is indispensable to give accurate insights into the nature of gene clustering. In summary, we identified genes that specifically associate with the *Gfap* gene locus and are expressed in NPC-derived astrocytes. These results suggest that transcription of one of the astrocyte-specific genes, *Gfap*, is cooperatively regulated by co-expressed genes and their regulatory factors. We provide a new framework for *Gfap* expression and astrocyte differentiation that will help uncover the precise mechanisms of *Gfap* expression and astrocyte differentiation.

## Methods

### Cell culture

Pregnant ICR mice were used to prepare NPCs. The experimental protocols described below were performed according to the animal experimentation guidelines of Gunma University. NPCs prepared from the telencephalon of ICR mice at embryonic day (E) 14.5 were cultured as previously described^19^. Briefly, the telencephalon was triturated in Hanks’ balanced salt solution by gently pipetting with 1-mL pipette tips. Dissociated cells were cultured in N2-supplemented Dulbecco’s Modified Eagle’s Medium with F12 containing 10 ng/mL basic fibroblast growth factor (bFGF; R&D Systems, Minneapolis, MN) on culture dishes (Corning, Corning, NY) that were pre-coated with poly-L-ornithine and fibronectin (Sigma-Aldrich, St. Louis, MO). For astrocyte differentiation, the cells were re-plated on fibronectin/poly-L-Lysine-coated glass coverslips (MATSUNAMI, Osaka, Japan) or culture dishes that were pre-coated with poly-L-ornithine and fibronectin after 4 d of culture and were stimulated for 4 d in the presence of LIF (50 ng/mL; Millipore, Billerica, MA). All animal procedures were conducted with the approval of Gunma University Animal Care and Use Committee and were in full compliance with the Committee’s guidelines.

### Immunocytochemistry

Cells cultured on coated glass coverslips were fixed in 4% paraformaldehyde in phosphate-buffered saline (PBS) and washed with PBS as described previously^6^. A mouse monoclonal antibody against *GFAP* (Sigma, #G-6171) was used as a primary antibody. Alexa 555- or Alexa 647-conjugated secondary antibodies (Invitrogen, Carlsbad, CA) were used for visualization. For simultaneous labeling experiments, immunostaining was performed after FISH.

### Modified e4C

e4C was performed as described previously^8^ with minor modifications. Briefly, cells were exposed to 2% formaldehyde for 10 min. Cells were collected after they were quenched with 125 mM glycine. After they were homogenized, the cells were lysed in 2 mL lysis buffer (10 mM Tris-HCl [pH 7.5], 10 mM NaCl, 0.2% NP-40, 1× protease inhibitor cocktail [Nacalai Tesque]) for 1.5 h at 4 °C and centrifuged to remove the supernatant. Extracted nuclei were treated with 0.1 N HCl for 10 min and neutralized with 0.1 N NaOH and then incubated with 850 U *Bgl*II (New England Biolabs, Ipswich, MA) overnight at 37 °C. After being inactivated in 1.6% SDS at 65 °C for 20 min, samples were diluted in 4.8 mL ligation buffer (66 mM Tris-HCl [pH 7.5], 6.6 mM MgCl_2_, 10 mM DTT, 0.1 mM ATP) and 2400 U T4 ligase (New England Biolabs) and incubated at 16 °C for 4 h. Ligated chromatin was digested by proteinase K (100 ng/mL; Merck, White House Station, NJ). After phenol-chloroform extraction, DNA was ethanol precipitated. Then, digestion efficiency was verified as previously described^58^, and the 3C products were processed for primer extension with 2 U Vent (exo-) DNA polymerase (New England Biolabs) and biotinylated bait-region–specific primers. After being bound to streptavidin-coated magnetic beads (Dynabeads M-280, Invitrogen), the biotinylated products on beads were digested with 20 U *Nla*III (New England Biolabs), followed by an adaptor ligation with 2000 U T4 ligase (New England Biolabs). The beads were used for PCR with nested bait-region–specific primers and adaptor-specific primers. The amplified products were digested again with 20 U *Bgl*II and 20 U *Nla*III (New England Biolabs). Following phenol-chloroform extraction, the e4C products were ethanol precipitated. The samples were hybridized to the customized microarray following NimbleGen’s protocol. The following primers and adapters were used: 5′ GGACATGATGAGGTCCAGTC 3′ and 5′ GCTTGCTGAGGTTCTCCTAATG 3′, 5′ GCCCACGAGTGACTCACCTTG 3′ and 5′ CCAGGATGCCAGGATGTCAG 3′ (*Gfap Bgl*II site and GSBS for Digestion efficiency check), 5′ biotin GCTTGCTGAGGTTCTCCTAATG 3′ (biotinylated bait-specific primer for primer extension), 5′ TTGGATTTGCTGGTGCAGTACAACTAGGCTTAATAGGGACATG 3′ and 5′ phosphorylated CCCTATTAAGCCTAGTTGTACTGCACCAGCAAATCC 3′ amine C7 (Nla adapter strands), and 5′ GGATTTGCTGGTGCAGTACA 3′ and 5′ GAATAATGGCATAGTGAGGGAG 3′ (for nested PCR).

### e4C microarray

e4C material was labeled and competitively hybridized with digested mouse genomic DNA as described previously^8^. The customized NimbleGen array consists of 1.4 million probes with a length of 50–70mer covering as many fragments as possible with *Bgl*II and *Nla*III sites on the 5′ and 3′ ends, respectively, and a size >100 bp based on NCBI37/mm9. Two biological replicates were performed for each condition, and reproducible positive probes were identified as e4C hits by setting a cut-off value of log_2_ (e4C signal/genomic control) = 2 (200-bp sliding window). e4C genes were identified by mapping all genes within 50 kb from each peak. Circos Plots of the results were generated with R version 3.3.3 using Package RCircos ver1.1.2.^59^

### Sample preparation and GeneChip analysis

Sample preparation and GeneChip analysis were performed as described previously^60^. The expression data were converted to copy numbers of mRNA per cell by the Percellome method, quality controlled, and analyzed using Percellome software^25^. Genes with copy numbers that increased by at least two-fold were identified as upregulated genes, while genes whose copy numbers of mRNA were <1 were excluded.

### FISH

Probes for DNA FISH were generated by nick translation of BAC clones covering genes of interest (BACPAC Resources). The following BAC clones were used: RP24–155G1 (*Gfap*), RP24–152H11 (*Ogn*), RP23–198P20 (*Osmr*), RP23–185E14 (*A2m*), RP24–214M12 (*Ecrg4*), RP24–114L21 (*Gab1*), RP23–118O2 (*Ahnak*), and RP23–211E13 (*Nme8*). FISH was essentially performed as described previously^6^. Briefly, cells were fixed with 4% PFA and kept in PBS at 4 °C until use. The cells were permeabilized with 0.5% Triton X-100/PBS and treated with 0.1 N HCl for 10 min. Cells were denatured for 30 min at room temperature in 50% formamide with 2× SSC. Hybridization was performed overnight at 37 °C with dinitrophenyl (DNP) or digoxigenin (DIG) or biotin-labeled probes and detected with Alexa 488-conjugated anti-DNP (Invitrogen) or rhodamine-conjugated anti-DIG antibody (Roche, Basel, Switzerland) or Alexa 647-conjugated streptavidin (Invitrogen). For RNA/DNA FISH, RNA probes were made as previously described^6^. In brief, *Gfap* exon sequences were amplified using cDNA prepared from astrocytes as a template. Amplified DNA was *in vitro* transcribed and then reverse-transcribed in the presence of biotin-labeled dUTP (Invitrogen). The single-stranded biotin-labeled cDNA probe was used to detect *Gfap* mRNA. Cells were fixed with 4% PFA containing 10% acetic acid and kept in 70% ethanol at −30 °C until use. Cells were digested with 0.05% pepsin/0.01 N HCl, dehydrated through ethanol treatment, and hybridized to the single-stranded DNA probe against *Gfap* cDNA. RNA-probe hybrids were detected with horseradish peroxidase-conjugated streptavidin and further labeled with Alexa598-conjugated tyramide using the TSA kit (Invitrogen). DNA FISH was performed after RNase treatment.

### Microscopy and image analysis

A DeltaVision microscope (CORNES Technologies) was used to analyze the results of DNA FISH. 3D images were obtained from serial Z-sections of 8.0-μm thickness in 0.1-μm intervals. For association analysis, the shortest distances between two FISH signals were examined by softWoRx Explorer1.3 (Applied Precision). Genes were considered associated when they were positioned within 500 nm of *Gfap*.

### Quantitative RT-PCR

Total RNAs were extracted with ToTALLY RNA^TM^ Total RNA Isolation Kit and then treated with DNaseI (Life Technologies). Complementary DNAs were synthesized from 2 μg total RNA using Superscript II (Life Technologies). Quantitative real-time PCR (qPCR) was performed in an Applied Biosystems 7900HT Fast Real-Time PCR system (Life Technologies) using the KAPA SYBR Fast qPCR Kit (Kapa Biosystems, Woburn, MA). Expression of the target genes was normalized to that of glyceraldehyde-3-phosphate dehydrogenase (*Gapdh*). The following primers were used: 5′ ATCGAGATCGCCACCTACAG 3′ and 5′ CTCACATCACCACGTCCTTG3′ (for *Gfap*), 5′ TGCAACAGGCAATTCTGAAG 3′, 5′ TGCAACAGGCAATTCTGAAG 3′ and 5′ TCCTTGGCAGTCAGCTTTTT 3′ (for *Ogn*), 5′ ACACCAAGTCCCTTCCACAG 3′ and 5′ ATGGTGACATTGGAGCCTTC 3′ (for *Osmr*), 5′ GCCTGAGGTACAGCAGTGGT 3′ and 5′ ATGGCCGCATCTTCATCATA 3′ (for *Ecrg4*), 5′ CTTCTATTATCTGATGATGGCAAAGG 3′ and 5′ CCTGCGTCACAGGCAGAAC 3′ (for *A2m*), 5′ CCAGGACGATCCACAAGACT 3′ and 5′ TTCATTCCGTGTTTGCTCTG 3′ (for *Gab1*), 5′ ACCACAGTCCATGCCATCAC 3′ and 5′ TCCACCACCCTGTTGCTGTA 3′ (for *Gapdh*).

### Statistical analyses

Residual analyses of chi-squared tests were used for Figs 4B and 5D. Fisher’s and Kolmogorov-Smirnov tests were used for Fig. 5B and Figure S4, respectively. One-way analysis of variance (ANOVA) with nonparametric tests was used to compare nuclei sizes (data not shown).

### Accession codes

Data are deposited in NCBI’s Gene Expression Omnibus and are accessible through GEO Series accession number GSE66097.

## Additional Information

**How to cite this article**: Ito, K. *et al*. Identification of genes associated with the astrocyte-specific gene *Gfap* during astrocyte differentiation. *Sci. Rep*. **6**, 23903; doi: 10.1038/srep23903 (2016).

## Supplementary Material

Supplementary Information

## Figures and Tables

**Table 1:** List of the 18 putative genes specifically associated with *Gfap* and expressed in LIF+ cells.

Accession	Gene symbol	NPCs	LIF+	LIF−	LIF+/NPCs	LIF−/NPCs	STAT3 binding site	Distance to probe region detected by e4C
NM_011019	*Osmr*	0.20	14.43	0.27	72.14	1.33	○	10–30 kb from TES
NM_008760	*Ogn*	0.10	3.46	0.04	34.58	0.42	○	10–30 kb from TSS
NM_011313	*S100a6*	1.20	29.50	1.27	24.58	1.06	○	10–30 kb from TES
NM_172471	*Itih5*	0.60	11.04	1.81	18.40	3.02	○	>30 kb from TES
NM_175459	*Glis3*	0.40	2.97	0.38	7.43	0.94	○	>30 kb from TSS
NM_008046	*Fst*	0.20	1.36	0.23	6.82	1.16	○	>30 kb from TES
NM_133832	*Rdh10*	1.20	4.97	1.76	4.14	1.47	○	5–10 kb from TSS
NM_024283	*Ecrg4*	0.50	1.76	0.08	3.52	0.16	○	>30 kb from TSS
NM_011883	*Rnf13*	2.70	9.12	5.38	3.38	1.99	○	10–30 kb from TES
NM_175628	*A2m*	11.10	37.32	4.31	3.36	0.39	○	5–10 kb from TES
BC019423	*Rsph9*	0.80	1.99	0.61	2.48	0.76	×	10–30 kb from TES
BC110634	*Galntl1*	3.00	7.42	3.95	2.47	1.32	○	>30 kb from TES
NM_009011	*Rad23b*	6.00	14.72	11.27	2.45	1.88	×	>30 kb from TES
NM_009371	*Tgfbr2*	1.00	2.33	0.29	2.33	0.29	○	>30 kb from TSS
NM_173876	*Clcn3*	3.30	7.38	6.31	2.24	1.91	○	<2 kb from TES
NM_175836	*Spnb2*	4.60	10.06	6.84	2.19	1.49	○	>30 kb from TSS
NM_008654	*Ppp1r15a*	1.00	2.14	1.34	2.14	1.34	×	>30 kb from TES
BC094659	*Gab1*	11.70	23.66	11.72	2.02	1.00	×	2–5 kb from TES

TSS; transcription start site, TES; transcription end site.
