# Supplementary material for: Identification of genes associated with the astrocyte-specific gene Gfap during astrocyte differentiation
Source: Sci Rep. 2016 Apr 4;6:23903. doi: 10.1038/srep23903 (PMC4819225; doi:10.1038/srep23903)
Supplement: Supplementary Information [file srep23903-s1.pdf]

Supplementary information

Identification of genes associated with the astrocyte-specific gene *Gfap* during astrocyte differentiation

Kenji Ito[1], Tsukasa Sanosaka[2], Katsuhide Igarashi[3], Maky Otsuka I[3], Akira Aizawa[1], Yuichi Uosaki[1], Azumi Noguchi[1], Hirokazu Arakawa[1], Kinichi Nakashima[2], Takumi Takizawa[1*]

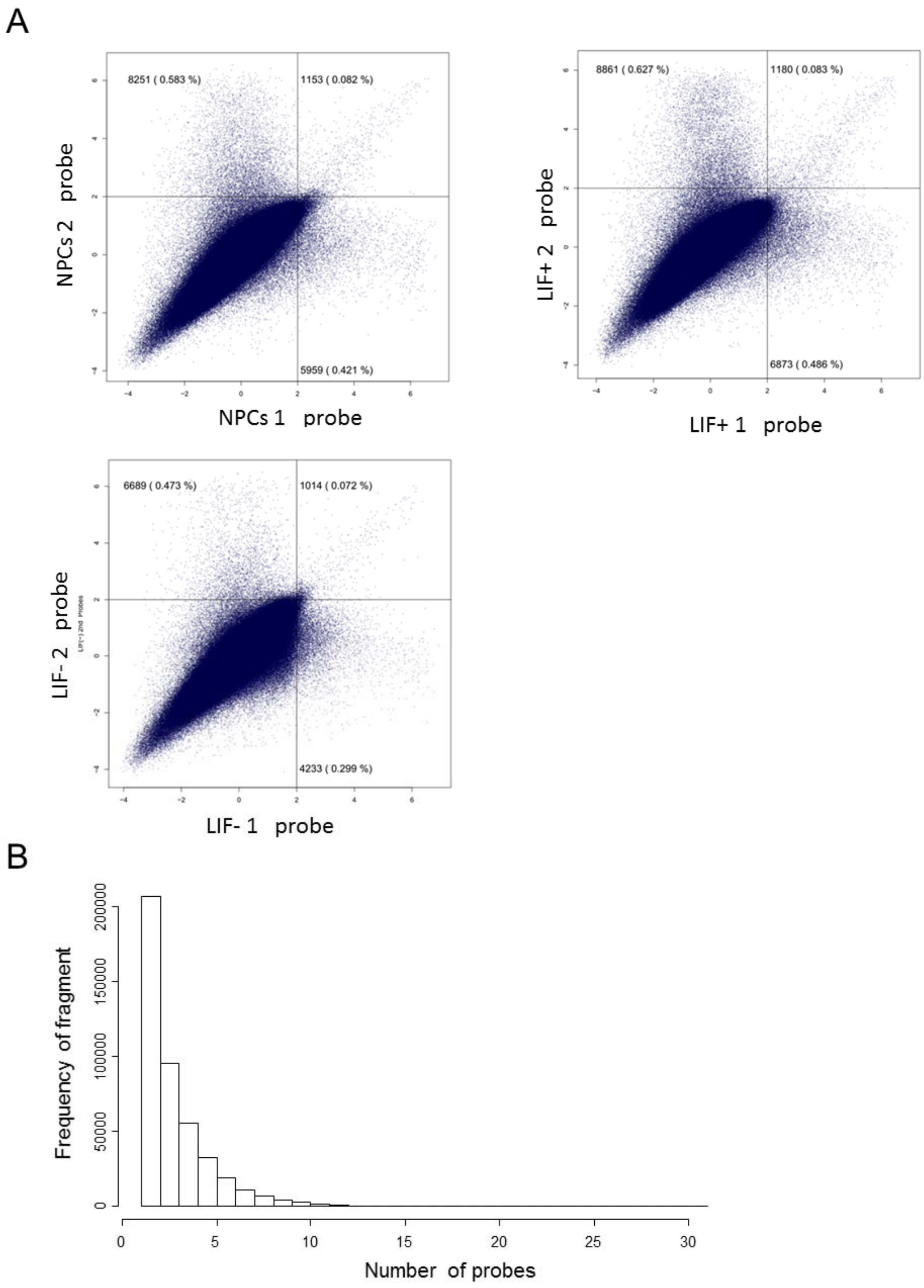

Supplemental Figure S1. Correlation plot of modified-e4C between two biological replicates and the number of probes per fragment. (A) Log_2 (e4C signal/genomic control) values of probes from two biological replicates were plotted for each cell type. (B) Relationship between the number of probes located on fragments and the frequency of fragments digested with *Bgl*II and *Nla*III.

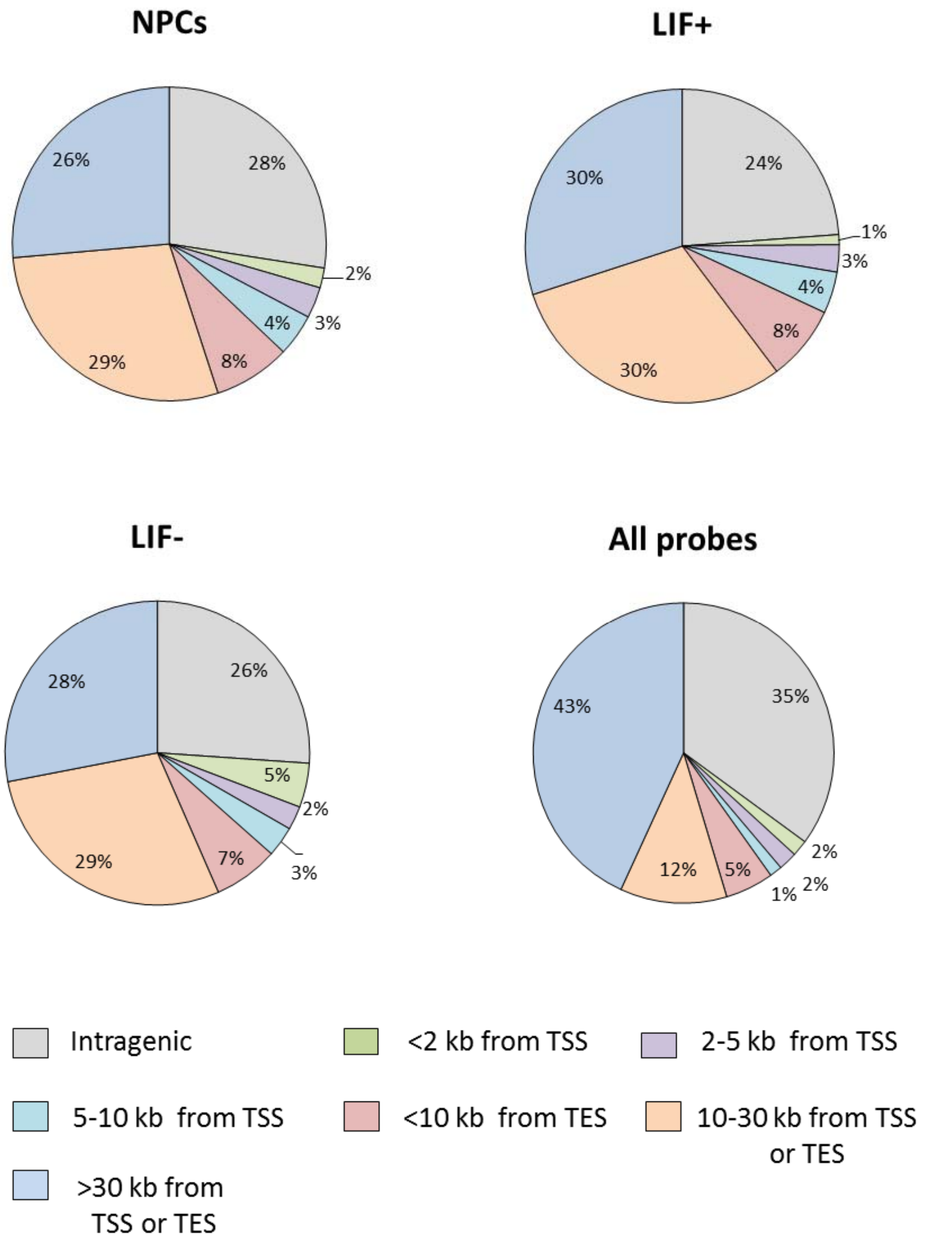

Supplemental Figure S2. Positions of positive peaks to the nearest genes in each cell type. Distances of positive peaks identified in each cell type were classified as indicated, and the percentages of different distance groups are indicated in the pie charts. All probes indicate the distances of all probes in the microarray. TSS: transcription start site, TES: transcription end site.

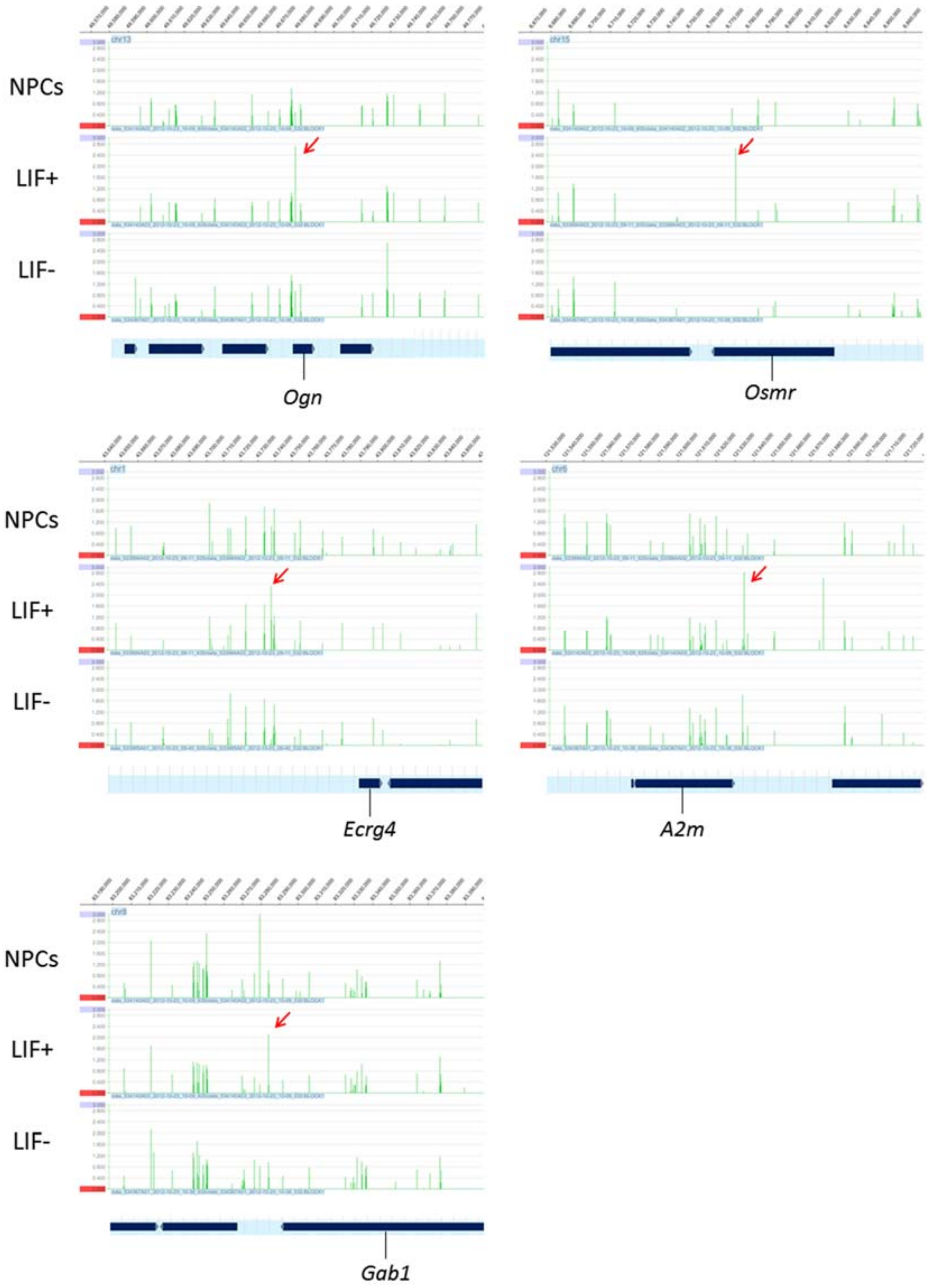

Supplemental Figure S3. Zoomed in representation of the results of modified e4C of five genes. Zoomed images of modified-e4C profiles for the ~200-kb flanking regions of *Ogn*, *Osmr*, *Ecrg4*, *A2m*, and *Gab1*. Dark-blue bars denote the positions of genes within these regions.

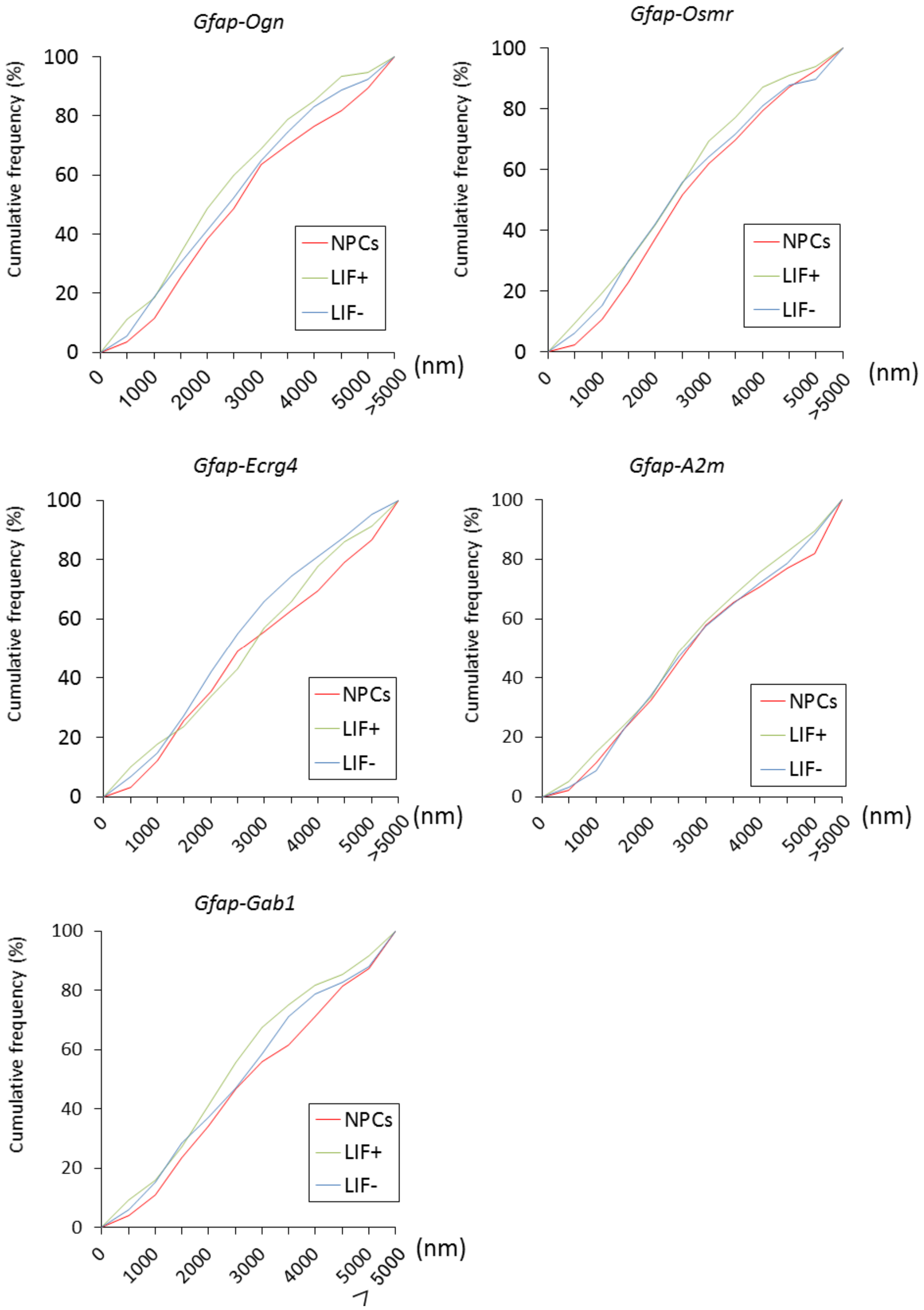

Supplemental Figure S4. Interprobe distance distributions. Cumulative frequency of alleles with indicated distances between *Gfap* and five genes.

Supplementary Table1.

List of the 238 genes that are associated with Gfap in NPCs, LIF+ and LIF-

Chr	Region Start	Region End	accession	Name	ncbi_gene_id
Chr1	10314807	10710024	NM_177834	Cpa6	329093
Chr1	14159038	14299937	BC066860	Eya1	14048
Chr1	21388488	21952023	NM_023872	Kcnq5	226922
Chr1	40637071	40687576	NM_177084	Slc9a4	110895
Chr1	44608355	44853677	BC032154	Gulp1	70676
Chr1	68086539	69154633	NM_010154	Erbb4	13869
Chr1	74438182	74443851	NM_153088	Ctdsp1	227292
Chr1	74455957	74482134	NM_009509	Vil1	22349
Chr1	74482084	74590860	NM_176972	Usp37	319651
Chr1	98715360	98768748	NM_172658	Slco4c1	227394
Chr1	98802752	98894137	NM_001039475	Slco6b1	67854
Chr1	101669341	102381276	BC167183	Cntnap5b	241175
Chr1	108434755	108610867	NM_009741	Bcl2	12043
Chr1	108616986	108656319	NM_027534	Kdsr	70750
Chr1	117581713	118477249	BC156970	Cntnap5a	636808
Chr1	123200929	123224339	NM_133748	Insig2	72999
Chr1	133693971	133713597	BC120725	Pm20d1	212933
Chr1	133724553	133745440	BC096596	Slc41a1	98396
Chr1	133763853	133769462	NM_144875	Rab7l1	226422
Chr1	133807034	133832897	NM_175294	Nucks1	98415
Chr1	153191627	153203928	NM_001039511	Ivns1abp	117198
Chr1	153214828	153275565	NM_025819	1200016B10Rik	66875
Chr1	155066051	155179916	NM_010683	Lamc1	226519
Chr1	183745446	183772532	NM_133815	Lbr	98386
Chr1	183833697	183949730	BC062927	Enah	13800
Chr1	193130670	193141985	BC048464	Nenf	66208
Chr1	193149843	193176804	NM_025864	Tmem206	66950
Chr1	193175859	193220920	NM_144880	Ppp2r5a	226849
Chr2	40452292	42509118	NM_053011	Lrp1b	94217
Chr2	53936503	53937963	NM_023396	Rprm	67874
Chr2	54288797	54970155	NM_173030	Galnt13	271786
Chr2	58298848	58368570	BC058718	Acvr1	11477
Chr2	101790357	102026534	NM_178886	Ldlrad3	241576
Chr2	135906823	136213703	NM_172858	Pak7	241656
Chr2	146681579	146795816	BC150844	Ncrna00153	228730
Chr2	149940142	149962414	NM_181266	Zfp120	104348
Chr2	150007490	150019015	NM_001145863	Gm14139	100271882
Chr2	168301909	168415783	NM_010899	Nfatc2	18019
Chr3	30993982	31021499	NM_011386	Skil	20482
Chr3	31801624	32099102	NM_028231	Kcnmb2	72413
Chr3	92120626	92122007	NM_011469	Sprr2b	20756
Chr3	92143061	92144795	NM_011470	Sprr2d	20758
Chr3	92156064	92157371	NM_011471	Sprr2e	20759
Chr3	92169108	92170364	NM_011472	Sprr2f	20760
Chr3	92177836	92179151	NR_003548	Sprr2g	20761
Chr3	99689339	99947245	NM_028892	Spag17	74362
Chr3	101380145	101408526	BC033435	Atp1a1	11928
Chr3	101616998	101640146	NM_172295	BC037703	242125
Chr3	101659695	101728376	NM_001039371	Slc22a15	242126
Chr3	107680147	107695817	NM_133867	Eps8l3	99662
Chr3	107691769	107699131	NR_015459	4933431E20Rik	329735
Chr3	107698771	107701603	NM_010360	Gstm5	14866
Chr4	45436148	45543700	NM_001033306	Shb	230126
Chr4	110070395	111330115	NM_030231	Agbl4	78933
Chr4	118293420	118300379	NM_026932	Ebna1bp2	69072
Chr4	118338123	118339070	NM_146315	Olfr62	18363
Chr4	118362066	118363068	BC119161	Olfr1342	258708

Chr4	121087339	121095704	NM_001144948	Gm12886	666921
Chr4	131776939	131817464	NM_001122992	Gmeb1	56809
Chr4	131825993	131826101	NR_002865	Rnu11	353373
Chr4	131830289	131849242	NM_025579	Taf12	66464
Chr4	131858108	131859271	NM_001081651	Rab42	242681
Chr4	131864592	131866939	NR_029468	Snhg12	100039864
Chr4	131867677	131885404	BC048840	Trnau1ap	71787
Chr4	141000587	141023852	NM_009541	Zbtb17	22642
Chr4	141023804	141094512	NM_019763	Spen	56381
Chr4	141102076	141104228	NR_030695	B330016D10Rik	320456
Chr4	143981576	143998367	NM_029948	Pramef12	77632
Chr4	144008093	144028675	NM_027056	1700012P22Rik	69364
Chr4	144043673	144053659	NM_001085503	Aadacl3	230883
Chr4	151552208	151645963	NM_133348	Acot7	70025
Chr4	151648470	151659446	NM_178406	Gpr153	100129
Chr4	151660080	151665771	NM_008237	Hes3	15207
Chr4	151671335	151681229	BC110695	Icmt	57295
Chr4	151681131	151692734	NM_001033489	Rnf207	433809
Chr5	24687297	24726107	NM_026449	Galntl5	67909
Chr5	24728710	24771736	NM_144908	Galnt11	231050
Chr5	24777611	25004601	NM_001081383	Mll3	231051
Chr5	30339700	30346508	NM_031168	Il6	16193
Chr5	30385366	30400121	NM_021288	Tyms	22171
Chr5	32438844	32460207	NM_008037	Fosl2	14284
Chr5	64551449	64742725	NM_019636	Tbc1d1	57915
Chr5	65194761	65221368	NM_008453	Klf3	16599
Chr5	128263860	128938937	NM_172885	Tmem132d	243274
Chr5	129085004	129106562	NR_015517	5930412G12Rik	319616
Chr5	129106980	129109968	NM_175284	Fzd10	93897
Chr6	45010059	47251370	NM_001004357	Cntnap2	66797
Chr6	56782052	56829352	BC043129	Fkbp9	27055
Chr6	56832394	56851862	BC038029	Nt5c3	107569
Chr6	56906459	56907493	BC132386	V1rc21	171194
Chr6	70305867	70676955	BC094049	SMUSG000000765	384419
Chr6	82352468	82510093	NM_009313	Tacr1	21336
Chr6	137683602	137700451	NM_011499	Strap	20901
Chr6	137703096	137786409	BC016218	Dera	232449
Chr7	4071134	4087843	NM_001109765	Ttyh1	57776
Chr7	4088657	4099775	NM_172736	Leng8	232798
Chr7	4099784	4101369	BC116676	Leng9	243813
Chr7	4102861	4116301	NM_021454	Cdc42ep5	58804
Chr7	16534335	16552884	NM_153068	Ehd2	259300
Chr7	16556610	16584844	NM_001081418	Gltscr1	243842
Chr7	53698530	53895156	BC049603	Sergef	27414
Chr7	68850735	69072460	NR_015533	A230057D06Rik	319893
Chr7	69088786	69127189	NR_015599	A330076H08Rik	320026
Chr7	79222715	79451481	NM_001024703	Mctp2	244049
Chr7	98239295	99597599	NM_011807	Dlg2	23859
Chr7	104100036	104213267	NM_153591	Nars2	244141
Chr7	148247172	148258016	BC172670	B4galnt4	330671
Chr7	148264127	148276408	NM_001162924	Pkp3	56460
Chr8	128519001	128554585	NM_008430	Kcnk1	16525
Chr8	65430242	65835893	NM_023689	Spock3	72902
Chr8	83223842	83263358	NM_053124	Smarca5	93762
Chr8	38585607	39724562	NM_145841	Sgcz	244431
Chr8	113370923	113406134	NM_133973	Cog4	102339
Chr8	113265729	113329803	NM_029646	Il34	76527
Chr8	113334391	113370630	BC042580	Sf3b3	101943
Chr9	34296315	34843889	NM_026324	Kirrel3	67703
Chr9	35259660	35315237	NM_021339	Cdon	57810

Chr9	64033592	64101191	BC054754	Map2k1	26395
Chr9	64083007	64084169	NM_033607	Uchl4	93841
Chr9	64129413	64152599	NM_025372	Tipin	66131
Chr9	114399433	114405662	NM_009916	Ccr4	12773
Chr9	114420392	114473487	NM_001042503	Trim71	636931
Chr9	122026383	122078818	NM_133741	Snrk	20623
Chr10	9475344	9620838	NM_001081344	Stxbp5	78808
Chr10	33871198	33927357	NM_172508	Dse	212898
Chr10	34001995	34004691	NM_009433	Tspyl1	22110
Chr10	61533769	61535319	NR_002687	Gm5424	432466
Chr10	75052125	75079713	NM_011820	Ggt5	23887
Chr10	75099363	75106753	NM_027890	Susd2	71733
Chr10	75108854	75227102	NM_172549	Cabin1	104248
Chr10	80318019	80331453	NM_175195	3110056O03Rik	73218
Chr10	80342464	80362186	BC172169	Tmprss9	432478
Chr10	80362194	80363714	NM_013895	Timm13	30055
Chr10	80364107	80380966	BC051985	Lmnb2	16907
Chr10	80392834	80394949	BC023815	Gadd45b	17873
Chr10	80411368	80464110	NM_001038655	Gng7	14708
Chr10	82082596	82085973	NM_001135569	1190007I07Rik	544717
Chr10	82092582	82113239	NM_011561	Tdg	21665
Chr11	23156040	23197013	BC062912	Xpo1	103573
Chr11	45869488	45960844	NM_009616	Adam19	11492
Chr11	45961656	45979861	NM_172524	Nipal4	214112
Chr11	46007359	46125852	NM_133769	Cyfip2	76884
Chr11	51781132	51790836	NM_029976	Cdkn2aipnl	52626
Chr11	51798648	51813968	NM_009458	Ube2b	22210
Chr11	51817722	51841061	NM_001166655	Cdkl3	213084
Chr11	75401609	75442280	NM_008850	Pitpna	18738
Chr11	75444521	75462367	NM_008916	Inpp5k	19062
Chr11	75465010	75488136	NM_008659	Myo1c	17913
Chr11	75492811	75519594	NM_133656	Crk	12928
Chr11	78207986	78219159	NR_003282	Gm11194	790911
Chr11	78210777	78235687	NM_022411	Slc13a2	20500
Chr11	78279202	78285446	NM_026740	Slc46a1	52466
Chr11	78285832	78311256	NM_001168521	Sarm1	237868
Chr11	78312621	78315826	BC018521	Vtn	22370
Chr11	102697609	102699353	NM_080846	Higd1b	75689
Chr11	102699785	102742255	BC054778	Eftud2	20624
Chr11	102742557	102746529	NM_028492	Ccdc103	73293
Chr11	102746636	102748003	BC116769	Ccdc103	66784
Chr11	102748649	102758514	NM_010277	Gfap	14580
Chr11	102766832	102786438	NM_197959	Kif18b	70218
Chr11	102800578	102807775	NM_011795	C1ql1	23829
Chr11	102855360	102878427	BC005607	Dcakd	68087
Chr11	102889872	102927417	BC021635	Nmt1	18107
Chr12	30859421	31058240	NM_172951	Sntg2	268534
Chr12	87582818	87632320	BC048169	1700020O03Rik	70373
Chr12	100955740	101121652	NM_030172	2610021K21Rik	78767
Chr12	101538979	101759032	NM_001033213	Ttc7b	104718
Chr12	111839604	111905154	NM_030238	Dync1h1	13424
Chr12	111905898	111920829	NM_025488	1700001K19Rik	66323
Chr13	38243162	38290446	NM_023842	Dsp	109620
Chr13	38296807	38319532	NM_026382	Snrnp48	67797
Chr13	42775991	43233881	NM_198419	Phactr1	218194
Chr13	69751121	69774535	NM_145354	Nsun2	28114
Chr13	69841709	69878775	NM_001145162	Ube2ql1	76980
Chr13	94827750	94965364	NM_172589	Lhfpl2	218454
Chr14	20570478	20631009	NM_008695	Nid2	18074
Chr14	20630623	20643045	NM_026528	2700060E02Rik	68045

Chr14	55840843	55860277	NM_027790	Dhrs2	71412
Chr14	65271367	65425133	NM_001081177	Kif13b	16554
Chr14	115491436	116924414	NM_175500	Gpc5	103978
Chr14	121310415	121347266	NM_023579	Ipo5	70572
Chr15	12963954	13021632	BC107008	Cdh6	12563
Chr15	35301300	35860984	NM_177151	Vps13b	666173
Chr15	41279027	41692593	NM_001130166	Oxr1	170719
Chr15	72420049	72886240	BC034590	Trappc9	76510
Chr15	84753830	84773393	BC060234	Nup50	18141
Chr15	84776149	84818401	NM_001033273	5031439G07Rik	223739
Chr15	85166674	85293641	BC046802	Atxn10	54138
Chr17	36179905	36195590	NM_001034909	EG547347	547347
Chr17	36258672	36265600	BC147141	EG547347	547347
Chr17	43407277	43461686	BC019217	Gpr110	77596
Chr17	44914119	45256233	NM_178652	Supt3h	109115
Chr17	47277890	47496402	BC059215	Trerf1	224829
Chr17	47505835	47515627	NM_001146211	Mrps10	64657
Chr17	47522341	47529916	NM_146079	Guca1b	107477
Chr17	47531506	47537500	BC031810	Guca1a	14913
Chr17	53706627	53810932	BC145896	Kat2b	18519
Chr17	53814111	53828640	NM_028232	Sgol1	72415
Chr17	53863075	53865058	NM_001037718	4932415M13Rik	211496
Chr17	80922889	80963174	NM_001033443	Cdkl4	381113
Chr17	80979851	81127433	NM_001081357	Map4k3	225028
Chr17	85104586	85189860	BC059862	Lrpprc	72416
Chr18	52927362	53075584	NM_026408	Sncaip	67847
Chr18	56685389	56721952	NM_138600	Aldh7a1	110695
Chr18	56722222	56747366	NM_001162989	Phax	56698
Chr18	56748001	56754433	NM_026099	1700065I17Rik	67343
Chr18	57514394	57552373	NM_028447	Prrc1	73137
Chr18	73929693	73975046	NM_145494	Me2	107029
Chr18	74019040	74038788	NM_027741	Mro	71263
Chr18	80802943	80904912	NM_001164111	Nfatc1	18018
Chr18	85104416	85150784	NM_015798	Fbxo15	50764
Chr18	85116685	85120916	NM_025969	1700034H14Rik	67105
Chr19	6184409	6224219	NM_001165919	1700123I01Rik	622554
Chr19	6226400	6227767	NM_130453	Gpha2	170458
Chr19	6227764	6235631	BC058977	Ppp2r5b	225849
Chr19	6241667	6262304	NM_194348	Atg2a	329015
Chr19	6276895	6300096	NM_010119	Ehd1	13660
ChrX	3748193	3749684	NM_001099643	Gm3750	100042254
ChrX	4446526	4448017	NM_001099643	Gm3750	100042254
ChrX	31060181	31060959	BC117727	4930408F14Rik	74851
ChrX	31574488	31575266	BC117726	4930408F14Rik	74851
ChrX	50423173	50467568	BC051666	Plac1	56096
ChrX	52683411	52700069	BC171942	4930527E24Rik	75140
ChrX	52804772	52821634	BC171941	4930527E24Rik	75140
ChrX	75983101	75984749	NM_001164739	Fam47a	70864
ChrX	78315982	78343214	NM_138751	Tmem47	192216
ChrX	83067323	83134149	BC115576	5430427O19Rik	71398
ChrX	86997606	87000830	NM_001085511	4932429P05Rik	245509
ChrX	93650774	93769821	NM_010417	Heph	15203
ChrX	112018620	112019550	NR_003645	Ube2dnl	237009
ChrX	112021190	112022119	NM_001081661	4930524E20Rik	75097
ChrX	131071794	131076156	BC004796	Timm8a1	30058
ChrX	131076874	131117603	BC053392	Btk	12229
ChrX	131120192	131122599	NM_019865	Rpl36a	19982
ChrX	133200913	133202916	NM_001029978	Tceal3	594844
ChrX	133242570	133244412	BC083157	Tceal1	237052
ChrX	153985753	154036647	NM_172307	Mbtps2	270669

ChrY	234230	280254	NM_011419	Kdm5d	20592

Supplemenary Table 2. List of 1272 genes that are upregulated in LIF- compared to NPCs

Systematic	Transcript ID_Affymetrix	Genbank ID	Gene symbol	NPCs	LIF+	LIF-	LIF+ /NPCs	LIF- /NPCs
1456046_at	Mm.69004.1	AV319144	C1qr1	0.067	0.117	4.857	1.731	71.990
1419589_at	Mm.681.1	BB039247	C1qr1	0.095	0.059	4.936	0.621	52.096
1435500_at	Mm.22998.1	BB270778	Rab26	0.030	0.009	1.257	0.299	42.192
1448998_at	Mm.41236.1	NM_080420	Lpo	0.083	0.200	3.436	2.415	41.549
1419152_at	Mm.45765.1	AK017673	2810417H13Rik	0.211	0.525	5.364	2.488	25.397
1419292_at	Mm.41957.1	NM_030127	9530081K03Rik	0.075	0.852	1.849	11.326	24.585
1422583_at	Mm.41580.1	NM_023537	Rab3b	1.185	1.991	28.946	1.680	24.427
1416200_at	Mm.182359.1	NM_133775	9230117N10Rik	0.071	0.756	1.387	10.685	19.607
1424528_at	Mm.45127.1	BC023116	Cgref1	0.068	0.797	1.293	11.797	19.146
1418744_s_at	Mm.26378.1	NM_021344	Tesc	0.119	0.388	1.441	3.251	12.063
1424936_a_at	Mm.108242.1	AF356522	Dnahc8	0.107	0.020	1.132	0.189	10.553
1451304_at	Mm.34754.1	BC017618	2310076O21Rik	0.100	0.466	1.057	4.636	10.517
1434191_at	Mm.36638.1	AI790538	A530016O06Rik	0.134	0.538	1.377	4.015	10.266
1457664_x_at	Mm.56884.1	AV227574	C2	0.123	0.076	1.093	0.616	8.898
1430588_at	Mm.126220.1	BI076815	Mro	0.177	0.982	1.565	5.542	8.830
1435126_at	Mm.39225.1	BB174864	Dusp15	0.253	0.589	2.193	2.326	8.662
1418186_at	Mm.2746.1	BC012254	Gstt1	0.882	0.826	7.443	0.936	8.436
1447474_at	Mm.214498.1	BB799448	Ramp1	0.218	0.593	1.842	2.714	8.433
1417419_at	Mm.22288.1	NM_007631	Ccnd1	3.627	4.156	30.571	1.146	8.428
1417065_at	Mm.181959.1	NM_007913	Egr1	1.849	1.233	15.138	0.667	8.187
1418472_at	Mm.20317.1	BC024934	Aspa	0.354	0.876	2.707	2.476	7.647
1419502_at	Mm.37308.1	NM_031871	D11Lgp1e	0.144	0.792	1.032	5.487	7.145
1434877_at	Mm.5142.2	AI152800	Nptx1	0.676	0.286	4.798	0.424	7.095
1427284_a_at	Mm.24375.1	AU019171	Ttpa	0.763	1.062	5.395	1.392	7.074
1435615_at	Mm.39548.2	BB277790	Zfp365	0.270	0.816	1.884	3.017	6.964
1426872_at	Mm.205520.1	BC026653	A430096B05Rik	0.190	0.819	1.317	4.299	6.914
1428611_at	Mm.34548.1	AW549826	Apg7l	0.150	0.878	1.019	5.856	6.796
1453173_at	Mm.70371.1	AK019906	2310005E10Rik	2.012	1.907	13.552	0.948	6.737
1423062_at	Mm.29254.1	AV175389	Igfbp3	3.012	5.298	20.057	1.759	6.658
1448600_s_at	Mm.103805.1	BC027242	Vav3	1.529	2.020	10.096	1.321	6.602
1417122_at	Mm.103805.1	BC027242	Vav3	1.310	1.812	8.572	1.384	6.545
1454015_a_at	Mm.24700.2	AK016527	Cdh13	0.705	1.186	4.494	1.682	6.375
1432091_a_at	Mm.29957.2	AK007455	Rutbc3	0.194	0.801	1.225	4.131	6.320
1423420_at	Mm.46797.1	AK018378	Adrb1	0.266	0.835	1.676	3.139	6.299
1431168_at	Mm.150838.1	AW209946	E130112H22Rik	0.243	0.384	1.497	1.582	6.162
1448698_at	Mm.22288.1	NM_007631	Ccnd1	7.956	10.158	48.859	1.277	6.141
1415834_at	Mm.1791.1	NM_026268	Dusp6	4.153	0.945	24.976	0.228	6.014
1448752_at	Mm.1186.1	NM_009801	Car2	6.701	9.482	39.963	1.415	5.963
1415803_at	Mm.3205.1	AF010586	Cx3cl1	1.789	2.060	10.586	1.151	5.918
1456341_a_at	Mm.19788.5	AV354744	Bteb1	0.703	1.005	4.065	1.431	5.786
1422428_at	Mm.20592.1	NM_053178	Lpd	4.416	4.459	25.210	1.010	5.709
1435743_at	Mm.138073.1	AW548377	C130068N17Rik	2.587	4.275	14.724	1.652	5.691
1423756_s_at	Mm.22248.2	BC019836	Igfbp4	2.083	0.422	11.689	0.202	5.612
1448676_at	Mm.4857.1	NM_007595	Camk2b	1.049	1.097	5.807	1.046	5.538
1428434_at	Mm.41601.1	AK012833	2810028A01Rik	1.743	2.117	9.361	1.215	5.370
1428303_at	Mm.38347.1	AK005160	1500005I02Rik	0.694	1.051	3.648	1.513	5.255
1457536_at	Mm.186305.1	BB171986	Gpc5	0.191	0.086	1.001	0.451	5.227
1448831_at	Mm.3425.1	NM_007426	Agpt2	0.291	0.384	1.517	1.318	5.211
1424248_at	Mm.32794.1	BB159263	0710001E13Rik	0.977	0.441	5.077	0.452	5.198

1426432_a_at	Mm.34747.2	BE655147	Slc4a4	2.177	2.895	11.283	1.330	5.184
1437405_a_at	Mm.22248.4	BB787243	Igfbp4	3.514	0.767	17.964	0.218	5.112
1434736_at	Mm.45146.2	BB744589	Hlf	0.613	0.358	3.124	0.584	5.092
1434881_s_at	Mm.24647.1	BM220945	Kctd12	0.845	0.678	4.282	0.802	5.066
1423222_at	Mm.44529.1	AV261931	Cap2	0.723	1.249	3.628	1.728	5.020
1458268_s_at	Mm.200652.1	AI649005	Igfbp3	3.569	5.432	17.902	1.522	5.016
1416592_at	Mm.29728.1	AF276917	Glrx1	2.398	3.524	11.803	1.469	4.922
1416749_at	Mm.30156.1	NM_019564	Prss11	2.654	2.559	13.013	0.964	4.903
1438251_x_at	Mm.30156.2	BB559067	Prss11	1.552	1.315	7.573	0.848	4.881
1435467_at	Mm.23273.1	BM936783	Fgd6	0.216	0.925	1.040	4.289	4.825
1428393_at	Mm.40663.1	AK003046	Nrn1	0.806	1.288	3.885	1.598	4.820
1417420_at	Mm.22288.1	NM_007631	Ccnd1	6.266	5.363	30.130	0.856	4.809
1436876_at	Mm.100348.1	AV337421	D13Bwg1146e	0.928	0.882	4.443	0.951	4.788
1439940_at	Mm.44658.1	AV328280	Slc1a2	0.728	0.937	3.470	1.288	4.769
1418754_at	Mm.1425.1	NM_009623	Adcy8	0.306	0.492	1.446	1.610	4.733
1426714_at	Mm.25013.1	AK003278	D11Ertd18e	0.244	0.729	1.152	2.993	4.726
1428574_a_at	Mm.34715.2	AK006398	Chn2	0.737	1.213	3.474	1.646	4.714
1423551_at	Mm.24700.1	BB776961	Cdh13	3.239	4.581	15.164	1.414	4.682
1454070_a_at	Mm.24760.2	AK014975	4921528E07Rik	1.135	1.859	5.169	1.638	4.553
1429909_at	Mm.63526.1	AK014682	4833411O04Rik	0.261	0.758	1.156	2.908	4.436
1425416_s_at	Mm.196677.1	BC008994	5430413I02Rik	1.778	0.996	7.748	0.561	4.358
1415804_at	Mm.3205.1	AF010586	Cx3cl1	0.763	0.997	3.323	1.306	4.354
1435790_at	Mm.32720.1	BG864960	A030009A06	1.726	3.178	7.411	1.841	4.293
1418108_at	Mm.28352.1	NM_133244	Plekhk1	0.472	0.297	2.015	0.628	4.268
1433583_at	Mm.39548.1	AV327248	Zfp365	1.614	2.562	6.746	1.588	4.181
1423640_at	Mm.41729.1	BC026512	Synpr	0.682	0.706	2.821	1.034	4.134
1416431_at	Mm.181860.1	NM_026473	2310057H16Rik	1.545	1.373	6.358	0.889	4.115
1416593_at	Mm.29728.1	AF276917	Glrx1	2.313	2.763	9.494	1.195	4.104
1422484_at	Mm.35389.1	NM_007808	Cycs	7.473	12.305	30.486	1.647	4.080
1455963_at	Mm.25840.2	AV317707	6332401O19Rik	3.620	2.538	14.616	0.701	4.038
1451280_at	Mm.32794.1	BB159263	0710001E13Rik	0.356	0.138	1.436	0.389	4.038
1418478_at	Mm.12607.1	NM_057173	Lmo1	2.397	2.150	9.643	0.897	4.024
1450506_a_at	Mm.34109.1	NM_026531	2700083B06Rik	1.129	2.206	4.524	1.955	4.009
1450910_at	Mm.44529.1	AV261931	Cap2	1.096	2.181	4.371	1.990	3.988
1435695_a_at	Mm.27337.2	AA673177	A030007L17Rik	0.517	0.870	2.038	1.683	3.942
1416541_at	Mm.3990.1	NM_009191	Skd3	1.330	2.220	5.232	1.669	3.933
1452961_at	Mm.41187.2	AK004681	1200009O22Rik	3.916	4.708	15.355	1.202	3.921
1420957_at	Mm.7883.1	NM_007462	Apc	1.948	3.184	7.592	1.634	3.897
1426147_s_at	Mm.104972.1	BC021770	Cldn10	0.551	0.123	2.143	0.223	3.889
1453752_at	Mm.5290.2	BF453369	Rpl17	1.546	2.355	5.990	1.523	3.874
1441927_at	Mm.45546.1	AV138438	Syt7	0.777	0.741	3.010	0.953	3.872
1428573_at	Mm.34715.2	AK006398	Chn2	1.720	2.535	6.640	1.474	3.861
1449638_at	Mm.25031.1	W41129	C920021L13Rik	0.290	0.744	1.111	2.571	3.837
1434096_at	Mm.41044.1	BB283443	Slc4a4	5.940	7.842	22.373	1.320	3.767
1438671_at	Mm.41694.1	AV223153	6330548O06Rik	0.394	0.656	1.483	1.666	3.764
1450100_a_at	Mm.67998.1	AW046403	Tcerg1	0.317	0.665	1.191	2.099	3.759
1431812_a_at	Mm.2834.2	AK014572	Slc6a9	1.249	0.712	4.681	0.571	3.749
1429321_at	Mm.28614.1	T12280	Rnf149	2.418	4.542	9.053	1.878	3.744
1437406_x_at	Mm.22248.4	BB787243	Igfbp4	1.940	0.525	7.260	0.270	3.742
1424966_at	Mm.29739.1	BC019416	9030407H20Rik	0.927	1.092	3.460	1.178	3.734
1440085_at	Mm.189270.1	AV246296	Xedar	1.344	1.483	5.010	1.103	3.727
1416029_at	Mm.4292.1	NM_013692	Tieg1	1.911	1.388	7.082	0.726	3.706

1423405_at	Mm.36851.1	BI788452	Timp4	4.447	0.254	16.483	0.057	3.706
1436849_x_at	Mm.4793.3	BB357227	Gaa	1.863	3.542	6.891	1.902	3.699
1416051_at	Mm.2081.1	NM_013484	C2	0.358	0.099	1.323	0.277	3.693
1425349_a_at	Mm.18535.1	U13262	Myef2	1.063	1.812	3.918	1.705	3.686
1417930_at	Mm.6549.1	NM_008668	Nab2	1.431	1.151	5.256	0.804	3.673
1455321_at	Mm.206763.1	BM226111	4921528E07Rik	0.961	1.460	3.524	1.518	3.666
1423077_at	Mm.89515.1	BB703513	Snx9	1.150	2.029	4.211	1.765	3.663
1456319_at	Mm.196322.1	BG065719	X83313	4.452	5.043	16.207	1.133	3.640
1460449_at	Mm.45416.1	BQ174247	C030032C09Rik	0.675	0.596	2.442	0.884	3.621
1436934_s_at	Mm.215115.2	AU019938	Aco2	11.898	18.102	42.980	1.521	3.612
1435069_at	Mm.34689.1	AW493518	BC064078	1.140	1.470	4.114	1.289	3.609
1451318_a_at	Mm.1834.1	M57697	Lyn	1.940	1.054	6.998	0.543	3.608
1448340_at	Mm.171292.1	BE986812	2010200I23Rik	2.766	2.925	9.973	1.057	3.605
1417212_at	Mm.34532.1	NM_026633	9530058B02Rik	1.369	1.165	4.931	0.851	3.601
1417168_a_at	Mm.12914.1	AI553394	Usp2	1.124	2.132	4.043	1.897	3.596
1426831_at	Mm.27733.1	BB831090	Ahcyl1	5.371	9.555	19.242	1.779	3.583
1435135_at	Mm.24576.1	AV369935	B230106I24Rik	0.372	0.952	1.334	2.557	3.582
1417300_at	Mm.17794.1	NM_133888	Smpdl3b	0.783	1.239	2.794	1.583	3.570
1448690_at	Mm.10800.1	NM_008430	Kcnk1	0.586	1.087	2.084	1.854	3.555
1434735_at	Mm.45146.2	BB744589	Hlf	1.140	0.418	4.030	0.367	3.535
1456812_at	Mm.40168.1	AW456685	Abcd2	0.341	0.128	1.198	0.375	3.514
1449402_at	Mm.44827.1	AB046929	Chst7	3.969	2.773	13.932	0.699	3.510
1450437_a_at	Mm.4974.1	NM_010875	Ncam1	2.062	2.293	7.173	1.112	3.478
1427441_a_at	Mm.34632.1	BF608645	Suclg2	2.244	3.217	7.775	1.434	3.465
1459048_s_at	Mm.132783.1	BB154236	Zfp142	0.331	0.569	1.144	1.718	3.454
1422493_at	Mm.35820.1	BG067254	Cpox	0.420	1.000	1.449	2.382	3.453
1444062_at	Mm.23499.1	AI428930	2900056L01Rik	0.688	0.699	2.373	1.016	3.451
1435438_at	Mm.131065.1	AV345303	Sox8	3.400	4.153	11.657	1.222	3.429
1434115_at	Mm.27614.1	BQ176681	Cdh13	3.663	4.537	12.501	1.238	3.412
1423594_a_at	Mm.2377.1	BB451714	Ednrb	10.523	11.742	35.782	1.116	3.400
1439633_at	Mm.36490.1	BQ175636	B230112P13Rik	0.405	0.649	1.376	1.603	3.397
1454168_a_at	Mm.3990.2	AK006072	Skd3	0.405	0.525	1.359	1.297	3.356
1435733_x_at	Mm.28690.2	AI646809	1500026D16Rik	1.885	2.763	6.293	1.465	3.338
1431413_at	Mm.159971.1	BI665680	9130218E19Rik	0.370	0.748	1.232	2.020	3.327
1456944_at	Mm.39204.1	BB529153	C230009H10Rik	3.284	1.419	10.918	0.432	3.325
1435653_at	Mm.161353.1	BG070904	Abhd2	5.269	10.174	17.469	1.931	3.315
1417619_at	Mm.70462.1	BE368753	Gadd45gip1	2.140	3.602	7.046	1.683	3.292
1453257_at	Mm.24117.1	BB758649	D8Ertd319e	3.262	5.263	10.660	1.613	3.267
1433858_at	Mm.31247.2	BB667092	1300004K21Rik	2.047	2.520	6.678	1.231	3.263
1449893_a_at	Mm.944.1	NM_008377	Lrig1	6.584	10.766	21.429	1.635	3.255
1431274_a_at	Mm.2849.2	AA543265	Hspa9a	0.639	0.974	2.063	1.524	3.227
1435417_at	Mm.27054.1	BG063189	AI464131	2.014	2.176	6.496	1.081	3.226
1426264_at	Mm.38786.1	AV336908	Dlat	2.925	4.793	9.412	1.639	3.217
1431352_s_at	Mm.202176.1	BI453402	Pvt1	0.393	0.502	1.264	1.276	3.215
1416108_a_at	Mm.27606.1	NM_025360	1200002G13Rik	3.888	7.725	12.496	1.987	3.214
1456600_a_at	Mm.28235.3	AV047821	Rnf7	5.307	10.390	17.009	1.958	3.205
1450295_s_at	Mm.8071.1	NM_009310	D7Ertd458e	0.315	0.769	1.011	2.437	3.204
1417273_at	Mm.10283.1	NM_013743	Pdk4	0.463	0.945	1.483	2.039	3.202
1422135_at	Mm.42054.1	NM_011980	Zfp146	0.790	1.200	2.528	1.519	3.199
1417916_a_at	Mm.4050.1	NM_019502	Fxc1	8.159	14.970	26.040	1.835	3.192
1438207_at	Mm.44823.1	BB744704	AW049900	0.434	0.891	1.384	2.052	3.188
1433733_a_at	Mm.26237.2	BG069864	Cry1	0.936	1.324	2.982	1.415	3.186

1424313_a_at	Mm.28712.1	BC013503	Ndufs7	9.932	17.057	31.599	1.717	3.181
1422126_a_at	Mm.41966.1	NM_026341	Nudt13	0.543	0.924	1.725	1.703	3.180
1418504_at	Mm.2849.1	D11089	Hspa9a	8.297	15.741	26.363	1.897	3.177
1444422_at	Mm.210225.1	BB732600	B530002L05Rik	0.375	0.761	1.186	2.031	3.165
1452142_at	Mm.5260.1	M92378	Slc6a1	14.195	13.669	44.916	0.963	3.164
1449943_at	Mm.12834.1	NM_008494	Lfng	0.785	0.973	2.477	1.240	3.155
1426314_at	Mm.2377.2	BB770914	Ednrb	10.132	11.895	31.950	1.174	3.153
1436416_x_at	Mm.4050.4	BB609429	Fxc1	4.729	7.712	14.836	1.631	3.137
1447919_x_at	Mm.176233.1	AV092283	Ndufab1	8.875	14.074	27.802	1.586	3.133
1455272_at	Mm.128925.1	BB429139	Grm5	3.758	4.717	11.770	1.255	3.132
1433771_at	Mm.28796.2	AV173092	5730446C15Rik	4.229	7.705	13.211	1.822	3.124
AFFX-TransRecMur/X57349_3_at	AFFX-TransRecMur/X57349_3	AFFX-TransRecMur/X57349_3	Tfrc	0.586	0.715	1.826	1.221	3.116
1448887_x_at	Mm.4050.1	NM_019502	Fxc1	4.362	7.216	13.572	1.654	3.111
1427918_a_at	Mm.826.1	BI081723	Rhoq	12.194	10.498	37.874	0.861	3.106
1453468_at	Mm.3447.2	AV312369	4930430F08Rik	0.779	1.096	2.419	1.407	3.104
1423350_at	Mm.37204.1	AA510713	Socs5	0.704	0.973	2.184	1.381	3.102
1424828_a_at	Mm.41502.1	BC006048	Fh1	13.135	19.689	40.713	1.499	3.099
1426189_at	Mm.219646.1	AF357887	Dusp15	0.413	0.350	1.280	0.847	3.096
1418365_at	Mm.2277.1	NM_007801	Ctsh	0.606	1.170	1.871	1.929	3.086
1418665_at	Mm.34079.1	NM_053261	Impa2	0.587	0.194	1.810	0.330	3.086
1439894_at	Mm.40846.1	BB253214	A730056I06Rik	2.962	1.390	9.105	0.469	3.074
1429453_a_at	Mm.34801.2	BM209870	Mrpl55	2.474	4.836	7.575	1.955	3.062
1443823_s_at	Mm.181435.1	AV325919	Atp1a2	15.820	3.887	48.417	0.246	3.060
1452048_at	Mm.133851.1	AK002757	Mrpl12	7.136	8.990	21.782	1.260	3.052
1417430_at	Mm.1640.1	NM_007672	Cdr2	0.416	0.353	1.267	0.849	3.045
1421915_a_at	Mm.41978.1	AK005053	Siat6	0.342	0.542	1.037	1.585	3.030
1426917_s_at	Mm.220950.1	BM211317	4833415E20Rik	0.505	0.879	1.527	1.741	3.026
1434595_at	Mm.76764.1	BQ174474	Trim9	2.562	2.868	7.750	1.120	3.025
1417323_at	Mm.23776.1	NM_019976	5430413I02Rik	4.824	3.240	14.586	0.672	3.024
1454886_x_at	Mm.40472.1	BB083438	Trim9	5.121	9.888	15.446	1.931	3.016
1435275_at	Mm.29625.1	AV013496	Coxvib2	3.697	4.940	11.140	1.336	3.013
1435605_at	Mm.40406.1	BB125424	Arp3b	1.036	0.498	3.118	0.481	3.009
1438832_x_at	Mm.30243.6	BB461484	Dhx30	2.032	2.702	6.107	1.330	3.006
1424356_a_at	Mm.24349.1	BC024445	BC019776	1.309	1.637	3.933	1.251	3.005
1426622_a_at	Mm.46475.1	BB150720	Qpct	1.029	0.914	3.085	0.888	2.998
1432016_a_at	Mm.196043.2	AK003393	Idh3a	12.938	17.021	38.767	1.316	2.996
1436506_a_at	Mm.28311.1	AW988981	1110008H02Rik	10.897	17.799	32.625	1.633	2.994
1422558_at	Mm.7329.1	AF015887	Gamt	2.562	3.315	7.633	1.294	2.979
1448919_at	Mm.30109.1	NM_025422	1110055L24Rik	1.411	1.983	4.199	1.405	2.976
1425139_at	Mm.23608.1	AV308638	Sesn2	1.016	1.769	3.020	1.742	2.974
1451096_at	Mm.21669.1	BC016097	Ndufs2	15.014	25.942	44.603	1.728	2.971
1460656_a_at	Mm.21104.1	NM_134114	5630401J11Rik	4.650	6.948	13.811	1.494	2.970
1431340_a_at	Mm.196252.2	AK010048	2310002J21Rik	0.560	0.878	1.661	1.569	2.968
1417636_at	Mm.2834.1	NM_008135	Slc6a9	4.413	2.417	13.083	0.548	2.964
1424671_at	Mm.30886.1	BC002120	Plekhf1	1.677	1.899	4.956	1.132	2.955
1425143_a_at	Mm.218595.1	BC006660	Ndufs1	8.333	11.463	24.469	1.376	2.936
1420477_at	Mm.3797.1	BG064031	Nap1l1	6.089	7.723	17.864	1.268	2.934
1456395_at	Mm.10707.3	BM120569	A830037N07Rik	1.065	1.330	3.119	1.248	2.928
1428782_a_at	Mm.972.3	AK004031	Uqcrc1	18.927	36.593	55.335	1.933	2.924
1424184_at	Mm.18630.1	BC026559	Acadvl	7.277	13.330	21.258	1.832	2.921
1426554_a_at	Mm.16783.3	BI407347	Pgam1	16.266	31.935	47.511	1.963	2.921
1456653_a_at	Mm.184752.3	AV095209	Fthfsdc1	0.655	0.414	1.912	0.632	2.919

1428507_at	Mm.28525.1	AK014208	3110052N05Rik	4.002	6.912	11.673	1.727	2.917
1454159_a_at	Mm.141936.2	AK011784	Igfbp2	33.062	21.415	96.250	0.648	2.911
1439374_x_at	Mm.39130.6	AV110855	Rps10	20.646	18.115	60.009	0.877	2.907
1437454_a_at	Mm.27328.2	AV103255	2310042M24Rik	7.519	9.624	21.844	1.280	2.905
1448562_at	Mm.4610.1	NM_009477	Upp1	1.702	0.803	4.937	0.471	2.900
1427037_at	Mm.219670.1	BF227830	Eif4g1	1.516	2.785	4.393	1.837	2.897
1440248_at	Mm.170955.1	BB238478	D130060C09Rik	0.987	1.765	2.855	1.788	2.892
1416662_at	Mm.141020.1	BI217574	Sardh	1.310	2.040	3.785	1.558	2.890
1457743_at	Mm.36396.1	BQ175190	AI842293	0.720	0.317	2.078	0.440	2.887
1433702_at	Mm.27216.1	BI663634	D19Wsu12e	4.486	4.581	12.948	1.021	2.886
1435446_a_at	Mm.21754.3	BF180212	Chpt1	1.911	3.624	5.514	1.896	2.885
1455586_at	Mm.217250.1	BB275228	3110001H15Rik	0.458	0.628	1.322	1.370	2.884
1447837_x_at	Mm.50720.1	BB066090	Polh	1.194	0.964	3.439	0.808	2.881
1452919_a_at	Mm.28541.1	W59405	1700012G19Rik	4.934	4.936	14.215	1.000	2.881
1417147_at	Mm.5166.1	NM_019833	B230317C12Rik	1.963	2.478	5.652	1.262	2.879
1426146_a_at	Mm.21754.1	BC016251	Chpt1	1.083	1.708	3.116	1.577	2.879
1429005_at	Mm.5601.1	BB107412	Mfhas1	5.781	9.618	16.638	1.664	2.878
1433679_at	Mm.45384.1	BB399719	A330051M14Rik	3.258	5.133	9.368	1.576	2.876
1434249_s_at	Mm.40472.1	BB083438	Trim9	2.158	3.894	6.191	1.805	2.870
1425576_at	Mm.220328.1	BC018218	Ahcyl1	3.679	5.427	10.557	1.475	2.870
1456119_at	Mm.101058.1	AV334671	Grm5	2.149	1.914	6.155	0.891	2.865
1437559_at	Mm.100348.2	BB130891	D13Bwg1146e	1.077	0.582	3.081	0.541	2.861
1420545_a_at	Mm.41190.1	NM_029716	Chn1	1.134	1.408	3.242	1.242	2.859
1427166_a_at	Mm.29620.1	BM212545	Spg7	1.478	2.405	4.221	1.627	2.856
1452713_a_at	Mm.29606.1	AK002371	0610009C03Rik	2.634	3.450	7.501	1.310	2.847
1426265_x_at	Mm.38786.1	AV336908	Dlat	3.417	5.002	9.721	1.464	2.845
1424475_at	Mm.87620.1	BI157430	Camkk2	0.361	0.860	1.027	2.381	2.842
1460408_at	Mm.147354.1	AF156490	Gabrg1	3.076	5.517	8.717	1.794	2.834
1456643_at	Mm.75160.1	BB043558	9230114K14Rik	0.440	0.476	1.244	1.082	2.827
1448339_at	Mm.171292.1	BE986812	2010200I23Rik	9.281	17.114	26.225	1.844	2.826
1422483_a_at	Mm.35389.1	NM_007808	Cycs	18.878	25.172	53.293	1.333	2.823
1419103_a_at	Mm.181473.1	NM_025341	Abhd6	1.451	1.942	4.092	1.338	2.819
1417663_a_at	Mm.36775.1	BE631549	Ndrg3	2.879	5.711	8.116	1.984	2.819
1427965_at	Mm.131705.1	BG073014	Ssbp1	7.589	14.768	21.349	1.946	2.813
1447703_x_at	Mm.177940.1	AV214133	3110024A21Rik	0.773	1.507	2.175	1.949	2.813
1458534_at	Mm.44348.1	BB281929	D13Bwg1146e	0.900	0.322	2.530	0.357	2.812
1429533_at	Mm.183073.1	BB222675	Immt	2.969	3.944	8.342	1.328	2.809
1419748_at	Mm.4817.1	NM_011994	Abcd2	0.445	0.183	1.249	0.410	2.808
1417624_at	Mm.25903.1	NM_008667	Nab1	2.807	5.484	7.870	1.954	2.804
1450537_at	Mm.25678.1	AF196480	Mid2	0.359	0.936	1.007	2.605	2.803
1435642_at	Mm.40741.1	BB081437	9630019K15Rik	3.519	1.801	9.857	0.512	2.801
1451158_at	Mm.173718.1	BG923744	Gtl6	1.051	1.892	2.944	1.799	2.800
1448540_a_at	Mm.41683.1	BC021536	0610012G03Rik	3.150	4.207	8.800	1.336	2.794
1450387_s_at	Mm.42040.1	NM_009647	Ak4	1.787	1.558	4.981	0.872	2.788
1427938_at	Mm.220922.1	BB046347	Mycbp	0.391	0.922	1.089	2.359	2.788
1417192_at	Mm.180299.1	NM_138599	D16Wsu109e	1.738	2.706	4.845	1.557	2.787
1429187_at	Mm.220909.1	AV306753	3930401E15Rik	3.882	7.487	10.817	1.928	2.786
1436343_at	Mm.183257.3	BM502696	Chd4	1.573	3.049	4.381	1.939	2.786
1448273_at	Mm.7504.1	NM_008180	Gss	0.795	1.506	2.213	1.895	2.785
1448987_at	Mm.2445.1	BB728073	Acadl	19.969	31.940	55.547	1.600	2.782
1440901_at	Mm.101340.1	BB279524	Dgkb	0.501	0.641	1.394	1.278	2.780
1436746_at	Mm.27341.1	BI692255	Prkwnk1	6.732	12.522	18.715	1.860	2.780

1456541_x_at	Mm.21881.4	BB357220	Atad3a	1.881	2.055	5.225	1.093	2.778
1427981_a_at	Mm.41853.1	AY033912	Csad	1.822	3.218	5.051	1.767	2.773
1452893_s_at	Mm.34074.1	BB772205	2310040A07Rik	4.271	5.185	11.824	1.214	2.769
1433970_at	Mm.23856.1	BB610351	1810056O20Rik	4.264	7.386	11.795	1.732	2.766
1419548_at	Mm.6952.1	U20619	Kpna1	2.058	3.508	5.688	1.705	2.764
1448619_at	Mm.182149.1	NM_007856	Dhcr7	6.244	9.104	17.255	1.458	2.763
1450731_s_at	Mm.200792.1	BG972377	Tnfrsf21	4.987	8.675	13.776	1.739	2.762
1421225_a_at	Mm.34747.1	NM_018760	Slc4a4	0.616	0.320	1.699	0.520	2.759
1424831_at	Mm.23333.1	BC023348	Cpne2	1.403	2.366	3.869	1.687	2.759
1421260_a_at	Mm.10.1	NM_009272	Srm	6.318	8.084	17.428	1.280	2.758
1451272_a_at	Mm.44214.2	BC016117	2510010F15Rik	3.200	6.036	8.826	1.886	2.758
1420967_at	Mm.200907.1	AW541300	Slc25a15	2.085	3.357	5.744	1.610	2.755
1453485_s_at	Mm.200744.1	AK016897	1110005A03Rik	1.204	2.086	3.317	1.732	2.755
1424684_at	Mm.29829.1	BC023027	Rab5c	5.856	10.233	16.129	1.747	2.754
1452130_at	Mm.27328.1	BI790903	2310042M24Rik	8.236	15.887	22.683	1.929	2.754
1416661_at	Mm.2238.1	AW701127	Eif3s10	0.775	1.329	2.130	1.714	2.749
1451084_at	Mm.28336.1	BC012522	Etfdh	5.090	9.385	13.982	1.844	2.747
1433897_at	Mm.41720.1	BQ176744	AI597468	5.703	9.813	15.666	1.721	2.747
1459784_x_at	Mm.136169.1	BB521298	1300002A08Rik	0.436	0.853	1.196	1.956	2.742
1433562_s_at	Mm.148721.2	AA097406	Atp5f1	26.829	53.467	73.538	1.993	2.741
1460706_s_at	Mm.195755.1	AV013771	1110060F11Rik	1.112	1.850	3.046	1.663	2.738
1449523_at	Mm.2898.1	NM_009746	Bcl7c	2.391	3.313	6.543	1.386	2.736
1416114_at	Mm.29027.1	NM_010097	Sparcl1	12.488	21.815	34.126	1.747	2.733
1431032_at	Mm.17221.1	AA681807	Agl	2.041	3.259	5.571	1.597	2.730
1423648_at	Mm.182959.1	BC006865	Txndc7	7.667	12.843	20.914	1.675	2.728
1415966_a_at	Mm.29842.1	NM_133666	Ndufv1	9.326	14.145	25.438	1.517	2.728
1424345_s_at	Mm.196580.1	BC021792	Ube2m	4.396	7.874	11.990	1.791	2.727
1452071_at	Mm.34747.2	BE655147	Slc4a4	4.173	3.458	11.376	0.829	2.726
1449628_s_at	Mm.219882.1	AI852671	Stard7	11.975	21.690	32.638	1.811	2.726
1456464_x_at	Mm.181623.1	BB129990	Syt11	17.048	32.895	46.430	1.930	2.723
1438317_a_at	Mm.4449.3	AV104666	Endog	1.020	1.192	2.776	1.168	2.721
1427465_at	Mm.193539.3	BC025807	Atp1a2	4.319	1.119	11.741	0.259	2.719
1448005_at	Mm.24385.1	BI658899	2500002E12Rik	8.257	15.428	22.432	1.868	2.717
1448558_a_at	Mm.4186.1	NM_008869	Pla2g4a	1.130	2.145	3.070	1.897	2.715
1438431_at	Mm.104643.2	BB197269	Abcd2	0.436	0.128	1.184	0.293	2.715
1435870_at	Mm.148209.2	BM899344	Sycp3	1.788	2.650	4.848	1.482	2.711
1429861_at	Mm.52439.1	BQ177394	Pcdh9	1.393	2.344	3.768	1.682	2.704
1449634_a_at	Mm.156528.2	AW046296	C030032C09Rik	0.735	0.477	1.986	0.649	2.701
1430326_s_at	Mm.196343.1	AK003160	1500040F11Rik	18.971	32.734	51.228	1.725	2.700
1438178_x_at	Mm.21881.3	BB368056	Atad3a	7.456	9.904	20.123	1.328	2.699
1435526_at	Mm.220916.3	BM248600	A130072J07	2.607	4.050	7.034	1.554	2.699
1415821_at	Mm.15125.1	NM_009145	Sdfr1	8.027	14.547	21.637	1.812	2.695
1421097_at	Mm.4449.1	NM_007931	Endog	0.932	1.251	2.510	1.343	2.694
1449936_at	Mm.34182.1	NM_028982	8430419L09Rik	1.095	1.280	2.945	1.169	2.690
1416863_at	Mm.22637.1	NM_022419	Abhd8	2.205	3.525	5.927	1.599	2.688
1425598_a_at	Mm.1834.2	M64608	Lyn	1.381	0.713	3.710	0.516	2.687
1456625_at	Mm.33970.2	BB752547	Aasdhppt	0.513	0.837	1.376	1.633	2.684
1434136_at	Mm.25840.1	BE571820	6332401O19Rik	2.208	0.964	5.925	0.437	2.683
1433839_at	Mm.40274.1	BB431372	AI875199	1.306	1.693	3.500	1.296	2.680
1458440_at	Mm.37803.1	BB827631	B230396K10Rik	3.227	1.979	8.633	0.613	2.675
1416478_a_at	Mm.21743.1	NM_008617	Mdh2	30.386	55.133	81.273	1.814	2.675
1418996_a_at	Mm.46847.1	BC021522	4930469P12Rik	5.600	9.203	14.976	1.643	2.674

1422767_at	Mm.27291.1	BG079188	Bysl	1.916	2.400	5.119	1.253	2.672
1434419_s_at	Mm.219677.2	AW538183	Tardbp	2.329	4.063	6.222	1.745	2.672
1419505_a_at	Mm.36520.1	NM_010282	Ggps1	0.587	1.146	1.567	1.953	2.672
1421847_at	Mm.28489.1	BM730566	Wsb2	7.503	13.991	20.038	1.865	2.671
1433855_at	Mm.1348.2	BF462185	Abat	5.680	10.224	15.161	1.800	2.669
1449256_a_at	Mm.1387.1	BC010722	Rab11a	10.395	20.645	27.748	1.986	2.669
1452173_at	Mm.200497.1	AW107842	Hadha	10.686	18.320	28.521	1.714	2.669
1424075_at	Mm.21640.1	BC024945	9430016H08Rik	3.401	4.380	9.074	1.288	2.668
1449129_a_at	Mm.29653.1	AF300870	Csen	2.678	1.431	7.145	0.534	2.668
1427708_a_at	Mm.1190.4	L22989	Nf2	0.395	0.738	1.053	1.869	2.667
1423927_at	Mm.21470.1	BC025875	Slc35b1	4.337	5.958	11.565	1.374	2.666
1417609_at	Mm.27541.1	BG868960	Ube2a	3.504	6.639	9.333	1.895	2.664
1430295_at	Mm.202311.2	BG094302	Gna13	2.094	3.212	5.577	1.534	2.663
1429382_at	Mm.3077.3	AV253376	Nr1i3	2.316	4.300	6.162	1.857	2.661
1434053_x_at	Mm.19370.2	AV216686	Atp5k	13.825	21.757	36.768	1.574	2.659
1460336_at	Mm.10707.1	BB745167	Ppargc1a	1.262	1.329	3.351	1.053	2.654
1432158_a_at	Mm.34586.2	AK007951	Trappc2	6.999	13.189	18.561	1.884	2.652
1438984_x_at	Mm.368.3	AV212146	Psmb4	13.849	27.627	36.726	1.995	2.652
1436834_x_at	Mm.3156.3	AV011848	Mdh1	14.914	24.355	39.501	1.633	2.649
1447780_x_at	Mm.176854.1	AV097965	2300002G02Rik	1.838	2.630	4.867	1.431	2.648
1427937_at	Mm.41102.1	BB410125	2610030H06Rik	1.787	3.432	4.729	1.921	2.646
1460191_at	Mm.24353.1	NM_019661	0610042I15Rik	4.029	6.761	10.655	1.678	2.645
1448727_at	Mm.206764.1	NM_053254	Tle6	0.915	0.246	2.421	0.269	2.645
1416093_a_at	Mm.23825.1	NM_025570	Mrpl20	4.913	8.662	12.992	1.763	2.644
1428322_a_at	Mm.8688.1	BI905689	Ndufb10	10.799	17.788	28.511	1.647	2.640
1451122_at	Mm.29847.1	BC004801	Idi1	10.151	19.705	26.799	1.941	2.640
1437622_x_at	Mm.27332.2	BB530031	Mrpl28	7.277	9.638	19.211	1.324	2.640
1448419_at	Mm.22284.1	NM_025390	Pop4	2.312	4.516	6.097	1.954	2.637
1452308_a_at	Mm.193539.2	BB462665	Atp1a2	13.686	2.537	36.085	0.185	2.637
1424488_a_at	Mm.212.1	BC011417	1110013G13Rik	3.798	4.950	10.009	1.303	2.636
1416337_at	Mm.24805.1	NM_026219	Uqcrb	11.823	15.914	31.159	1.346	2.635
1420889_at	Mm.10001.1	BB129992	Hccs	1.507	2.310	3.970	1.532	2.634
1417303_at	Mm.28146.1	NM_138656	Mvd	1.937	3.652	5.102	1.886	2.634
1422504_at	Mm.29422.1	NM_010298	Glrb	1.294	2.094	3.408	1.618	2.633
1431183_at	Mm.67218.1	AA109251	1700066M21Rik	1.868	1.185	4.915	0.635	2.631
1448563_at	Mm.2355.1	AK010619	Phb	2.358	3.916	6.203	1.661	2.631
1456475_s_at	Mm.25594.2	BB216074	Prkar2b	1.445	1.558	3.795	1.079	2.627
1434273_at	Mm.4143.2	BG073439	A830073O21Rik	3.146	6.289	8.257	1.999	2.625
1427488_a_at	Mm.12921.2	BC026990	Birc6	0.471	0.682	1.236	1.449	2.624
1417123_at	Mm.103805.1	BC027242	Vav3	0.938	0.628	2.458	0.670	2.622
1438120_x_at	Mm.38241.3	AV091354	Irak1	0.965	0.979	2.529	1.015	2.621
1416447_at	Mm.171292.1	BE986812	2010200I23Rik	4.336	7.195	11.362	1.659	2.620
1428134_at	Mm.29042.1	AK004527	2310005O14Rik	5.930	6.923	15.533	1.167	2.619
1415891_at	Mm.29845.1	NM_019879	Suclg1	9.011	17.155	23.560	1.904	2.615
1438476_a_at	Mm.183257.4	BB201828	Chd4	1.010	1.954	2.642	1.934	2.614
1441910_x_at	Mm.128432.1	BB293079	Ccne1	1.571	1.810	4.104	1.152	2.612
1448292_at	Mm.43162.1	NM_025650	Uqcr	19.107	28.878	49.890	1.511	2.611
1447808_s_at	Mm.124505.1	BB244383	Slc15a2	3.787	1.500	9.887	0.396	2.611
1437907_a_at	Mm.1863.3	BB559082	Tbca	11.163	21.842	29.118	1.957	2.608
1424573_at	Mm.117055.1	BC020076	4432412D15Rik	1.440	2.555	3.756	1.774	2.608
1435589_at	Mm.29875.1	AV308222	MGC67311	1.737	3.195	4.530	1.839	2.608
1418503_at	Mm.2849.1	D11089	Hspa9a	6.255	10.278	16.306	1.643	2.607

1425077_at	Mm.108596.1	AI606403	2700075B01Rik	0.491	0.991	1.279	2.017	2.605
1432344_a_at	Mm.19133.3	AK013376	Aplp2	3.081	5.673	8.026	1.841	2.605
1438635_x_at	Mm.132095.1	BB258476	B930041F14Rik	2.079	3.015	5.412	1.450	2.604
1429839_a_at	Mm.4714.2	AK012787	Yaf2	7.074	11.392	18.414	1.610	2.603
1423757_x_at	Mm.22248.2	BC019836	Igfbp4	1.906	0.881	4.962	0.462	2.603
1423696_a_at	Mm.27591.1	BC006869	2400006A19Rik	8.805	16.619	22.894	1.888	2.600
1433984_a_at	Mm.21743.2	AV016940	Mdh2	30.009	52.053	78.010	1.735	2.600
1448654_at	Mm.28023.1	BI872421	Mtch2	3.817	7.482	9.920	1.960	2.599
1456664_x_at	Mm.196608.4	AV217426	4833420I20Rik	21.742	43.259	56.453	1.990	2.596
1430989_a_at	Mm.196140.2	AK006166	1700020I14Rik	1.240	2.150	3.219	1.733	2.596
1419925_s_at	Mm.204548.1	AV259382	A730024F05Rik	3.926	7.431	10.145	1.893	2.584
1452026_a_at	Mm.22526.2	AY007382	Pla2g12a	0.530	0.719	1.368	1.355	2.578
1454724_x_at	Mm.28796.2	AV173092	5730446C15Rik	8.685	13.549	22.356	1.560	2.574
1438563_s_at	Mm.7328.2	AV069725	Mrps24	3.630	6.092	9.342	1.678	2.573
1424064_at	Mm.182563.1	BC016408	1110011F09Rik	5.224	9.784	13.428	1.873	2.570
1422574_at	Mm.38795.1	BE291523	Mad4	0.686	1.021	1.763	1.488	2.570
1433442_at	Mm.2500.1	BG064945	C530050O22Rik	10.943	19.306	28.117	1.764	2.569
1453111_a_at	Mm.44226.2	AK019396	3010027G13Rik	5.090	7.936	13.059	1.559	2.566
1456590_x_at	Mm.451.5	BB469763	Akr1b3	2.193	3.585	5.620	1.635	2.563
1428351_at	Mm.23018.1	AK013149	2810423O19Rik	1.680	2.929	4.305	1.743	2.563
1434422_at	Mm.23446.1	AV027153	AI428479	6.458	5.435	16.545	0.842	2.562
1431691_a_at	Mm.29274.2	AK007048	1700093E07Rik	5.454	8.974	13.952	1.646	2.558
1456015_x_at	Mm.29842.2	AW228218	Ndufv1	9.712	13.769	24.836	1.418	2.557
1418746_at	Mm.177272.1	NM_019999	Brp17	3.941	4.701	10.078	1.193	2.557
1416867_at	Mm.23564.1	NM_009748	Bet1	1.029	1.905	2.632	1.850	2.557
1430474_a_at	Mm.28023.3	AK007757	Mtch2	10.451	20.584	26.699	1.970	2.555
1437343_x_at	Mm.21881.2	AV239773	Atad3a	4.508	5.682	11.510	1.260	2.553
1440958_at	Mm.29322.1	BF462492	D930017K21Rik	0.655	1.016	1.673	1.550	2.553
1433623_at	Mm.33008.1	BE629588	Zfp367	1.292	1.778	3.296	1.376	2.550
1415727_at	Mm.205996.1	AV017766	Apoa1bp	10.541	19.408	26.847	1.841	2.547
1452607_at	Mm.41102.1	BB410125	2610030H06Rik	4.165	8.200	10.602	1.969	2.546
1426689_s_at	Mm.22149.1	BF585357	Sdha	11.793	18.877	30.004	1.601	2.544
1451042_a_at	Mm.196204.1	AK013451	Mina	1.032	1.475	2.624	1.430	2.544
1428106_at	Mm.214574.1	AK004841	1300001I01Rik	2.502	4.982	6.361	1.991	2.542
1456071_a_at	Mm.35389.3	AV155488	Cycs	24.578	32.956	62.450	1.341	2.541
1416345_at	Mm.30034.1	W82151	Timm8a	4.447	6.124	11.297	1.377	2.540
1424613_at	Mm.103439.1	BC020004	Gprc5b	2.132	4.036	5.413	1.893	2.539
1433989_at	Mm.44683.1	BB536871	Gabt4	6.798	0.948	17.260	0.139	2.539
1427206_at	Mm.39751.1	AW546149	Afg3l2	0.592	1.026	1.503	1.733	2.539
1424425_a_at	Mm.28500.1	BG075139	Mtap	4.054	6.748	10.291	1.665	2.539
1423710_at	Mm.28365.1	BC006702	Dlst	7.628	12.404	19.353	1.626	2.537
1449267_at	Mm.41767.1	BC006876	3110023E09Rik	1.318	2.515	3.343	1.909	2.537
1452191_at	Mm.39856.1	AK011112	2510048K03Rik	0.695	0.560	1.760	0.807	2.535
1452272_a_at	Mm.28124.2	BI901126	Gfer	2.675	4.134	6.778	1.546	2.534
1453589_a_at	Mm.1571.2	BI737178	2610005L07Rik	0.781	1.239	1.980	1.585	2.534
1429314_at	Mm.218599.1	AK018343	Syt11	16.023	30.882	40.591	1.927	2.533
1457744_at	Mm.212498.1	BG243238	Ddx46	0.717	0.748	1.815	1.043	2.531
1431197_at	Mm.175403.1	BB641782	2010110I21Rik	0.478	0.754	1.210	1.577	2.529
1432218_a_at	Mm.132646.2	AK020079	4632412I24Rik	0.580	0.750	1.467	1.293	2.528
1453726_s_at	Mm.218294.1	AK013022	2810407C02Rik	8.121	15.535	20.519	1.913	2.527
1417252_at	Mm.41722.1	NM_015807	Nt5c	4.967	8.997	12.550	1.811	2.527
1426343_at	Mm.29697.1	AK018758	1300006C19Rik	3.466	5.423	8.750	1.565	2.525

1448253_at	Mm.10600.1	NM_008133	Glud	26.485	48.619	66.644	1.836	2.516
1456759_at	Mm.39812.1	BE953888	6430556C10Rik	0.529	0.866	1.332	1.636	2.516
1448810_at	Mm.30193.1	BC015277	Gne	1.263	1.112	3.176	0.881	2.516
1433655_at	Mm.96867.1	AV024351	Rnf141	4.618	8.865	11.611	1.920	2.514
1415733_a_at	Mm.24228.2	BI080296	1110019J04Rik	7.628	10.549	19.176	1.383	2.514
1437194_x_at	Mm.3094.2	BB252479	1200011O22Rik	3.173	5.035	7.967	1.587	2.511
1417559_at	Mm.12983.1	BB478992	Sfxn1	6.645	5.254	16.684	0.791	2.511
1427921_s_at	Mm.4011.1	AW045976	1110046L09Rik	2.774	2.334	6.962	0.841	2.510
1441931_x_at	Mm.90304.1	BB125219	Gss	0.769	1.214	1.927	1.578	2.505
1438794_x_at	Mm.14798.11	AI642440	Rps13	18.070	23.775	45.249	1.316	2.504
1434748_at	Mm.22448.1	BM208103	Ckap2	6.281	1.855	15.722	0.295	2.503
1422824_s_at	Mm.2012.1	NM_007945	Eps8	3.599	2.936	9.009	0.816	2.503
1460319_at	Mm.35628.1	NM_016893	Fut8	4.761	5.889	11.918	1.237	2.503
1434648_a_at	Mm.741.2	BI903794	Ccm2	7.762	13.648	19.422	1.758	2.502
1425092_at	Mm.40247.1	AF183946	A830016G23Rik	3.075	2.625	7.692	0.854	2.501
1426259_at	Mm.29566.1	BC027089	Pank3	1.044	1.692	2.610	1.621	2.501
1450214_at	Mm.3938.1	NM_007413	Adora2b	0.483	0.213	1.207	0.441	2.500
1451345_at	Mm.28500.1	BG075139	Mtap	1.956	2.579	4.882	1.318	2.496
1422501_s_at	Mm.196043.1	NM_029573	Idh3a	1.775	1.518	4.426	0.855	2.493
1452626_a_at	Mm.41915.1	AK007494	1810014F10Rik	0.946	1.529	2.356	1.615	2.490
1455357_x_at	Mm.6932.2	BF228097	Tomm20	14.654	21.844	36.482	1.491	2.489
1418143_at	Mm.2582.1	NM_013841	Vps45	1.767	2.890	4.396	1.635	2.487
1428588_a_at	Mm.23010.1	BF134369	2810443J12Rik	5.661	9.183	14.049	1.622	2.482
1422627_a_at	Mm.26763.1	BF581250	Mkks	3.642	4.990	9.033	1.370	2.480
1452206_at	Mm.19154.1	AK005273	Sucla2	10.290	15.917	25.513	1.547	2.479
1450861_at	Mm.4683.1	BE952454	Fancc	0.409	0.710	1.014	1.735	2.478
1417169_at	Mm.12914.1	AI553394	Usp2	2.010	2.964	4.978	1.475	2.477
1428281_at	Mm.32746.1	AK011362	2610009I02Rik	0.738	1.015	1.826	1.376	2.475
1448274_at	Mm.30049.1	NM_007573	C1qbp	9.305	10.956	23.034	1.177	2.475
1456438_x_at	Mm.22333.4	BB145729	Rpn1	16.325	27.641	40.363	1.693	2.472
1437108_at	Mm.28694.2	BE949068	Lsm6	1.207	1.311	2.985	1.085	2.472
1423972_at	Mm.26949.1	BC003432	Etfa	10.577	18.399	26.116	1.739	2.469
1435859_x_at	Mm.2462.2	AV263662	Psmc2	13.111	25.542	32.368	1.948	2.469
1423159_at	Mm.3131.1	AI647805	Dld	5.368	7.556	13.249	1.408	2.468
1428635_at	Mm.11827.1	AK007659	1810030M08Rik	1.275	0.811	3.146	0.637	2.468
1427140_at	Mm.4608.2	BE956863	Pvt1	0.483	0.563	1.192	1.164	2.465
1417205_at	Mm.181880.1	AJ278133	Kdelr2	2.690	3.623	6.632	1.347	2.465
1449106_at	Mm.7156.1	NM_008161	Gpx3	1.240	0.522	3.055	0.421	2.464
1451274_at	Mm.30074.1	BC013670	Ogdh	2.808	4.913	6.915	1.749	2.462
1417548_at	Mm.29594.1	BB546730	Sart3	0.537	0.664	1.322	1.236	2.461
1417902_at	Mm.35444.1	NM_054087	Slc19a2	2.040	2.551	5.016	1.251	2.459
1437593_x_at	Mm.692.3	BB449248	Api5	14.779	26.946	36.334	1.823	2.458
1451327_a_at	Mm.196618.1	BC016568	Il1rl1l	4.030	6.310	9.901	1.566	2.457
1424980_s_at	Mm.34556.2	BC012406	6530402N02Rik	5.581	10.089	13.707	1.808	2.456
1417591_at	Mm.28048.1	NM_133783	Ptges2	1.676	2.049	4.115	1.222	2.455
1415917_at	Mm.29584.1	NM_138745	Mthfd1	4.283	6.082	10.499	1.420	2.451
1456580_s_at	Mm.22514.3	AV019894	Atp5d	28.448	44.250	69.714	1.555	2.451
1460697_s_at	Mm.182650.1	BC027564	2610209M04Rik	11.527	22.361	28.247	1.940	2.450
1432372_a_at	Mm.28393.2	AK004941	Spr	1.842	1.969	4.514	1.069	2.450
1434299_x_at	Mm.30191.2	AI413098	Rabl4	3.858	5.902	9.451	1.530	2.450
1423652_at	Mm.7884.1	AV209097	Hbld2	2.312	3.543	5.663	1.532	2.450
1438369_x_at	Mm.21915.3	AV008714	2610318K02Rik	0.658	1.147	1.612	1.742	2.448

1449557_at	Mm.22641.1	NM_025904	1600012F09Rik	1.440	2.133	3.524	1.481	2.448
1432181_s_at	Mm.219419.2	AK013765	Ecgf1	1.216	1.551	2.977	1.276	2.448
1451208_at	Mm.3845.1	BC013717	Etf1	2.292	3.480	5.610	1.518	2.447
1436930_x_at	Mm.1710.2	BB000512	Hmbs	1.164	1.673	2.847	1.438	2.447
1435757_a_at	Mm.988.5	AV082188	Uqcrc2	11.803	15.602	28.861	1.322	2.445
1426616_at	Mm.28122.1	BE951033	0610030G03Rik	0.490	0.493	1.197	1.006	2.445
1419917_s_at	Mm.202023.1	AW545765	3930401E15Rik	7.741	12.323	18.905	1.592	2.442
1453240_a_at	Mm.196347.1	BB698883	2900054P12Rik	2.323	2.746	5.673	1.182	2.442
1451418_a_at	Mm.33268.1	BC023083	Ssb4	3.690	1.276	9.006	0.346	2.441
1416180_a_at	Mm.7051.1	NM_009041	Rdx	2.364	4.459	5.771	1.886	2.441
1416446_at	Mm.171292.1	BE986812	2010200I23Rik	3.427	6.006	8.363	1.753	2.441
1424142_at	Mm.46573.1	AF367244	Ikbkap	0.914	1.690	2.230	1.849	2.441
1416165_at	Mm.29274.1	NM_133685	1700093E07Rik	21.378	38.138	52.132	1.784	2.439
1428047_s_at	Mm.919.3	BC028506	Zfa	0.410	0.592	1.000	1.444	2.438
1417368_s_at	Mm.29867.1	NM_010885	Ndufa2	16.110	28.911	39.275	1.795	2.438
1418611_at	Mm.2514.1	NM_013533	Grca	2.380	1.608	5.800	0.676	2.437
1429568_x_at	Mm.44214.3	AK007937	2510010F15Rik	2.774	4.433	6.760	1.598	2.437
1438194_at	Mm.26627.1	AW488243	2900019G14Rik	5.998	6.738	14.608	1.123	2.435
1460599_at	Mm.83618.1	BB372567	D19Wsu12e	0.705	0.654	1.717	0.927	2.435
1427098_at	Mm.78312.1	BI102531	8030445B08Rik	1.307	2.184	3.182	1.671	2.435
1428495_at	Mm.41894.1	BF300229	2410003K15Rik	3.975	5.428	9.675	1.365	2.434
1423461_a_at	Mm.21846.1	AV328436	Ubl3	7.089	13.853	17.249	1.954	2.433
1424415_s_at	Mm.37938.1	BC020531	Spon1	1.156	1.182	2.813	1.022	2.433
1424095_at	Mm.34038.1	BC016519	Rtcd1	5.533	10.992	13.458	1.987	2.432
1415916_a_at	Mm.29584.1	NM_138745	Mthfd1	7.480	11.148	18.188	1.490	2.431
1427230_at	Mm.29083.1	BB322737	B930041F14Rik	1.009	1.129	2.452	1.120	2.431
1438964_x_at	Mm.760.2	AV096788	Tfpt	0.645	1.201	1.568	1.862	2.430
1438005_at	Mm.87257.1	BB480175	B230315F11Rik	0.680	1.174	1.653	1.726	2.430
1416631_at	Mm.29234.1	NM_026193	1810038H16Rik	1.071	2.029	2.603	1.894	2.430
1438943_x_at	Mm.22333.6	AV308148	Rpn1	13.390	21.609	32.485	1.614	2.426
1453767_a_at	Mm.41761.2	AK008226	Nt5m	3.718	5.426	9.019	1.459	2.426
1416604_at	Mm.29196.1	NM_025567	Cyc1	25.042	31.519	60.735	1.259	2.425
1427950_at	Mm.162637.1	BM231649	Zfp294	0.594	0.971	1.441	1.634	2.424
1456573_x_at	Mm.3842.4	BB205930	Nnt	3.139	5.721	7.607	1.823	2.424
1417136_s_at	Mm.8709.1	NM_009274	Srpk2	2.732	3.921	6.620	1.435	2.423
1416837_at	Mm.19904.1	BC018228	Bax	11.212	15.593	27.163	1.391	2.423
1452216_at	Mm.37560.1	BB494388	BC025519	1.953	2.920	4.732	1.495	2.423
1428488_at	Mm.45561.1	BB646655	Pigk	1.623	3.016	3.932	1.858	2.422
1457669_x_at	Mm.212751.1	AV096765	Rfc2	0.748	0.742	1.811	0.993	2.422
1419552_at	Mm.41728.1	NM_025855	Echdc1	3.697	7.164	8.951	1.938	2.421
1436200_at	Mm.30731.1	BE956940	A830039N02Rik	1.560	1.053	3.777	0.675	2.421
1430515_s_at	Mm.195973.1	AK008554	Aasdhppt	2.163	3.105	5.229	1.436	2.418
1454960_at	Mm.28611.1	BI646741	Smad3	2.017	2.254	4.875	1.117	2.417
1437633_at	Mm.41929.2	BB320513	3010027A04Rik	1.174	1.768	2.836	1.507	2.416
1451038_at	Mm.29262.1	BB819942	Apln	1.221	0.727	2.951	0.595	2.416
1448373_at	Mm.21356.1	NM_026310	Mrpl18	3.681	3.872	8.892	1.052	2.416
1431530_a_at	Mm.31927.2	AK015705	Tm4sf9	1.290	0.965	3.116	0.748	2.415
1435841_s_at	Mm.196456.1	BB757840	Suclg2	7.403	8.242	17.870	1.113	2.414
1433766_at	Mm.26878.1	BG065438	C330023M02Rik	1.124	1.693	2.713	1.506	2.413
1449972_s_at	Mm.4596.1	NM_011765	Zfp97	0.821	1.189	1.980	1.448	2.412
1433779_at	Mm.31922.1	AV311104	D130060C09Rik	6.048	10.739	14.581	1.776	2.411
1451631_at	Mm.216371.1	BC021395	BC021395	0.529	0.845	1.274	1.599	2.410

1452800_a_at	Mm.28628.1	AK002320	0610008C08Rik	7.941	12.415	19.135	1.563	2.410
1460464_at	Mm.608.1	AV030118	2700089E24Rik	2.901	4.594	6.990	1.583	2.409
1423158_at	Mm.41304.1	AK008566	Gnpnat1	0.611	1.074	1.471	1.758	2.408
1418585_at	Mm.18474.1	NM_023243	Ccnh	1.289	1.990	3.103	1.544	2.407
1417322_at	Mm.4022.1	NM_133964	1110033C18Rik	2.094	3.086	5.040	1.474	2.406
1418371_at	Mm.29468.1	AY029255	6720463E02Rik	3.824	7.037	9.199	1.840	2.406
1450080_at	Mm.158897.1	NM_028375	2900027G03Rik	2.751	4.396	6.619	1.598	2.406
1448426_at	Mm.141020.1	BI217574	Sardh	1.060	1.568	2.549	1.480	2.405
1423829_at	Mm.22383.1	BC011343	0910001A06Rik	5.985	9.258	14.387	1.547	2.404
1427958_at	Mm.22955.1	BB478745	D230019K24Rik	0.849	1.664	2.040	1.959	2.402
1426510_at	Mm.136458.1	AW537824	C330023F11Rik	3.064	5.907	7.361	1.927	2.402
1448028_at	Mm.216274.1	BF536757	C530046L02Rik	1.486	2.668	3.570	1.795	2.402
1434709_at	Mm.37263.1	BB202655	C130076O07Rik	5.871	10.434	14.100	1.777	2.402
1415933_a_at	Mm.360.1	NM_007747	Cox5a	33.629	54.327	80.698	1.616	2.400
1416679_at	Mm.1519.1	BC009119	Abcd3	17.208	33.154	41.273	1.927	2.399
1423840_at	Mm.52.1	BC026206	D11Ertd99e	3.489	5.085	8.359	1.458	2.396
1429122_a_at	Mm.28037.1	AK006658	1700040I03Rik	1.347	1.347	3.225	1.000	2.395
1429429_s_at	Mm.196522.1	AK018402	A030012M09Rik	1.255	2.457	3.006	1.957	2.395
1435755_at	Mm.181765.2	BB609468	1110001A16Rik	2.446	4.253	5.855	1.739	2.394
1424662_at	Mm.182067.2	AK004549	4933435E07Rik	3.647	5.971	8.730	1.637	2.394
1418716_at	Mm.87062.1	AK004037	Mrps25	0.835	1.224	1.999	1.465	2.393
1424086_at	Mm.3776.1	BC025514	D130038B21Rik	0.726	1.125	1.737	1.550	2.393
1454642_a_at	Mm.14486.1	BB230296	Commd3	3.806	7.163	9.104	1.882	2.392
1436931_at	Mm.32654.1	AV255458	Rfx4	10.132	18.005	24.235	1.777	2.392
1435333_at	Mm.28983.2	AV156568	1110007M04Rik	1.803	2.106	4.311	1.168	2.392
1421604_a_at	Mm.28787.1	NM_008453	Klf3	0.898	0.876	2.148	0.976	2.391
1455777_x_at	Mm.3195.2	BB032410	Hsd17b4	8.667	15.654	20.714	1.806	2.390
1460698_a_at	Mm.27800.1	AK007641	1810029G24Rik	4.500	5.659	10.755	1.258	2.390
1429213_at	Mm.46585.1	AK009547	2310030N02Rik	0.991	0.996	2.368	1.006	2.390
1437238_x_at	Mm.21062.3	BB233370	C87860	2.122	2.904	5.072	1.369	2.390
1449194_at	Mm.87062.1	AK004037	Mrps25	3.594	5.389	8.589	1.499	2.390
1433936_at	Mm.6844.1	BG791555	0610010E21Rik	3.257	4.208	7.776	1.292	2.388
1426657_s_at	Mm.16898.1	L21027	Phgdh	7.121	11.571	17.000	1.625	2.387
1422559_at	Mm.30233.1	AY039837	Ube2n	12.299	24.524	29.349	1.994	2.386
1416949_s_at	Mm.18556.1	NM_008202	Slc39a7	6.036	9.542	14.403	1.581	2.386
1450174_at	Mm.29220.1	NM_021464	Ptprt	0.461	0.330	1.099	0.717	2.386
1437289_at	Mm.24703.1	BG797423	1110001C20Rik	5.568	9.540	13.272	1.714	2.384
1435517_x_at	Mm.27832.3	BB465250	Ralb	3.929	6.403	9.356	1.629	2.381
1421992_a_at	Mm.22248.1	NM_010517	Igfbp4	1.218	0.134	2.899	0.110	2.381
1439267_x_at	Mm.360.4	AV085180	Cox5a	21.597	30.998	51.407	1.435	2.380
1434437_x_at	Mm.99.2	AV301324	Rrm2	14.087	4.715	33.466	0.335	2.376
1452462_a_at	Mm.3594.3	BC013339	Banp	0.684	1.332	1.623	1.949	2.374
1452085_at	Mm.178424.2	BE134386	Odag	2.838	4.215	6.733	1.485	2.373
1423812_s_at	Mm.24642.1	BC024822	AW146242	2.872	4.601	6.803	1.602	2.369
1455875_x_at	Mm.5885.3	BB131843	Tm9sf2	14.970	29.783	35.456	1.990	2.369
1422732_at	Mm.197493.1	NM_026389	Poldip2	5.877	10.826	13.910	1.842	2.367
1459902_at	Mm.107715.1	AI510297	2700007P21Rik	0.741	1.466	1.754	1.978	2.367
1427464_s_at	Mm.918.2	AJ002387	Hspa5	10.551	19.045	24.970	1.805	2.367
1423407_a_at	Mm.6120.1	BF228318	Fbln2	3.985	0.089	9.429	0.022	2.366
1425051_at	Mm.182574.1	AK010892	2610034N03Rik	1.949	1.624	4.611	0.834	2.366
1433616_a_at	Mm.29828.2	BG793007	2310028O11Rik	14.545	19.830	34.403	1.363	2.365
1423492_at	Mm.5381.1	C79823	Mrpl45	3.488	5.744	8.250	1.647	2.365

1416433_at	Mm.2870.1	BC004578	Rpa2	1.911	1.765	4.519	0.924	2.365
1431033_x_at	Mm.17221.1	AA681807	Agl	2.108	2.915	4.986	1.383	2.365
1434099_at	Mm.35687.3	BB752393	A830037N07Rik	1.086	0.971	2.565	0.894	2.362
1449712_s_at	Mm.195299.1	C85064	Atp6v1e1	12.473	24.484	29.440	1.963	2.360
1451354_at	Mm.138512.1	BC024806	BC024806	1.006	1.282	2.372	1.274	2.359
1418949_at	Mm.31325.1	NM_011819	Gdf15	0.572	0.202	1.350	0.354	2.358
1417972_s_at	Mm.24636.1	NM_026398	Pop5	4.462	4.878	10.522	1.093	2.358
1452722_a_at	Mm.46579.1	BB702110	Cul5	0.743	1.109	1.752	1.493	2.358
1455176_a_at	Mm.28873.4	BF457392	Syt11	14.976	26.284	35.299	1.755	2.357
1416666_at	Mm.3093.1	NM_009255	Serpine2	32.506	52.657	76.618	1.620	2.357
1435694_at	Mm.26053.1	BB127065	4933432P15Rik	0.489	0.350	1.153	0.716	2.357
1419522_at	Mm.24730.1	NM_026021	Zmynd19	1.263	1.439	2.976	1.139	2.357
1449209_a_at	Mm.219462.1	AB030503	Rdh11	2.547	4.239	5.997	1.664	2.354
1428675_at	Mm.29131.1	AK004208	1110049F12Rik	3.117	4.150	7.337	1.331	2.354
1448264_a_at	Mm.104828.1	NM_018799	Eif3s2	11.652	16.502	27.419	1.416	2.353
1455886_at	Mm.8585.2	BB385581	Cbl	1.196	1.195	2.812	0.999	2.351
1455737_at	Mm.70065.1	AU040848	C030002B11Rik	0.622	0.967	1.463	1.553	2.351
1434006_at	Mm.213010.1	BQ030992	BC051227	2.930	5.565	6.888	1.899	2.350
1451056_at	Mm.18347.1	BB034143	Psmd7	4.623	8.158	10.860	1.765	2.349
1452791_at	Mm.46631.1	AK009092	2310002F18Rik	3.197	5.111	7.508	1.599	2.348
1451002_at	Mm.154581.1	AA034553	Aco2	25.705	35.392	60.361	1.377	2.348
1423138_at	Mm.143771.1	BE854862	Wdr4	0.656	1.111	1.541	1.694	2.348
1456700_x_at	Mm.30059.7	BB100920	Marcks	25.303	41.457	59.345	1.638	2.345
1428772_at	Mm.25042.1	AK003432	1110004L07Rik	4.011	5.604	9.400	1.397	2.343
1452192_at	Mm.22496.1	BM502329	BC053440	3.296	6.504	7.724	1.973	2.343
1432646_a_at	Mm.15985.3	BE859789	2900097C17Rik	1.556	2.396	3.647	1.540	2.343
1415840_at	Mm.19130.1	NM_134255	Elovl5	14.348	24.963	33.608	1.740	2.342
1416616_s_at	Mm.15243.1	NM_017393	Clpp	3.583	5.309	8.394	1.481	2.342
1437008_x_at	Mm.30020.2	BB477208	1110006I15Rik	2.559	3.714	5.991	1.451	2.341
1438096_a_at	Mm.3856.3	AV306250	Dtymk	2.379	3.040	5.567	1.278	2.340
1434512_x_at	Mm.6787.2	BB492363	Sfrs3	7.965	9.431	18.634	1.184	2.339
1417144_at	Mm.43167.1	NM_134024	Tubg1	3.090	3.126	7.227	1.012	2.339
1426495_at	Mm.46449.1	BQ031311	2410042D21Rik	1.683	3.058	3.935	1.817	2.337
1460689_at	Mm.22123.2	BC022097	D15Wsu75e	3.886	6.409	9.080	1.649	2.337
1428970_at	Mm.197688.1	AV113878	Mak3	2.801	4.310	6.545	1.538	2.336
1439783_at	Mm.24318.1	BB809870	C330018D20Rik	0.778	1.254	1.818	1.611	2.336
1452132_at	Mm.28122.1	BE951033	0610030G03Rik	3.187	3.282	7.445	1.030	2.336
1456517_at	Mm.116728.1	BB035891	9330161C17Rik	0.839	0.628	1.959	0.749	2.336
1419089_at	Mm.4871.1	BI111620	Timp3	1.951	3.522	4.551	1.806	2.333
1425052_at	Mm.182574.1	AK010892	2610034N03Rik	4.013	2.560	9.362	0.638	2.333
1452737_at	Mm.29464.1	AK012692	2810008M24Rik	8.178	13.373	19.075	1.635	2.332
1418323_at	Mm.24069.1	BM232562	Fem1b	0.706	0.931	1.646	1.319	2.332
1427885_at	Mm.22884.1	AK010477	Pold4	1.329	1.869	3.098	1.406	2.332
1417019_a_at	Mm.20912.1	NM_011799	Cdc6	0.470	0.108	1.095	0.230	2.331
1428870_at	Mm.78861.2	BM213850	Nolc1	1.833	2.937	4.272	1.603	2.331
1453725_a_at	Mm.193040.2	AK012225	Mrps7	4.042	5.342	9.418	1.321	2.330
1455841_s_at	Mm.21070.2	BB251524	Grwd1	1.483	1.834	3.455	1.237	2.330
1419494_a_at	Mm.2777.1	BC002036	Tpd52	1.334	2.549	3.108	1.910	2.329
1460235_at	Mm.39288.1	NM_007644	Scarb2	5.495	9.364	12.797	1.704	2.329
1452137_at	Mm.28214.1	BB704602	Acbd3	0.823	1.476	1.917	1.793	2.329
1450866_a_at	Mm.44225.1	BQ179556	Mrpl17	8.782	11.895	20.448	1.355	2.329
1424038_a_at	Mm.29937.1	BC020179	2310044H10Rik	12.232	22.455	28.479	1.836	2.328

1429211_at	Mm.113827.1	AK013801	2900078E11Rik	1.276	2.265	2.969	1.776	2.327
1433966_x_at	Mm.2942.2	AV212753	Asns	1.834	2.562	4.263	1.397	2.325
1421971_a_at	Mm.29741.1	NM_023260	Mrps34	7.070	11.037	16.432	1.561	2.324
1431105_a_at	Mm.110600.4	AK012413	1600019D15Rik	3.089	5.512	7.180	1.784	2.324
1416580_a_at	Mm.29000.1	NM_019719	Stub1	8.386	13.960	19.489	1.665	2.324
1438634_x_at	Mm.200673.8	BB105164	Lasp1	6.169	12.147	14.329	1.969	2.323
1417819_at	Mm.206786.1	BB004887	Tor1b	0.938	1.492	2.179	1.590	2.322
1452588_at	Mm.20301.1	BC027683	2810407K09Rik	0.814	1.127	1.889	1.384	2.321
1428069_at	Mm.29164.1	AK011289	Cdca7	1.573	0.458	3.651	0.291	2.321
1418444_a_at	Mm.30126.1	BC003902	Mir16	5.859	11.636	13.601	1.986	2.321
1438510_a_at	Mm.10528.2	BB451746	Hars	5.284	10.247	12.266	1.939	2.321
1433661_at	Mm.3957.1	BB830346	BC034204	1.305	1.263	3.029	0.968	2.321
1433729_x_at	Mm.34774.2	AV047320	3110004O18Rik	6.149	7.179	14.270	1.168	2.321
1456088_at	Mm.149115.6	BF134200	Birc4	1.019	1.788	2.364	1.755	2.320
1438546_x_at	Mm.658.8	C81442	Slc25a5	15.992	14.556	37.102	0.910	2.320
1435735_x_at	Mm.22362.3	BB285733	H47	2.927	5.514	6.790	1.884	2.319
1416998_at	Mm.29061.1	NM_021511	Rrs1	3.838	6.162	8.901	1.605	2.319
1415738_at	Mm.31076.1	NM_025334	0610040B21Rik	7.077	8.458	16.407	1.195	2.318
1428195_at	Mm.28589.2	BG072404	4631427C17Rik	0.609	0.949	1.412	1.558	2.317
1452189_at	Mm.137912.1	BG865652	9430077D24Rik	7.691	13.277	17.813	1.726	2.316
1452902_at	Mm.27121.1	AK011939	2610209N15Rik	1.983	2.124	4.589	1.071	2.314
1448211_at	Mm.30214.1	NM_133764	0610006O14Rik	12.222	23.850	28.275	1.951	2.314
1450312_at	Mm.103640.1	NM_011800	Cdh20	1.848	0.664	4.274	0.359	2.313
1434893_at	Mm.193539.4	AI845177	Atp1a2	4.623	0.946	10.694	0.205	2.313
1428163_at	Mm.196592.1	AK010187	Sara2	13.554	26.689	31.351	1.969	2.313
1438545_at	Mm.658.8	C81442	Slc25a5	5.310	2.977	12.273	0.561	2.311
1416788_a_at	Mm.14825.1	NM_008323	Idh3g	13.014	17.559	30.077	1.349	2.311
1430449_at	Mm.133458.1	AK021172	C330002I19Rik	0.464	0.980	1.071	2.113	2.310
1418760_at	Mm.219462.1	AB030503	Rdh11	2.716	4.642	6.269	1.709	2.308
1450743_s_at	Mm.196461.1	BG920261	Syncrip	2.508	2.767	5.781	1.104	2.306
1456036_x_at	Mm.282.3	AV003026	Gsto1	2.891	5.503	6.665	1.903	2.305
1424226_at	Mm.218590.1	BC021385	9030617O03Rik	1.022	0.780	2.355	0.764	2.305
1436917_s_at	Mm.31239.4	BB491018	Gpsm1	1.149	1.737	2.646	1.511	2.302
1425830_a_at	Mm.20693.2	BC021867	2810452K22Rik	2.067	3.051	4.758	1.476	2.302
1451671_at	Mm.27339.1	AV094567	Gorasp1	1.035	1.791	2.381	1.730	2.300
1438480_a_at	Mm.16925.2	AV301675	Thy28	2.841	3.132	6.533	1.102	2.299
1426936_at	Mm.220992.1	BC002257	LOC215866	2.951	2.756	6.782	0.934	2.298
1454860_x_at	Mm.2547.3	BI966630	Dad1	8.885	16.307	20.418	1.835	2.298
1428836_at	Mm.46372.1	AK009046	2300009A05Rik	1.645	2.770	3.778	1.684	2.296
1449504_at	Mm.6952.1	U20619	Kpna1	0.680	1.058	1.560	1.557	2.296
1450860_at	Mm.34832.1	AK010384	Lap3	4.955	9.491	11.373	1.916	2.295
1416060_at	Mm.22252.1	BF577643	Tbc1d15	4.426	6.931	10.156	1.566	2.295
1431334_a_at	Mm.26506.1	AK017049	4933433P14Rik	0.650	1.139	1.491	1.752	2.294
1427692_a_at	Mm.10735.2	Y17137	Cask	1.612	1.916	3.698	1.189	2.294
1423608_at	Mm.193.1	BI966443	Itm2a	4.220	4.480	9.674	1.062	2.292
1422017_s_at	Mm.220315.1	NM_029241	4833439L19Rik	3.528	5.983	8.085	1.696	2.292
1447522_s_at	Mm.207035.1	AI662480	5430432P15Rik	5.362	7.818	12.289	1.458	2.292
1451679_at	Mm.40004.1	BC016270	6530401D17Rik	4.921	4.898	11.278	0.995	2.292
1456584_x_at	Mm.16898.6	BB495884	Phgdh	8.431	13.072	19.323	1.550	2.292
1422744_at	Mm.42254.1	NM_008832	Phka1	0.597	0.670	1.368	1.122	2.291
1437163_x_at	Mm.10182.3	BB168668	Gtf2h4	3.610	4.724	8.266	1.308	2.290
1448904_at	Mm.27673.1	AK016470	D6Wsu176e	3.112	4.602	7.124	1.479	2.289

1421266_s_at	Mm.220333.1	NM_010908	Nfkbib	1.183	2.169	2.709	1.833	2.289
1427044_a_at	Mm.39792.1	BQ177140	Amph	2.789	3.152	6.380	1.130	2.288
1431012_a_at	Mm.28883.2	AK009478	Peci	15.338	24.180	35.086	1.576	2.287
1422811_at	Mm.7206.1	NM_011977	Slc27a1	4.026	5.645	9.208	1.402	2.287
1429763_at	Mm.151104.1	AW547258	D530030D03Rik	8.016	12.831	18.330	1.601	2.287
1428290_at	Mm.38407.1	AK017491	Mipep	1.880	3.000	4.298	1.596	2.286
1434100_x_at	Mm.35687.3	BB752393	A830037N07Rik	1.182	0.947	2.702	0.801	2.286
1448210_at	Mm.14530.1	AW108405	Rab1	6.479	10.727	14.777	1.656	2.281
1436833_x_at	Mm.74226.2	BB251824	Ttll1	2.657	5.270	6.055	1.984	2.279
1449334_at	Mm.4871.1	BI111620	Timp3	0.677	0.952	1.543	1.406	2.279
1418230_a_at	Mm.29097.1	BC005621	Lims1	2.166	3.754	4.937	1.733	2.279
1416209_at	Mm.10600.1	NM_008133	Glud	27.047	45.229	61.604	1.672	2.278
1429193_at	Mm.30115.1	BE952940	4631416I11Rik	3.760	7.199	8.562	1.914	2.277
1426118_a_at	Mm.20843.3	AF109918	Tomm40	4.912	6.472	11.182	1.318	2.276
1448704_s_at	Mm.22362.1	NM_024439	H47	7.101	13.321	16.160	1.876	2.276
1429160_at	Mm.196528.1	AK012733	2810012L14Rik	0.670	0.975	1.524	1.456	2.275
1422500_at	Mm.196043.1	NM_029573	Idh3a	1.129	1.023	2.568	0.906	2.275
1418436_at	Mm.10818.1	AB019212	Stx7	6.510	12.723	14.806	1.954	2.274
1451583_a_at	Mm.41648.1	BC025076	BC025076	0.521	0.898	1.184	1.724	2.274
1434679_at	Mm.4936.2	BM945195	Cspg3	12.986	9.189	29.525	0.708	2.274
1422414_a_at	Mm.18041.1	NM_007589	Calm2	12.775	13.239	29.037	1.036	2.273
1439780_at	Mm.218477.1	BG071958	1500016H10Rik	0.574	0.686	1.304	1.196	2.273
1416668_at	Mm.464.1	NM_025736	4921531G14Rik	7.663	14.455	17.409	1.886	2.272
1416077_at	Mm.1408.1	NM_009627	Adm	0.641	0.472	1.456	0.736	2.270
1456196_x_at	Mm.27941.2	AV038603	Fkbp1a	15.018	28.845	34.094	1.921	2.270
1448527_at	Mm.28441.1	AV094856	Pdcd10	7.048	13.569	15.986	1.925	2.268
1417424_at	Mm.28593.1	BE287896	1110057H19Rik	3.331	4.767	7.554	1.431	2.267
1426743_at	Mm.29665.1	BC002232	Dip3b	10.009	13.813	22.682	1.380	2.266
1423192_at	Mm.20129.1	BB590675	Pspc1	3.200	3.986	7.253	1.246	2.266
1451277_at	Mm.31536.1	BC025897	C530046K17Rik	4.716	6.609	10.686	1.402	2.266
1435395_s_at	Mm.353.2	BG794445	Atp5j2	32.975	47.940	74.713	1.454	2.266
1434240_at	Mm.26468.2	BB463610	4632434I11Rik	0.734	0.180	1.663	0.245	2.266
1428074_at	Mm.8569.1	BE981853	2310037P21Rik	1.537	0.406	3.482	0.264	2.265
1453960_a_at	Mm.2945.3	AK007209	Capzb	7.181	12.449	16.260	1.734	2.264
1417102_a_at	Mm.28058.1	BC025155	Ndufb5	15.169	20.103	34.339	1.325	2.264
1437007_x_at	Mm.27292.2	BB446580	Usp39	5.514	9.576	12.481	1.737	2.264
1418886_s_at	Mm.29590.1	NM_130884	Idh3b	12.774	17.658	28.913	1.382	2.263
1451512_s_at	Mm.25250.1	BC026437	Hibch	3.442	4.237	7.792	1.231	2.263
1437357_at	Mm.30760.1	AI481820	BC037178	0.670	0.459	1.515	0.686	2.263
1441682_s_at	Mm.200928.1	C79645	1110004L07Rik	6.233	8.390	14.105	1.346	2.263
1449024_a_at	Mm.2284.1	U07631	Hexa	11.735	23.273	26.554	1.983	2.263
1423649_at	Mm.24229.1	BC016240	2010300G19Rik	2.300	3.670	5.203	1.596	2.262
1457254_x_at	Mm.119806.1	BB302103	6330442E10Rik	1.211	1.759	2.737	1.453	2.261
1452692_a_at	Mm.2206.1	BI692577	Ndufv2	25.077	32.655	56.688	1.302	2.261
1449964_a_at	Mm.20260.1	NM_019966	Mlycd	1.098	1.687	2.480	1.536	2.259
1439257_x_at	Mm.22333.8	BB519934	Rpn1	18.033	27.648	40.706	1.533	2.257
1415967_at	Mm.29842.1	NM_133666	Ndufv1	13.194	18.035	29.783	1.367	2.257
1416866_at	Mm.23564.1	NM_009748	Bet1	2.081	3.643	4.697	1.751	2.257
1416487_a_at	Mm.4885.1	NM_009534	Yap	0.636	1.227	1.435	1.930	2.256
1455372_at	Mm.203968.3	BB770826	Cpeb3	0.670	0.731	1.511	1.092	2.256
1456865_x_at	Mm.200704.1	AV304745	Rrs1	5.823	7.935	13.131	1.363	2.255
1450864_at	Mm.220898.1	AU079142	Calm3	9.505	17.207	21.433	1.810	2.255

1435020_at	Mm.28959.2	BE980167	Klhdc2	3.210	5.214	7.239	1.624	2.255
1427418_a_at	Mm.3879.2	X95580	Hif1a	11.950	20.612	26.938	1.725	2.254
1438843_x_at	Mm.28023.5	AV067008	Mtch2	4.965	7.171	11.187	1.444	2.253
1460216_at	Mm.18759.1	NM_007383	Acads	3.114	3.890	7.015	1.249	2.253
1437751_at	Mm.10707.2	AV337619	A830037N07Rik	1.396	1.452	3.144	1.040	2.252
1419252_at	Mm.22627.1	BG067649	Eps15	7.244	12.582	16.310	1.737	2.251
1428497_at	Mm.46606.1	AA198634	Secisbp2	0.697	1.109	1.569	1.591	2.251
1428739_at	Mm.34074.1	BB772205	2310040A07Rik	3.879	3.692	8.733	0.952	2.251
1434499_a_at	Mm.9745.2	AV219418	Ldh2	17.720	27.325	39.883	1.542	2.251
1426746_at	Mm.39704.1	BB027373	1810026J23Rik	3.521	6.835	7.922	1.941	2.250
1456090_at	Mm.34499.1	BB667859	Pdhx	2.802	3.795	6.298	1.355	2.248
1450909_at	Mm.3941.1	BB406487	Eif4e	0.672	0.631	1.511	0.939	2.247
1435505_at	Mm.2852.1	BB698273	Dmwd	3.783	7.282	8.490	1.925	2.244
1417499_at	Mm.21826.1	NM_013899	Timm13a	4.816	7.388	10.805	1.534	2.244
1437067_at	Mm.3028.1	BM228625	Phtf2	0.901	0.578	2.021	0.642	2.244
1423879_at	Mm.28101.1	BC020125	D030056L22	2.180	2.895	4.890	1.328	2.244
1418083_at	Mm.35693.1	NM_025319	0610009B22Rik	5.049	8.764	11.328	1.736	2.243
1422577_at	Mm.43822.1	AB056479	Cs	14.858	16.137	33.333	1.086	2.243
1419988_at	Mm.197604.1	C87327	Map3k7	0.546	0.766	1.224	1.403	2.243
1425452_s_at	Mm.5046.1	BC002154	AW125753	4.432	1.644	9.939	0.371	2.243
1454604_s_at	Mm.21950.1	BB072896	Tm4sf12	4.027	5.001	9.030	1.242	2.243
1452698_at	Mm.29900.1	AK009898	2310050B20Rik	1.591	1.491	3.568	0.937	2.242
1434658_at	Mm.3285.1	BB333439	3110056O03Rik	3.392	5.105	7.600	1.505	2.241
1460354_a_at	Mm.10076.1	AB049641	Mrpl13	4.819	5.066	10.799	1.051	2.241
1451133_s_at	Mm.4511.1	BC007160	8430437G11Rik	0.921	1.587	2.064	1.722	2.240
1423181_s_at	Mm.21482.1	AK011789	Clns1a	7.620	10.297	17.061	1.351	2.239
1426742_at	Mm.148721.1	AK019459	Atp5f1	19.481	27.720	43.610	1.423	2.239
1437379_x_at	Mm.182051.3	BB297636	Dnase1	2.694	2.718	6.030	1.009	2.239
1455800_x_at	Mm.29651.2	BB085063	1110030L07Rik	9.926	12.815	22.205	1.291	2.237
1428096_at	Mm.132208.1	AK010877	Ipo11	2.766	3.922	6.187	1.418	2.237
1417038_at	Mm.38450.1	NM_017380	38969	18.921	11.568	42.294	0.611	2.235
1437211_x_at	Mm.19130.3	BB254141	Elovl5	15.857	26.274	35.440	1.657	2.235
1438547_x_at	Mm.200695.4	BB223859	Tor2a	2.580	3.772	5.764	1.462	2.234
1420891_at	Mm.4092.1	W29605	Wnt7b	2.218	2.644	4.956	1.192	2.234
1416020_a_at	Mm.258.1	NM_007506	Atp5g1	14.559	16.653	32.519	1.144	2.234
1434210_s_at	Mm.80542.5	AV174595	Slc25a26	11.371	16.166	25.392	1.422	2.233
1420479_a_at	Mm.3797.1	BG064031	Nap1l1	12.511	12.119	27.935	0.969	2.233
1455545_at	Mm.24388.2	BI415815	1110065P20Rik	7.314	8.113	16.327	1.109	2.232
1423418_at	Mm.39472.1	BI247584	Fdps	17.882	29.087	39.904	1.627	2.232
1448183_a_at	Mm.3879.1	BB269715	Hif1a	1.704	2.188	3.803	1.283	2.231
1415990_at	Mm.569.1	BC003731	Vdac2	26.045	33.533	58.091	1.288	2.230
1420630_at	Mm.34182.1	NM_028982	8430419L09Rik	4.616	5.188	10.284	1.124	2.228
1417912_at	Mm.28092.1	NM_025318	0610009E20Rik	5.367	7.217	11.955	1.345	2.227
1428288_at	Mm.183017.1	AW488885	2310051E17Rik	2.365	1.239	5.267	0.524	2.227
1438092_x_at	Mm.916.5	AV003424	H2afz	16.598	17.930	36.947	1.080	2.226
1434728_at	Mm.32184.1	BM220576	2900064I19Rik	0.879	0.609	1.957	0.693	2.226
1435534_a_at	Mm.6932.2	BF228097	Tomm20	5.321	7.170	11.844	1.347	2.226
1419038_a_at	Mm.23692.1	BB283759	Csnk2a1	4.250	5.761	9.459	1.356	2.226
1452011_a_at	Mm.12790.1	AA203925	Uxs1	1.132	1.751	2.517	1.547	2.224
1452053_a_at	Mm.110600.2	BB771960	1600019D15Rik	6.269	10.973	13.932	1.750	2.222
1436915_x_at	Mm.197518.2	AU024771	Laptm4b	8.891	16.429	19.753	1.848	2.222
1450968_at	Mm.181933.1	AK003966	Uqcrfs1	23.150	28.944	51.423	1.250	2.221

1436803_a_at	Mm.4537.2	AV161987	Ndufb9	22.265	32.857	49.428	1.476	2.220
1425568_a_at	Mm.110600.1	BC005562	1600019D15Rik	5.401	7.620	11.989	1.411	2.220
1434155_a_at	Mm.5624.2	BB174350	2310061I04Rik	4.881	6.432	10.834	1.318	2.220
1420058_s_at	Mm.198745.1	AI480750	2410166I05Rik	2.651	3.969	5.884	1.497	2.219
1448663_s_at	Mm.28146.1	NM_138656	Mvd	3.259	4.306	7.230	1.321	2.218
1424178_at	Mm.212927.1	BC014728	1110001E17Rik	0.943	1.558	2.092	1.652	2.218
1428333_at	Mm.27579.1	AK013740	6530401D17Rik	6.478	8.413	14.360	1.299	2.217
1416971_at	Mm.2151.1	NM_009945	Cox7a2	28.417	34.517	62.989	1.215	2.217
1418522_at	Mm.22508.1	NM_013604	Mtx1	3.202	3.979	7.098	1.243	2.216
1450997_at	Mm.25559.1	AV173139	Stk17b	0.594	0.484	1.316	0.814	2.216
1423087_a_at	Mm.177734.1	BB453951	1110002E23Rik	12.743	22.982	28.213	1.803	2.214
1416710_at	Mm.41489.1	NM_026239	9030603L14Rik	3.857	3.121	8.538	0.809	2.214
1427148_at	Mm.41711.2	BF160731	Pja2	1.598	2.857	3.538	1.788	2.214
1455006_at	Mm.5265.1	W42220	2310016M24Rik	7.661	13.377	16.957	1.746	2.214
1452190_at	Mm.39856.1	AK011112	2510048K03Rik	0.829	0.878	1.834	1.059	2.212
1415753_at	Mm.29802.1	BC005632	D10Bwg1364e	5.966	8.657	13.191	1.451	2.211
1433750_at	Mm.200342.1	AV287019	Slc31a1	2.970	3.721	6.562	1.253	2.210
1417582_s_at	Mm.23894.1	NM_020046	Dhodh	1.013	0.887	2.237	0.875	2.208
1430700_a_at	Mm.9277.2	AK005158	Pla2g7	13.913	11.344	30.712	0.815	2.207
1423478_at	Mm.4182.1	BF660388	Prkcb	1.730	0.592	3.818	0.342	2.207
1453920_a_at	Mm.27132.2	BB000068	Mospd2	0.878	1.301	1.937	1.482	2.206
1451627_a_at	Mm.2861.2	U75372	Slc1a2	0.535	0.366	1.180	0.684	2.206
1449575_a_at	Mm.426.1	NM_013541	Gstp1	16.968	31.335	37.422	1.847	2.205
1428421_a_at	Mm.38521.1	BI453663	2700085E05Rik	6.146	12.072	13.548	1.964	2.204
1422810_at	Mm.116637.1	BF780333	Zfp191	2.097	2.491	4.621	1.188	2.203
1418005_at	Mm.29141.1	BC013509	Sdhb	25.456	31.892	56.088	1.253	2.203
1433962_at	Mm.38041.1	BB131965	6720458F09Rik	0.727	1.092	1.601	1.502	2.203
1437683_x_at	Mm.200975.8	AV047585	Serf2	14.828	20.601	32.667	1.389	2.203
1416290_a_at	Mm.29582.1	NM_011874	Psmc4	3.877	5.390	8.540	1.390	2.203
1427915_s_at	Mm.176927.2	AI019214	Tceb1	1.857	3.050	4.089	1.642	2.201
1423654_a_at	Mm.21281.1	AV045658	Rnf4	5.803	8.929	12.774	1.539	2.201
1450459_at	Mm.218254.1	NM_023220	2010106G01Rik	1.994	2.833	4.389	1.420	2.201
1423112_at	Mm.24529.1	AK009276	Ube2d3	0.901	1.275	1.983	1.415	2.201
1448387_at	Mm.29405.1	NM_019712	Rbx1	19.742	34.168	43.440	1.731	2.200
1426123_a_at	Mm.13705.8	AF273691	Rrbp1	0.628	0.666	1.382	1.061	2.200
1440867_at	Mm.208557.1	BB703945	Spry4	1.497	0.746	3.294	0.498	2.200
1448198_a_at	Mm.2060.1	NM_026061	Ndufb8	26.388	36.533	58.057	1.384	2.200
1435564_at	Mm.133211.1	BB547893	C230078M08Rik	0.805	0.115	1.771	0.142	2.199
1448763_at	Mm.27123.1	NM_026487	Atad1	5.158	8.482	11.345	1.644	2.199
1438171_x_at	Mm.29122.4	BB056666	0610012D09Rik	14.742	20.681	32.419	1.403	2.199
1428428_at	Mm.25616.1	AK004244	Wbscr21	2.308	3.539	5.075	1.533	2.199
1422430_at	Mm.20315.1	NM_021891	Fignl1	2.332	0.616	5.126	0.264	2.198
1416501_at	Mm.10504.1	NM_011062	Pdpk1	0.599	1.029	1.317	1.717	2.197
1423911_at	Mm.205569.1	BC023062	Ppp2r5a	3.315	6.452	7.284	1.946	2.197
1449250_at	Mm.35089.1	NM_033573	Prcc	0.554	0.903	1.216	1.631	2.196
1449281_at	Mm.8074.1	NM_008738	Nrtn	0.471	0.975	1.035	2.069	2.196
1449913_at	Mm.4958.1	NM_009550	Zfp2	0.613	0.816	1.345	1.332	2.195
1460194_at	Mm.27066.1	NM_010726	Phyh	2.309	4.457	5.068	1.930	2.195
1436451_a_at	Mm.21507.3	AW824211	1110032D12Rik	23.227	44.668	50.965	1.923	2.194
1423957_at	Mm.34109.2	BI440638	2700083B06Rik	4.102	5.702	9.000	1.390	2.194
1455401_at	Mm.220852.1	AW061083	Camkk2	0.839	1.324	1.841	1.577	2.194
1420890_at	Mm.10001.1	BB129992	Hccs	3.299	4.495	7.236	1.363	2.193

1417073_a_at	Mm.2655.1	NM_021881	Qk	23.485	37.895	51.508	1.614	2.193
1448254_at	Mm.3063.1	BC002064	Ptn	31.869	33.805	69.860	1.061	2.192
1439570_at	Mm.100206.1	BE957162	LOC242915	7.803	5.952	17.097	0.763	2.191
1436848_x_at	Mm.183042.2	AV348702	Impa1	2.119	3.507	4.642	1.655	2.191
1451918_a_at	Mm.41892.2	BC023059	5830457J20Rik	1.688	2.947	3.698	1.746	2.191
1417606_a_at	Mm.1971.1	NM_007591	Calr	22.499	36.766	49.272	1.634	2.190
1421923_at	Mm.46048.1	BQ179335	Sh3bp5	0.956	1.292	2.094	1.351	2.190
1424283_at	Mm.13912.1	BC008139	Jtb	8.404	16.762	18.397	1.995	2.189
1428631_a_at	Mm.988.2	BG075002	Uqcrc2	18.355	24.701	40.179	1.346	2.189
1428949_at	Mm.37962.1	AK014235	Xpot	4.481	6.779	9.807	1.513	2.189
1425632_a_at	Mm.45131.1	BC019216	Pqlc2	0.756	1.273	1.654	1.685	2.188
1448237_x_at	Mm.9745.1	NM_008492	Ldh2	22.514	33.526	49.216	1.489	2.186
1450970_at	Mm.19039.1	AA792094	Got1	4.545	3.157	9.934	0.695	2.186
1416926_at	Mm.28708.1	AW495711	Trp53inp1	12.492	15.636	27.299	1.252	2.185
1422910_s_at	Mm.219634.1	AU022584	Smc6l1	0.991	1.036	2.167	1.045	2.185
1460376_a_at	Mm.213007.1	BC011509	Cox15	0.805	0.838	1.759	1.041	2.185
1438040_a_at	Mm.4526.3	BE995678	Tra1	10.873	13.843	23.752	1.273	2.185
1424114_s_at	Mm.148395.1	BG970109	Lamb1-1	0.602	0.133	1.314	0.222	2.184
1423232_at	Mm.5025.1	X63190	Etv4	2.599	0.022	5.674	0.008	2.183
1417320_at	Mm.21535.1	NM_024478	Grpel1	6.629	10.948	14.463	1.652	2.182
1416538_at	Mm.196345.1	NM_011734	Ysg2	0.745	1.210	1.625	1.625	2.181
1431829_a_at	Mm.110594.2	AK004876	Rgl3	0.769	1.218	1.677	1.584	2.181
1456241_a_at	Mm.193091.3	BB315716	1810073N04Rik	6.546	9.024	14.279	1.378	2.181
1426922_s_at	Mm.6461.1	BB130716	Hrb	1.038	1.950	2.264	1.879	2.181
1456355_s_at	Mm.172947.2	BE134381	Srr1	0.886	1.107	1.931	1.250	2.180
1424171_a_at	Mm.44240.1	BC004749	Hagh	1.899	2.765	4.137	1.456	2.178
1435974_at	Mm.44841.1	BB271482	Arhgef9	0.848	1.500	1.846	1.770	2.178
1452866_at	Mm.29192.1	AK013880	Nars	8.251	13.692	17.970	1.659	2.178
1455583_at	Mm.25687.1	AV371374	Gne	0.493	0.568	1.073	1.153	2.178
1448464_at	Mm.24353.1	NM_019661	0610042I15Rik	7.442	10.909	16.207	1.466	2.178
1452146_a_at	Mm.213007.2	BC021498	Cox15	1.052	1.047	2.291	0.995	2.177
1420608_at	Mm.205937.1	AV116216	Rbm18	3.523	6.354	7.668	1.804	2.177
1421019_at	Mm.24863.1	BC013506	1700021F05Rik	3.669	4.427	7.983	1.207	2.176
1448203_at	Mm.14663.1	NM_013795	Atp5l	19.801	26.618	43.082	1.344	2.176
1456736_x_at	Mm.181892.4	AV217938	5230400G24Rik	19.672	38.170	42.788	1.940	2.175
1423361_at	Mm.23335.1	BB826168	Yme1l1	2.070	2.917	4.501	1.409	2.174
1422578_at	Mm.43822.1	AB056479	Cs	26.050	30.832	56.636	1.184	2.174
1416021_a_at	Mm.741.1	BC002008	Fabp5	43.654	79.034	94.790	1.810	2.171
1437864_at	Mm.41916.1	BE632137	D6Ucla1e	0.887	1.324	1.925	1.493	2.171
1435635_at	Mm.21539.1	BB549335	A030012M09Rik	0.654	0.923	1.420	1.412	2.171
1452150_at	Mm.206206.1	BG071197	AU040320	4.553	7.975	9.882	1.752	2.171
1431225_at	Mm.41702.2	BB656631	Sox11	6.240	6.214	13.540	0.996	2.170
1430811_a_at	Mm.151315.2	AK010351	Cdca1	3.023	0.867	6.558	0.287	2.169
1425179_at	Mm.3379.2	AF237702	Shmt1	0.855	0.214	1.855	0.250	2.169
1450925_a_at	Mm.30120.1	BB836796	Rps27l	24.469	30.671	53.041	1.253	2.168
1451448_a_at	Mm.7686.1	BC026936	1110005A03Rik	2.168	3.039	4.699	1.401	2.167
1451411_at	Mm.103439.1	BC020004	Gprc5b	2.358	4.265	5.108	1.809	2.166
1425528_at	Mm.3869.2	L06502	Prrx1	0.865	1.362	1.872	1.575	2.166
1433954_at	Mm.220992.2	AV227569	4632419I22Rik	3.534	4.138	7.653	1.171	2.165
1438686_at	Mm.3428.3	BB531220	Eif4g1	0.712	1.172	1.541	1.647	2.165
1421012_at	Mm.7588.1	NM_009275	Srprb	1.156	1.246	2.500	1.078	2.163
1436684_a_at	Mm.34105.2	AV066689	Riok2	0.924	1.572	1.997	1.702	2.163

1439455_x_at	Mm.19142.4	AV267494	Capza1	6.830	10.237	14.771	1.499	2.163
1455955_s_at	Mm.6118.2	BB041005	Snx17	34.396	63.954	74.383	1.859	2.163
1417100_at	Mm.11175.1	AV336016	425O18-1	1.755	1.760	3.796	1.002	2.162
1439448_x_at	Mm.45233.3	AV007149	2400003B06Rik	8.044	11.482	17.390	1.427	2.162
1416969_at	Mm.20858.1	NM_013882	Gtse1	0.892	0.374	1.929	0.419	2.161
1442038_at	Mm.45704.1	AV134514	1700009P03Rik	1.346	1.996	2.908	1.483	2.161
1452167_at	Mm.28026.1	BC019936	2810407C02Rik	19.130	31.811	41.314	1.663	2.160
1421754_at	MmAffx.1.3	NM_133243	AY036118	6.247	8.360	13.491	1.338	2.160
1415889_a_at	Mm.4526.1	NM_011631	Tra1	10.596	13.358	22.880	1.261	2.159
1432164_a_at	Mm.141945.3	AK003189	5730591C18Rik	11.855	22.558	25.594	1.903	2.159
1416499_a_at	Mm.90496.1	NM_011722	Dctn6	5.561	10.664	12.002	1.918	2.158
1426895_at	Mm.153618.1	BB579760	Zfp191	7.513	9.208	16.203	1.226	2.157
1450974_at	Mm.36851.1	BI788452	Timp4	4.335	0.236	9.349	0.054	2.157
1448958_at	Mm.21761.1	NM_022329	Ifrg15	1.904	2.438	4.105	1.281	2.156
1417433_at	Mm.34302.1	NM_011942	Lypla2	10.587	16.889	22.827	1.595	2.156
1420920_a_at	Mm.6836.1	NM_007476	Arf1	13.442	19.441	28.976	1.446	2.156
1424216_a_at	Mm.3170.1	U52197	Papola	3.435	4.330	7.403	1.260	2.155
1416067_at	Mm.168.1	NM_013562	Ifrd1	3.629	4.799	7.819	1.323	2.155
1449168_a_at	Mm.7622.1	BC003735	Akap2	0.719	0.922	1.549	1.282	2.154
1429619_a_at	Mm.28233.2	BB040330	8430406I07Rik	0.713	0.724	1.535	1.016	2.154
1429428_at	Mm.4269.4	BB175494	Tcf7l2	1.216	2.239	2.618	1.842	2.154
1441960_x_at	Mm.54092.1	AV267590	5730494M16Rik	4.894	6.554	10.537	1.339	2.153
1438992_x_at	Mm.641.4	AV314773	Atf4	2.654	3.081	5.714	1.161	2.153
1455126_x_at	Mm.29828.3	AV209841	2310028O11Rik	23.471	30.791	50.528	1.312	2.153
1422769_at	Mm.196461.1	BG920261	Syncrip	3.594	4.193	7.736	1.167	2.153
1454666_at	Mm.30693.1	AV230488	Klf3	7.909	8.539	17.025	1.080	2.153
1452638_s_at	Mm.221051.1	BC027538	Dnm1l	0.540	0.786	1.161	1.456	2.152
1416346_at	Mm.30034.1	W82151	Timm8a	2.308	2.103	4.966	0.911	2.152
1456012_x_at	Mm.181237.2	AV101824	Rnaset2	5.617	9.284	12.084	1.653	2.151
1453366_at	Mm.41510.1	AK012596	Tdrkh	1.504	1.892	3.235	1.258	2.151
1449003_a_at	Mm.6577.1	NM_016800	Vti1b	10.269	20.386	22.075	1.985	2.150
1433566_at	Mm.80123.1	BB381618	B230331P10Rik	1.346	0.678	2.893	0.504	2.149
1427929_a_at	Mm.206159.1	BG063905	2310036D04Rik	3.048	3.453	6.549	1.133	2.149
1433804_at	Mm.28598.1	BQ032637	Jak1	0.748	1.449	1.606	1.937	2.148
1416470_a_at	Mm.22333.1	NM_133933	Rpn1	8.531	12.144	18.314	1.423	2.147
1418356_at	Mm.17498.1	NM_138670	Mpst	2.066	3.457	4.432	1.674	2.145
1416058_s_at	Mm.12677.1	NM_020615	Atp5c1	40.026	52.504	85.873	1.312	2.145
1448543_at	Mm.182294.1	NM_025531	2310042G06Rik	2.446	3.017	5.243	1.233	2.143
1423079_a_at	Mm.6932.1	AK002902	Tomm20	25.951	37.085	55.590	1.429	2.142
1451248_at	Mm.153872.1	BC006705	BC006705	2.910	3.410	6.232	1.172	2.142
1460010_a_at	Mm.3871.5	BB490580	Ptdss2	2.612	5.066	5.593	1.939	2.141
1447754_x_at	Mm.119502.1	BB130418	Thap4	1.649	2.301	3.531	1.395	2.141
1416429_a_at	Mm.4215.1	NM_009804	Cat	10.508	19.733	22.494	1.878	2.141
1448699_at	Mm.43397.1	NM_025317	Mrpl54	1.771	2.434	3.790	1.374	2.140
1434993_at	Mm.100160.1	BM942851	B830045N13Rik	1.625	1.441	3.476	0.887	2.140
1423873_at	Mm.30198.1	BC021460	Lsm1	3.125	4.353	6.686	1.393	2.140
1431054_at	Mm.28694.1	AK019126	Lsm6	5.896	4.814	12.614	0.817	2.140
1423423_at	Mm.709.1	BF319868	Grp58	18.871	31.433	40.356	1.666	2.139
1437462_x_at	Mm.114768.1	BB484002	Mmp15	5.714	2.923	12.215	0.512	2.138
1417418_s_at	Mm.43415.1	NM_007748	Cox6a1	35.981	56.152	76.902	1.561	2.137
1420638_at	Mm.44199.1	BC024942	Prps2	0.976	1.236	2.086	1.266	2.137
1423137_at	Mm.27348.1	BG073338	Rala	5.558	9.303	11.873	1.674	2.136

1441315_s_at	Mm.198170.1	BB449198	Slc19a2	0.559	0.464	1.194	0.830	2.136
1435086_s_at	Mm.28047.2	AV298107	Klhdc2	8.526	14.657	18.209	1.719	2.136
1460638_at	Mm.145488.1	NM_134151	Yars	2.883	3.856	6.156	1.337	2.135
1417259_a_at	Mm.2945.1	NM_009798	Capzb	8.580	15.181	18.323	1.769	2.135
1421846_at	Mm.28489.1	BM730566	Wsb2	3.752	4.964	8.012	1.323	2.135
1437284_at	Mm.200772.1	BB259670	Fzd1	2.741	4.192	5.851	1.529	2.134
1452938_at	Mm.45416.1	BQ174247	C030032C09Rik	0.669	0.422	1.428	0.631	2.134
1433737_at	Mm.28370.1	BQ175168	4732477C12Rik	2.667	4.612	5.693	1.729	2.134
1425733_a_at	Mm.2012.2	BC016890	Eps8	1.599	1.262	3.411	0.789	2.134
1427100_at	Mm.41925.1	BE947704	1810034B16Rik	18.848	22.422	40.213	1.190	2.134
1434890_at	Mm.203884.1	BG072813	Opa1	5.700	10.424	12.161	1.829	2.133
1444723_at	Mm.212591.1	BB049759	6530418L21Rik	1.399	0.137	2.985	0.098	2.133
1453008_at	Mm.29227.1	AK009004	2300002D11Rik	0.948	1.462	2.022	1.542	2.133
1423152_at	Mm.38800.1	BF303544	Vapb	4.069	7.832	8.679	1.925	2.133
1416104_at	Mm.89579.1	NM_011900	Mpdu1	4.612	7.185	9.834	1.558	2.132
1426395_s_at	Mm.27695.1	BB379268	Eif3s1	1.097	1.569	2.338	1.431	2.132
1421877_at	Mm.68933.1	BC024514	Mapk9	1.228	1.986	2.618	1.618	2.132
1455992_at	Mm.34548.2	BG967636	BC048841	2.350	3.399	5.008	1.447	2.131
1418706_at	Mm.30058.1	NM_023805	Slc38a3	6.393	2.888	13.621	0.452	2.131
1415988_at	Mm.30012.1	BG065877	Hdlbp	1.213	1.326	2.584	1.093	2.130
1429650_at	Mm.22193.1	AV091811	2310004N11Rik	1.501	2.311	3.197	1.539	2.130
1451308_at	Mm.83949.1	BB829575	Elovl4	1.282	1.386	2.730	1.081	2.129
1417744_a_at	Mm.27832.1	BC006907	Ralb	3.047	4.761	6.484	1.562	2.128
1430134_a_at	Mm.37486.1	AK008774	2210023C10Rik	0.842	0.867	1.792	1.030	2.128
1451700_a_at	Mm.46533.1	BC019557	1110007L15Rik	3.114	4.658	6.623	1.496	2.127
1456599_at	Mm.41899.1	BB745947	6330587F24Rik	2.201	3.065	4.682	1.392	2.127
1418326_at	Mm.27943.1	BC026131	Slc7a5	3.135	1.905	6.667	0.608	2.127
1433548_at	Mm.181216.2	BB137740	Mare	1.427	2.128	3.033	1.491	2.126
1460419_a_at	Mm.4182.2	X59274	Prkcb	9.023	3.078	19.184	0.341	2.126
1452184_at	Mm.4537.1	W29413	Ndufb9	25.402	39.997	53.994	1.575	2.126
1418822_a_at	Mm.196526.1	BI248938	Arf6	7.409	12.221	15.742	1.649	2.125
1434637_x_at	Mm.2137.4	BF017589	Sin3b	14.635	23.858	31.087	1.630	2.124
1456227_x_at	Mm.1603.4	AV310432	Rbbp7	15.098	23.180	32.059	1.535	2.123
1416603_at	Mm.13917.1	NM_009079	Rpl22	23.288	24.848	49.437	1.067	2.123
1419616_at	Mm.7106.1	NM_007561	Bmpr2	0.714	1.006	1.515	1.409	2.123
1428153_at	Mm.27863.1	AK004151	Mrps10	2.797	3.948	5.937	1.412	2.122
1423616_at	Mm.176.1	BE373492	Tarbp2	1.322	1.806	2.804	1.367	2.121
1419385_a_at	Mm.182053.1	NM_026842	Ubqln1	3.023	3.716	6.412	1.229	2.121
1415870_at	Mm.7515.1	NM_007594	Calu	11.298	19.274	23.961	1.706	2.121
1455951_at	Mm.28173.1	BM211104	Mars	3.662	4.498	7.765	1.228	2.120
1452782_a_at	Mm.3533.2	AK002358	Txn2	3.606	4.059	7.646	1.126	2.120
1455102_at	Mm.164450.1	BB213860	D330037H05Rik	1.280	1.758	2.714	1.373	2.120
1426965_at	Mm.30543.1	BC025198	5830461H18Rik	7.604	12.456	16.120	1.638	2.120
1449533_at	Mm.103393.1	NM_026433	1810057C19Rik	0.690	0.343	1.463	0.497	2.119
1437347_at	Mm.129235.1	BF100813	Ednrb	33.405	39.804	70.767	1.192	2.118
1433572_a_at	Mm.25018.2	AA673192	BC010304	17.382	22.077	36.819	1.270	2.118
1417684_at	Mm.781.1	NM_008188	Gt(ROSA)26asSor	2.794	4.778	5.917	1.710	2.118
1443733_x_at	Mm.207227.1	C85233	Pold3	0.773	0.980	1.636	1.268	2.117
1452043_at	Mm.19031.1	AK020401	2310011J03Rik	2.151	3.826	4.554	1.779	2.117
1443558_s_at	Mm.218238.1	AU040402	C630002B14Rik	1.153	1.689	2.441	1.465	2.117
1448970_at	Mm.23896.1	AV088109	1200007B05Rik	4.152	7.544	8.786	1.817	2.116
1437442_at	Mm.25535.1	BG067986	Pcdh7	1.656	1.471	3.504	0.888	2.116

1459842_x_at	Mm.109197.1	AV035368	Nubp2	0.777	1.336	1.644	1.719	2.115
1452681_at	Mm.3856.2	AK009220	Dtymk	2.514	2.702	5.317	1.075	2.115
1448901_at	Mm.22224.1	NM_019696	Cpxm1	1.773	2.495	3.748	1.408	2.114
1428273_at	Mm.142570.1	BE573480	1110065L07Rik	0.999	1.655	2.111	1.657	2.113
1428214_at	Mm.27901.1	BB609428	Tomm7	8.927	13.572	18.863	1.520	2.113
1425227_a_at	Mm.20869.2	BC001995	Atp6v0a1	0.956	1.113	2.020	1.165	2.113
1422462_at	Mm.6476.1	NM_026024	2700084L22Rik	1.387	0.343	2.931	0.247	2.113
1418084_at	Mm.27448.1	AK011144	Nrp	0.773	0.667	1.633	0.863	2.113
1448722_s_at	Mm.27245.1	NM_026494	6330579B17Rik	0.613	1.107	1.295	1.806	2.113
1428061_at	Mm.28421.1	AK014330	Hat1	3.434	3.016	7.252	0.878	2.112
1421933_at	Mm.28003.1	NM_007626	Cbx5	2.542	2.663	5.368	1.048	2.112
1416780_at	Mm.26550.1	NM_021514	Pfkm	7.067	11.990	14.921	1.697	2.112
1448971_at	Mm.29063.1	NM_025556	2410022L05Rik	1.309	1.406	2.764	1.074	2.111
1419034_at	Mm.23692.1	BB283759	Csnk2a1	2.048	2.438	4.324	1.190	2.111
1455829_at	Mm.219648.2	BG066490	Thoc1	1.692	2.287	3.572	1.351	2.111
1449615_s_at	Mm.215181.1	C77256	Hdlbp	1.032	1.393	2.177	1.351	2.110
1448531_at	Mm.7362.1	NM_010722	Lmnb2	0.538	0.562	1.136	1.045	2.110
1420013_s_at	Mm.195350.1	C77434	Lss	3.457	3.695	7.294	1.069	2.110
1428630_x_at	Mm.29230.2	AK021220	Haghl	3.431	6.038	7.238	1.760	2.110
1452741_s_at	Mm.41718.1	BQ175968	Gpd2	0.589	0.436	1.242	0.741	2.110
1423850_at	Mm.205855.1	BC013625	D13Wsu123e	3.313	4.310	6.988	1.301	2.109
1425459_at	Mm.24298.1	BB197262	Mtmr2	2.201	3.289	4.643	1.494	2.109
1424408_at	Mm.24551.1	BC010816	Lims2	2.198	1.639	4.634	0.746	2.108
1415679_at	Mm.41511.1	NM_025498	1700023M09Rik	15.775	27.664	33.243	1.754	2.107
1450275_x_at	Mm.44214.1	NM_026454	2510010F15Rik	3.679	5.002	7.753	1.360	2.107
1447090_s_at	Mm.200679.1	AU019749	Arl1	10.548	17.009	22.221	1.612	2.107
1443836_x_at	Mm.104789.1	BB064885	8430408H12Rik	2.047	3.464	4.308	1.693	2.105
1418316_a_at	Mm.28678.1	BC026445	Mark3	6.523	11.850	13.723	1.817	2.104
1441917_s_at	Mm.200575.1	BB468188	9030407H20Rik	0.688	0.548	1.446	0.797	2.104
1452597_at	Mm.4011.1	AW045976	1110046L09Rik	3.547	3.071	7.456	0.866	2.102
1427314_at	Mm.24086.1	AV225029	3930401E15Rik	8.356	14.506	17.551	1.736	2.100
1424554_at	Mm.18838.1	BC025479	6330548N22Rik	4.929	8.216	10.353	1.667	2.100
1423716_s_at	Mm.22514.1	BC008273	Atp5d	20.118	23.576	42.247	1.172	2.100
1426586_at	Mm.28466.1	AI647500	Slc25a11	6.904	8.923	14.493	1.292	2.099
1431196_at	Mm.153302.1	BG296252	Atp2c1	0.918	1.285	1.926	1.400	2.099
1430640_a_at	Mm.25594.1	BI695530	Prkar2b	0.795	0.732	1.669	0.921	2.099
1429776_a_at	Mm.2701.2	AK005680	Dnajb6	2.364	3.870	4.959	1.637	2.098
1420494_x_at	Mm.331.1	BC006680	Ubc	27.770	53.190	58.222	1.915	2.097
1433685_a_at	Mm.22226.2	BM248225	6430706D22Rik	4.496	3.023	9.423	0.672	2.096
1436494_x_at	Mm.195313.2	BF319098	D8Ertd812e	1.574	2.450	3.299	1.556	2.095
1428936_at	Mm.103450.1	BI080417	2810442I22Rik	1.955	3.523	4.095	1.803	2.095
1449553_at	Mm.46513.1	AK009389	2610200G18Rik	6.411	7.231	13.428	1.128	2.095
1417098_s_at	Mm.29096.1	NM_025297	Nrbf1	1.190	1.207	2.492	1.014	2.095
1436908_at	Mm.28192.3	BG076129	Pcm1	2.686	4.762	5.624	1.773	2.094
1429038_at	Mm.29901.1	BI904336	1500034J01Rik	2.776	4.263	5.809	1.536	2.093
1423446_at	Mm.10294.1	AI642212	Dapk3	1.547	2.319	3.232	1.500	2.090
1418695_a_at	Mm.29194.1	BG071725	Kcmf1	7.589	12.696	15.860	1.673	2.090
1433481_at	Mm.64032.1	BB027759	BC029109	1.212	2.358	2.532	1.946	2.090
1450112_a_at	Mm.25.1	NM_008087	Gas2	0.788	0.665	1.647	0.844	2.090
1429958_x_at	Mm.29230.3	BI790001	Haghl	3.821	6.387	7.983	1.672	2.089
1417725_a_at	Mm.27657.1	BC021593	Sssca1	0.723	0.830	1.510	1.149	2.089
1415719_s_at	Mm.196465.1	BE134617	Arcp	5.337	9.017	11.150	1.690	2.089

1436405_at	Mm.26388.1	BG068753	Dock4	0.775	0.628	1.620	0.809	2.089
1437995_x_at	Mm.141945.7	AV219419	38967	8.155	10.189	17.036	1.249	2.089
1422731_at	Mm.10987.1	AK004806	Limd1	3.202	4.097	6.688	1.280	2.089
1422823_at	Mm.2012.1	NM_007945	Eps8	3.376	3.650	7.052	1.081	2.089
1428068_at	Mm.29651.1	AK003990	1110030L07Rik	7.167	9.547	14.966	1.332	2.088
1448969_at	Mm.38894.1	NM_026510	2310037B18Rik	0.758	0.840	1.582	1.109	2.088
1451572_a_at	Mm.181892.1	BC016597	5230400G24Rik	6.003	11.649	12.530	1.940	2.087
1451077_at	Mm.4419.1	BM114165	Rpl5	4.079	6.002	8.508	1.472	2.086
1448226_at	Mm.99.1	NM_009104	Rrm2	8.014	2.067	16.714	0.258	2.085
1430713_s_at	Mm.9326.4	AI429575	Grim19	26.293	38.475	54.830	1.463	2.085
1426853_at	Mm.28805.2	BM229104	Set	15.209	20.795	31.713	1.367	2.085
1425460_at	Mm.24298.1	BB197262	Mtmr2	3.192	4.197	6.655	1.315	2.085
1426830_a_at	Mm.27733.1	BB831090	Ahcyl1	15.794	19.684	32.928	1.246	2.085
1426435_at	Mm.34049.1	AV225967	2810439K08Rik	2.069	3.387	4.312	1.637	2.084
1416591_at	Mm.28941.1	AF327929	Rab34	4.007	6.175	8.352	1.541	2.084
1437280_s_at	Mm.18700.2	AV110762	1200009K13Rik	22.925	29.742	47.780	1.297	2.084
1448391_at	Mm.25306.1	NM_019773	Rab9	11.116	17.929	23.166	1.613	2.084
1424427_at	Mm.24671.1	BC027337	D1Ertd251e	3.016	3.857	6.285	1.279	2.084
1424177_at	Mm.212927.1	BC014728	1110001E17Rik	1.239	2.101	2.579	1.696	2.082
1437071_at	Mm.29969.1	BB471576	Eif1ay	1.185	2.202	2.465	1.859	2.080
1428306_at	Mm.21697.1	AK017926	Ddit4	13.985	17.668	29.087	1.263	2.080
1416250_at	Mm.903.1	NM_007570	Btg2	5.943	6.993	12.360	1.177	2.080
1421155_at	Mm.87681.1	NM_080445	B3galt6	0.932	1.182	1.939	1.268	2.080
1451242_a_at	Mm.3294.1	BC003744	Ppp5c	3.090	4.848	6.425	1.569	2.079
1426306_a_at	Mm.22575.2	AF319976	Maged2	4.004	5.022	8.324	1.254	2.079
1416146_at	Mm.1032.1	BE912771	Hspa4	7.233	9.024	15.033	1.248	2.078
1415873_a_at	Mm.3118.1	NM_016860	Actr1a	13.297	23.399	27.614	1.760	2.077
1448882_at	Mm.28092.1	NM_025318	0610009E20Rik	5.850	9.200	12.148	1.573	2.077
1452883_a_at	Mm.196252.1	BQ176330	2310002J21Rik	5.309	7.512	11.012	1.415	2.074
1451541_at	Mm.98228.1	BC019781	Bcs1l	1.883	2.182	3.907	1.158	2.074
1434120_a_at	Mm.19170.2	AW742814	Metap2	13.777	20.949	28.572	1.521	2.074
1456109_a_at	Mm.25143.2	BB314055	Mrps15	2.943	3.526	6.102	1.198	2.074
1451145_s_at	Mm.28312.1	BC004641	0610039A15Rik	10.818	18.832	22.422	1.741	2.073
1437325_x_at	Mm.29751.3	BB251523	Pycs	3.816	4.371	7.910	1.145	2.073
1448665_at	Mm.742.1	NM_007868	Dmd	3.012	4.653	6.243	1.545	2.073
1434593_at	Mm.31867.1	AV271901	Eif5a2	1.194	1.717	2.475	1.437	2.072
1439012_a_at	Mm.3446.3	BB030204	Dck	6.413	4.819	13.290	0.751	2.072
1423195_at	Mm.3792.1	BM208582	Hiat1	3.016	5.063	6.247	1.679	2.072
1433539_at	Mm.14486.1	BB230296	Commd3	9.240	17.039	19.131	1.844	2.070
1428643_at	Mm.38399.1	AK015544	Mgat5	1.221	2.099	2.528	1.719	2.070
1453768_a_at	Mm.18228.2	AK005776	5430432M24Rik	1.005	0.685	2.080	0.681	2.070
1428025_s_at	Mm.38610.1	BC028271	1110020B03Rik	1.308	1.178	2.708	0.900	2.070
1448720_at	Mm.24737.1	NM_024194	2610040E16Rik	3.986	4.865	8.248	1.221	2.069
1425323_a_at	Mm.26783.1	BC008155	BC008155	7.103	8.871	14.699	1.249	2.069
1447849_s_at	Mm.180082.1	AV323441	Maf	0.730	1.293	1.510	1.772	2.069
1437497_a_at	Mm.1843.2	C77384	Hspca	15.784	26.118	32.654	1.655	2.069
1450747_at	Mm.151790.1	AW764104	Keap1	1.236	2.231	2.556	1.805	2.068
1433646_at	Mm.30208.1	BB667079	Mrps27	1.449	1.206	2.997	0.832	2.068
1436244_a_at	Mm.38608.2	AU067681	Tle2	0.567	0.265	1.172	0.467	2.067
1419664_at	Mm.19015.1	BC011164	Srr	0.813	1.041	1.680	1.280	2.066
1418986_a_at	Mm.34779.1	NM_013840	Uxt	1.559	1.739	3.221	1.115	2.066
1423399_a_at	Mm.4714.1	BG143658	Yaf2	6.793	9.566	14.031	1.408	2.066

1417369_at	Mm.3195.1	NM_008292	Hsd17b4	10.843	20.256	22.389	1.868	2.065
1417938_at	Mm.8156.1	BC003738	Rad51ap1	1.420	0.302	2.932	0.213	2.065
1436816_at	Mm.68427.2	BB559624	Nup133	2.047	1.516	4.227	0.740	2.065
1451312_at	Mm.28712.1	BC013503	Ndufs7	14.587	16.758	30.113	1.149	2.064
1459783_s_at	Mm.77024.1	AV218165	Cno	2.054	3.480	4.240	1.694	2.064
1437363_at	Mm.133785.1	BQ043238	Homer1	1.927	2.155	3.974	1.118	2.062
1448268_at	Mm.45233.1	NM_026211	2400003B06Rik	10.129	17.358	20.881	1.714	2.062
1429681_a_at	Mm.142511.2	AK020708	Gpsn2	28.900	56.530	59.578	1.956	2.062
1436113_a_at	Mm.196525.2	BB770696	St13	13.859	23.239	28.563	1.677	2.061
1416877_a_at	Mm.42826.1	AI594880	Mrpl51	12.143	16.831	25.026	1.386	2.061
1451299_at	Mm.106185.1	BC006875	Prkx	0.850	1.476	1.752	1.736	2.060
1424211_at	Mm.41877.1	BC011293	5730438N18Rik	1.398	2.554	2.880	1.827	2.060
1454857_at	Mm.29532.1	AW551457	1110063C11Rik	3.835	3.506	7.898	0.914	2.060
1437985_a_at	Mm.5624.3	BB379386	2310061I04Rik	3.759	4.601	7.739	1.224	2.059
1425045_at	Mm.41467.1	BC016255	Pla2g4b	1.938	2.092	3.990	1.079	2.058
1449044_at	Mm.36683.1	NM_025380	Eef1e1	6.960	9.425	14.325	1.354	2.058
1439413_x_at	Mm.27218.2	AV094989	Morf4l2	23.529	42.692	48.424	1.814	2.058
1422844_a_at	Mm.28975.1	NM_027432	2610312E17Rik	5.198	9.122	10.689	1.755	2.056
1439030_at	Mm.22554.2	BI410722	Gmppb	0.667	1.093	1.372	1.637	2.056
1421327_at	Mm.413.1	BM937510	Mtap2	2.553	2.539	5.250	0.994	2.056
1416024_x_at	Mm.3576.1	NM_009836	Cct3	21.416	32.427	44.038	1.514	2.056
1453689_at	Mm.27802.1	AK013325	Fance	0.803	0.738	1.652	0.919	2.056
1416479_a_at	Mm.30005.1	NM_025387	Tmem14c	9.920	15.209	20.399	1.533	2.056
1450667_a_at	Mm.43822.1	AB056479	Cs	29.641	31.618	60.946	1.067	2.056
1421866_at	Mm.4918.1	NM_008173	Nr3c1	0.520	0.755	1.070	1.451	2.056
1460387_a_at	Mm.196345.2	AF156856	Ysg2	1.107	1.915	2.275	1.730	2.056
1417699_at	Mm.24632.1	AV325174	2810405L04Rik	2.387	3.147	4.904	1.319	2.055
1417037_at	Mm.29709.1	NM_019716	Orc6l	2.843	2.623	5.841	0.923	2.055
1435112_a_at	Mm.182042.2	AV154755	Atp5h	27.441	39.229	56.382	1.430	2.055
1423752_at	Mm.27649.1	BC006843	Ddx47	3.897	5.587	8.005	1.434	2.054
1460563_at	Mm.26537.1	BE949414	D10Ertd438e	2.847	4.426	5.847	1.555	2.054
1439439_x_at	Mm.21086.4	AV104363	Eef1d	6.075	6.654	12.473	1.095	2.053
1418898_at	Mm.218854.1	BB704337	Lin7c	0.900	1.305	1.848	1.449	2.052
1427997_at	Mm.28983.1	BG064890	1110007M04Rik	3.242	3.072	6.653	0.947	2.052
1419866_s_at	Mm.199057.1	AW544490	Sca2	3.459	6.775	7.099	1.959	2.052
1434868_at	Mm.76653.2	AV282706	4.93343E+26	0.535	0.439	1.098	0.820	2.051
1426618_a_at	Mm.196045.1	AW212741	4930467B06Rik	3.408	4.979	6.991	1.461	2.051
1452587_at	Mm.30108.1	AK014859	Actr2	14.480	26.352	29.699	1.820	2.051
1418035_a_at	Mm.27705.1	NM_008922	Prim2	1.098	1.131	2.252	1.030	2.051
1417384_at	Mm.10211.1	NM_007647	Entpd5	1.059	1.685	2.172	1.591	2.051
1418819_at	Mm.175302.1	NM_026011	Arl10c	2.737	4.782	5.612	1.747	2.051
1451314_a_at	Mm.1021.2	L08431	Vcam1	1.190	1.507	2.440	1.266	2.050
1421830_at	Mm.42040.1	NM_009647	Ak4	1.443	0.944	2.959	0.654	2.050
1416198_at	Mm.182439.1	NM_020580	Th1l	3.515	5.291	7.200	1.505	2.048
1448422_at	Mm.30103.1	BB463345	1110014L17Rik	9.887	15.641	20.248	1.582	2.048
1420646_at	Mm.196035.1	NM_030250	D10Ertd438e	1.022	1.383	2.092	1.353	2.047
1426745_at	Mm.29852.1	BI156989	D11Bwg0434e	5.408	10.365	11.069	1.917	2.047
1416241_at	Mm.29296.1	NM_024206	Sec13l1	7.044	9.480	14.418	1.346	2.047
1456195_x_at	Mm.6424.5	BB543979	Itgb5	3.257	2.680	6.667	0.823	2.047
1416183_a_at	Mm.9745.1	NM_008492	Ldh2	23.717	31.833	48.533	1.342	2.046
1416567_s_at	Mm.20841.1	NM_025983	Atp5e	18.179	20.336	37.200	1.119	2.046
1457681_at	Mm.130790.1	BB532566	2610301F02Rik	0.521	0.393	1.065	0.754	2.046

1435325_at	Mm.34540.1	AV300436	2410018I08Rik	1.423	1.859	2.911	1.306	2.046
1459657_s_at	Mm.200445.1	AU018636	Rpo1-3	10.072	14.541	20.601	1.444	2.045
1436584_at	Mm.89982.2	BB529691	Spry2	9.268	6.284	18.954	0.678	2.045
1448440_x_at	Mm.29925.1	NM_080837	D17Wsu104e	0.983	1.374	2.009	1.398	2.045
1417575_at	Mm.46150.1	AK006346	4930586I02Rik	0.810	1.112	1.657	1.372	2.045
1450908_at	Mm.3941.1	BB406487	Eif4e	12.539	15.661	25.635	1.249	2.044
1429859_a_at	Mm.139700.2	AK006418	Arl2bp	11.200	18.454	22.898	1.648	2.044
1451149_at	Mm.22403.1	BC008527	Pgm2	3.610	5.296	7.380	1.467	2.044
1456741_s_at	Mm.15571.8	BB348674	Gpm6a	32.139	56.289	65.693	1.751	2.044
1431997_at	Mm.160318.1	AK013857	LOC14433	3.396	3.267	6.941	0.962	2.044
1418038_s_at	Mm.35774.1	NM_024438	Dusp19	1.445	1.664	2.951	1.152	2.042
1454714_x_at	Mm.16898.2	AA561726	Phgdh	18.484	26.553	37.747	1.437	2.042
1455129_at	Mm.183022.1	AV083741	2610103J23Rik	4.082	6.160	8.334	1.509	2.042
1448100_at	Mm.24593.1	NM_133797	4833439L19Rik	18.503	34.436	37.778	1.861	2.042
1439478_at	Mm.45431.1	BB500039	Mte1	2.284	4.515	4.663	1.977	2.042
1437336_x_at	Mm.177734.5	AV051733	1110002E23Rik	21.671	34.865	44.227	1.609	2.041
1428075_at	Mm.29065.1	BG968046	Ndufb4	17.760	21.264	36.241	1.197	2.041
1452940_x_at	Mm.38610.2	AI595920	1110020B03Rik	0.942	0.818	1.922	0.869	2.040
1453367_a_at	Mm.23572.2	BB178770	6330583M11Rik	3.535	4.572	7.213	1.293	2.040
1425348_a_at	Mm.7588.2	BC003798	Srprb	2.189	3.420	4.466	1.563	2.040
1456086_x_at	Mm.14616.2	BB026366	Pqbp1	8.805	13.124	17.957	1.490	2.039
1417331_a_at	Mm.7760.1	NM_019665	Arl6	2.481	4.644	5.057	1.872	2.039
1428410_at	Mm.28064.1	AK013287	Mak3	2.401	2.635	4.894	1.098	2.038
1433978_at	Mm.200822.1	BG073646	4930430F08Rik	1.356	1.279	2.763	0.943	2.038
1416190_a_at	Mm.28375.1	BC003707	Sec61a1	1.691	2.616	3.446	1.547	2.037
1460652_at	Mm.2563.1	NM_007953	Esrra	1.895	2.272	3.861	1.199	2.037
1438230_at	Mm.30613.1	BM240147	AI551093	1.037	1.916	2.112	1.848	2.037
1436339_at	Mm.53.1	BE627374	1810058I24Rik	5.470	10.311	11.139	1.885	2.036
1452095_a_at	Mm.22362.2	AK005204	H47	0.863	1.385	1.758	1.604	2.036
1456133_x_at	Mm.6424.4	BB543646	Itgb5	8.734	8.505	17.782	0.974	2.036
1428520_at	Mm.25322.1	AK004019	1110032A13Rik	2.797	4.500	5.694	1.609	2.036
1419369_at	Mm.42154.1	NM_019706	Rnf138	0.987	1.564	2.008	1.585	2.034
1452346_at	Mm.29628.1	AV032053	1500032M01Rik	4.338	7.475	8.820	1.723	2.033
1438938_x_at	Mm.36241.3	AV212294	Bcap37	8.380	8.680	17.035	1.036	2.033
1423785_at	Mm.140619.1	BE995700	Egln1	5.445	6.969	11.068	1.280	2.033
1448903_at	Mm.29812.1	NM_053102	38975	19.946	34.254	40.544	1.717	2.033
1428440_at	Mm.30928.1	AK019150	Slc25a12	5.853	6.880	11.897	1.176	2.033
1451419_at	Mm.33268.1	BC023083	Ssb4	2.955	0.778	6.006	0.263	2.032
1460260_s_at	Mm.6952.1	U20619	Kpna1	4.797	6.448	9.744	1.344	2.031
1422480_at	Mm.7356.1	NM_017472	Snx3	25.343	41.096	51.482	1.622	2.031
1448337_at	Mm.17392.1	NM_018888	2410003P15Rik	1.858	2.115	3.774	1.138	2.031
1460221_at	Mm.22421.1	BC003708	Tebp	18.311	30.082	37.173	1.643	2.030
1435995_at	Mm.13859.3	BF018217	Mrpl22	7.956	12.007	16.151	1.509	2.030
1415672_at	Mm.17035.1	NM_020585	Golga7	15.952	26.427	32.376	1.657	2.030
1416482_at	Mm.3679.1	BB833716	Ttc3	2.309	2.189	4.685	0.948	2.030
1430718_s_at	Mm.158247.1	AV313469	1700030A21Rik	2.523	4.190	5.120	1.661	2.030
1437327_x_at	Mm.11311.2	BB251327	2310057D15Rik	3.002	5.389	6.092	1.795	2.029
1448102_a_at	Mm.28437.1	NM_023191	2700038L12Rik	9.615	15.675	19.507	1.630	2.029
1452223_s_at	Mm.27621.1	BM214039	2900054P12Rik	3.079	4.075	6.247	1.323	2.029
1417135_at	Mm.8709.1	NM_009274	Srpk2	4.734	6.728	9.603	1.421	2.028
1424355_a_at	Mm.2137.1	AF038848	Sin3b	10.484	14.780	21.257	1.410	2.028
1431385_a_at	Mm.29791.2	AK002809	Mbtps1	0.702	0.854	1.424	1.216	2.028

1421781_at	Mm.4453.1	NM_009476	Upk2	0.507	0.559	1.028	1.102	2.027
1429042_at	Mm.32615.1	AK008415	2010200O16Rik	2.161	3.944	4.380	1.825	2.027
1426386_at	Mm.218533.1	BC003907	1500016H10Rik	4.429	4.708	8.977	1.063	2.027
1455392_at	Mm.40741.1	BB081437	9630019K15Rik	2.963	0.964	6.005	0.325	2.027
1457635_s_at	Mm.129481.1	BB096079	Nr3c1	0.578	0.562	1.170	0.972	2.026
1423254_x_at	Mm.30120.1	BB836796	Rps27l	24.860	31.550	50.354	1.269	2.026
1451052_at	Mm.34708.1	AA790871	2610019N19Rik	2.311	3.369	4.682	1.458	2.025
1452918_at	Mm.86894.1	AK011404	D19Ertd737e	4.299	6.854	8.708	1.594	2.025
1437109_s_at	Mm.28694.2	BE949068	Lsm6	7.565	6.294	15.321	0.832	2.025
1426474_at	Mm.41651.1	AI851949	Atpaf2	1.114	1.227	2.256	1.102	2.025
1438653_x_at	Mm.4098.3	AV309164	Sca10	30.391	52.286	61.535	1.720	2.025
1451169_at	Mm.22121.1	BC024503	D7Ertd156e	0.929	1.086	1.880	1.169	2.025
1451531_at	Mm.46542.1	BC018472	BC018472	0.512	0.627	1.037	1.225	2.025
1416269_at	Mm.1674.1	NM_020582	Atp5j2	29.690	39.967	60.105	1.346	2.024
1420899_at	Mm.22660.1	AW542340	Rab18	4.798	9.106	9.706	1.898	2.023
1450713_at	Mm.38496.1	NM_013884	Cspg5	18.485	19.084	37.398	1.032	2.023
1424483_at	Mm.29500.1	AV226922	4022402H07Rik	2.876	5.370	5.814	1.867	2.021
1450854_at	Mm.4742.1	BM232515	Pa2g4	1.408	1.801	2.844	1.279	2.020
1450865_s_at	Mm.7328.1	BF167852	Mrps24	3.618	5.295	7.305	1.463	2.019
1424139_at	Mm.212897.1	BC011105	Rap1a	12.132	20.695	24.493	1.706	2.019
1417855_at	Mm.19973.1	NM_133694	Fbxo37	1.325	1.682	2.676	1.269	2.019
1426351_at	Mm.1777.1	AI648003	Hspd1	25.347	31.422	51.160	1.240	2.018
1426483_at	Mm.4428.1	BF457576	Prkrir	5.504	6.845	11.106	1.244	2.018
1451540_at	Mm.181653.1	BC009068	Mpi1	2.152	3.217	4.340	1.495	2.017
1418171_at	Mm.182094.1	NM_025703	3930402F23Rik	10.371	17.998	20.914	1.735	2.017
1422208_a_at	Mm.4702.1	BC016135	Gnb5	2.183	3.742	4.403	1.714	2.017
1433496_at	Mm.184314.1	AV122321	2810024B22Rik	1.034	0.994	2.084	0.962	2.016
1429083_at	Mm.41348.1	BB006088	Agl	1.516	1.953	3.056	1.289	2.016
1456316_a_at	Mm.28214.2	BI965035	Acbd3	1.020	1.569	2.055	1.538	2.015
1460690_at	Mm.23826.1	BI526818	BC003940	5.541	7.825	11.158	1.412	2.014
1452160_at	Mm.21712.1	BB707122	Tiparp	1.760	3.414	3.543	1.940	2.014
1420485_at	Mm.142195.1	AW060738	Nol7	0.716	0.635	1.442	0.886	2.013
1460004_x_at	Mm.41462.3	BB492711	Stx6	4.885	8.386	9.834	1.717	2.013
1419506_at	Mm.36520.1	NM_010282	Ggps1	1.869	3.394	3.762	1.815	2.013
1429736_at	Mm.130694.1	BB659256	1110003F05Rik	1.453	1.273	2.924	0.876	2.012
1422457_s_at	Mm.24433.1	NM_019929	Smt3h1	9.566	14.116	19.247	1.476	2.012
1418658_at	Mm.46494.1	BC025158	2410005O16Rik	1.458	1.863	2.934	1.278	2.012
1452032_at	Mm.30039.2	BI687658	Prkar1a	17.357	25.985	34.910	1.497	2.011
1448476_at	Mm.2831.1	NM_008672	Nap1l4	11.652	14.534	23.429	1.247	2.011
1426516_a_at	Mm.28548.2	AK014526	Lpin1	3.011	4.230	6.053	1.405	2.010
1415826_at	Mm.27082.1	NM_133826	Atp6v1h	4.649	8.767	9.345	1.886	2.010
1448275_at	Mm.29647.1	AK018383	Tmem19	0.846	1.543	1.701	1.824	2.010
1438183_x_at	Mm.104920.3	AV253518	Sdh1	1.094	0.914	2.200	0.835	2.010
1427294_a_at	Mm.193091.1	BB406585	1810073N04Rik	4.951	7.314	9.952	1.477	2.010
1435863_at	Mm.30077.1	BE133720	Commd6	3.263	5.514	6.560	1.690	2.010
1418263_at	Mm.45108.1	BC024852	Ddx25	2.079	1.872	4.178	0.900	2.010
1438853_x_at	Mm.24495.3	BB463580	Ddx54	5.270	7.669	10.592	1.455	2.010
1424172_at	Mm.44240.1	BC004749	Hagh	2.506	3.835	5.035	1.530	2.009
1418002_at	Mm.29353.1	NM_025933	2010110M21Rik	19.544	34.718	39.264	1.776	2.009
1422717_at	Mm.26166.1	AW554436	Acp1	0.611	0.784	1.228	1.282	2.009
1424904_at	Mm.98366.1	BC019143	1300010F03Rik	1.057	1.459	2.123	1.380	2.008
1419257_at	Mm.218669.1	BC006022	Tcea1	4.380	7.774	8.796	1.775	2.008

1416699_at	Mm.29264.1	NM_026124	1110008F13Rik	14.358	17.992	28.826	1.253	2.008
1449214_a_at	Mm.31402.1	BC025160	Opa1	0.976	1.031	1.959	1.057	2.007
1441912_x_at	Mm.73650.1	AV290571	C2	0.995	0.255	1.998	0.256	2.007
1438159_x_at	Mm.2206.3	AV046532	Ndufv2	24.657	28.749	49.474	1.166	2.006
1442883_s_at	Mm.195873.1	BI413749	D10Bwg1364e	4.764	6.217	9.559	1.305	2.006
1434855_at	Mm.87319.1	AV298377	5730457F11Rik	2.465	4.305	4.946	1.746	2.006
1426388_s_at	Mm.3860.3	BG229030	Ryk	3.724	4.436	7.469	1.191	2.006
1451437_at	Mm.29044.1	BC019536	5033406L14Rik	0.959	0.820	1.923	0.855	2.005
1415863_at	Mm.525.1	NM_013507	Eif4g2	17.913	29.132	35.918	1.626	2.005
1428258_at	Mm.318.1	BG795140	2010107E04Rik	26.572	31.343	53.272	1.180	2.005
1455988_a_at	Mm.153159.2	BM210281	Cct6a	22.210	33.034	44.528	1.487	2.005
1418281_at	Mm.231.1	NM_011234	Rad51	1.765	0.514	3.539	0.291	2.005
1416559_at	Mm.36766.1	NM_025897	1500003O22Rik	1.094	1.654	2.193	1.511	2.004
1435372_a_at	Mm.4742.2	AI152156	Pa2g4	4.855	5.105	9.731	1.052	2.004
1451399_at	Mm.6560.1	BC008274	Brp17	6.268	6.320	12.561	1.008	2.004
1424332_at	Mm.28923.1	AF422144	Rab40c	0.610	0.932	1.223	1.527	2.004
1448677_at	Mm.22072.1	NM_010926	Noc4	5.519	9.480	11.060	1.718	2.004
1433462_a_at	Mm.117037.2	BB208212	Pi4k2a	0.559	0.744	1.120	1.331	2.003
1429809_at	Mm.37266.1	AK018506	8430438D04Rik	2.228	2.661	4.462	1.195	2.003
1428112_at	Mm.29778.1	AK014338	Armet	10.262	12.981	20.553	1.265	2.003
1429502_at	Mm.46007.1	BE533039	Stch	0.981	1.642	1.964	1.674	2.003
1439453_x_at	Mm.28690.3	AV259141	1500026D16Rik	4.045	3.641	8.098	0.900	2.002
1416484_at	Mm.3679.1	BB833716	Ttc3	7.575	10.189	15.164	1.345	2.002
1438161_s_at	Mm.16323.2	BB251459	Rfc4	1.722	0.574	3.446	0.334	2.001
1416186_at	Mm.29159.1	NM_026383	Pnrc2	7.712	12.441	15.425	1.613	2.000
1436813_x_at	Mm.34296.4	BB332580	Khsrp	3.156	5.236	6.312	1.659	2.000
1452896_at	Mm.196468.1	AK011217	Gtl3	4.107	5.264	8.214	1.282	2.000
